# Sexually divergent development of depression-related brain networks during healthy human adolescence

**DOI:** 10.1126/sciadv.abm7825

**Published:** 2022-05-27

**Authors:** Lena Dorfschmidt, Richard A. Bethlehem, Jakob Seidlitz, František Váša, Simon R. White, Rafael Romero-García, Manfred G. Kitzbichler, Athina R. Aruldass, Sarah E. Morgan, Ian M. Goodyer, Peter Fonagy, Peter B. Jones, Ray J. Dolan, NSPN Consortium, Neil A. Harrison, Petra E. Vértes, Edward T. Bullmore

**Affiliations:** 1 Department of Psychiatry, University of Cambridge, Cambridge CB2 0SZ, UK.; 2 Department of Psychiatry, University of Pennsylvania, Philadelphia, PA 19104, USA.; 3 Department of Child and Adolescent Psychiatry and Behavioral Science, University of Pennsylvania, Philadelphia, PA 19104, USA.; 4 Lifespan Brain Institute, Children’s Hospital of Philadelphia and Penn Medicine, Philadelphia, PA 19104, USA.; 5 Department of Neuroimaging, Institute of Psychiatry, Psychology and Neuroscience, King’s College London, London SE5 8AF, UK.; 6 The Alan Turing Institute, London NW1 2DB, UK.; 7 Department of Computer Science and Technology, University of Cambridge, Cambridge CB2 0SZ, UK.; 8 Research Department of Clinical, Educational and Health Psychology, University College London, London WC1E 6BT, UK.; 9 Cambridgeshire and Peterborough NHS Foundation Trust, Huntingdon PE29 3RJ, UK.; 10 Wellcome Trust Centre for Neuroimaging, University College London Queen Square Institute of Neurology.; 11 Max Planck University College London Centre for Computational Psychiatry and Ageing Research, University College London, London WC1B 5EH, UK.; 12 Department of Neuroscience, Brighton and Sussex Medical School, University of Sussex Campus, Brighton BN1 9RY, UK.; 13 Cardiff University Brain Research Imaging Centre, Cardiff University, Cardiff CF24 4HQ, UK.

## Abstract

Sexual differences in human brain development could be relevant to sex differences in the incidence of depression during adolescence. We tested for sex differences in parameters of normative brain network development using fMRI data on *N* = 298 healthy adolescents, aged 14 to 26 years, each scanned one to three times. Sexually divergent development of functional connectivity was located in the default mode network, limbic cortex, and subcortical nuclei. Females had a more “disruptive” pattern of development, where weak functional connectivity at age 14 became stronger during adolescence. This fMRI-derived map of sexually divergent brain network development was robustly colocated with i prior loci of reward-related brain activation ii a map of functional dysconnectivity in major depressive disorder (MDD), and iii an adult brain gene transcriptional pattern enriched for genes on the X chromosome, neurodevelopmental genes, and risk genes for MDD. We found normative sexual divergence in adolescent development of a cortico-subcortical brain functional network that is relevant to depression.

## Teaser

Sex differences in normative adolescent brain maturation are anatomically and genetically relevant to depression.

## INTRODUCTION

Adolescence is a period of critical development of the brain, characterized by changes in both structure (*1*–*4*) and function (*5*, *6*) that coincide with changes in cognition and behavior (*7*). It is also a time of increasing incidence of many psychiatric disorders, including depression, which occurs more frequently in females than males (*8*, *9*). Small sex differences in mood have been reported from the age of 11, and by the age of 15, females are about twice as likely to be depressed as males (*8*–*10*). Recent work has supported the idea that sexually divergent risk for mood disorders could be related to sex differences in adolescent brain network development (*11*).

Functional brain networks derived from resting-state functional magnetic resonance imaging (fMRI) can be used to study complex network organization in the brain. Each node of these networks is an anatomical region, and each edge weight is an estimator of association, so-called functional connectivity, typically the correlation or coherence between the two fMRI signals simultaneously measured for each possible pair of nodes in the network (*12*, *13*).

The brain is plastic and undergoes maturational changes throughout life. Primary sensory and motor areas mature most rapidly during childhood, while association areas undergo their most profound changes during adolescence (*6*, *14*, *15*). Previous resting-state fMRI studies have reported a shift from local to distributed networks (*16*) and an increase in the strength of long-range connections (*17*, *18*) in the course of adolescence. However, it has since been noted that in-scanner head motion may have confounded many of the effects previously attributed to age, particularly in younger participants (*19*–*21*). Developmental imaging studies have therefore used different strategies to address these concerns, e.g., by restricting analysis to motion-uncontaminated subsamples of data acquired with no detectable head motion (*6*) or by regressing each nodal fMRI signal on the global average fMRI signal, aka global signal regression (GSR) (*22*). Issues concerning optimal head motion correction for preprocessing fMRI data remain controversial (*23*–*25*).

It is not yet clear how FC differs between males and females, either during adolescence or adulthood. One widely reported sex difference is increased FC of the default mode network (DMN) in females (*26*–*30*). Female-increased (or female > male) connectivity has also been reported in the subcortex and limbic areas (cingulate gyrus, amygdala, and hippocampus) (*31*), whereas male > female connectivity has been reported for sensorimotor areas (*26*, *29*, *31*). However, these effects are not consistently found across studies (*27*, *28*, *32*). Most research on sex differences has focused on preselected regions, often including the amygdala (*33*, *34*), with few studies having investigated sex differences comprehensively over all brain regions (*26*, *29*, *35*–*37*). Most prior fMRI studies of brain development have focused on estimating “average” effects of age across both sexes, e.g., by including sex as a covariate in the statistical model for estimation of developmental parameters, and few prior studies have reported age-by-sex interactions or the conditioning of developmental parameters by sex (*29*, *31*).

We start from the position that the sexually divergent risk trajectory for depression, with higher depressive symptom scores for adolescent females than males (*8*, *9*), could be the psychological or clinical representation of underlying sex differences in adolescent brain network development (*28*, *29*, *35*). Specifically, we hypothesized (i) that there are normative sex differences in adolescent FC development and (ii) that these sex differences in functional brain network maturation are anatomically, psychologically, and genetically relevant to depression. To investigate these overarching hypotheses experimentally, we used a two-step analytic approach: First, we identified sexually divergent systems of healthy adolescent brain development, and second, we tested the anatomical colocation of sexually divergent fMRI systems with prior maps of task-related brain activation, human brain gene expression, and depression-related abnormalities of functional dysconnectivity. To assess the diagnostic specificity of the depression-related results from the second stage of analysis, we repeated some of these analyses using comparable, schizophrenia-related fMRI and genetic data.

Using fMRI data from a previously published (*6*) accelerated longitudinal study (*N* = 298; age range 14 to 26 years; 51% female; Table 1), stratified by age and balanced for sex per age stratum (*38*), we estimated the effects of sex on three parameters of adolescent development of resting-state FC: (i) baseline connectivity at age 14, FC_14_, (ii) the adolescent rate of change in connectivity, FC_14 − 26_, estimated at nodal and edgewise levels of analysis, and (iii) the maturational index for each node, MI, which is the signed correlation coefficient between FC_14_ and FC_14 − 26_ over all edges connecting a given node to the rest of the network. The sign of MI (positive or negative) has been reported to reflect two distinct modes of adolescent development of brain FC, operating in distinct sets of brain regions (*6*): (i) Positive MI is indicative of conservative development, meaning strong connections at 14 years became stronger during adolescence; (ii) negative MI is indicative of disruptive development, meaning weak connections at 14 years became stronger during adolescence and strong connections became weaker. Disruptive development is thus characteristic of nodes that change the relative strength or rank order of their functional connections to the rest of the brain network (see fig. S1) over the course of adolescence; see Materials and Methods and Discussion for detail. MI is an appealing measure for estimating developmental changes due to its succinct characterization of systems level maturational changes across all of a node’s edges and because it relates the magnitude of developmental change during adolescence to the strength of FC at baseline. The sex difference in MI (ΔMI) thus summarizes the sexual divergence in how a node’s edgewise wiring changes from baseline during adolescence and thereby goes beyond traditional analyses of age-by-sex interactions by highlighting sex differences in regions that are likely undergoing rewiring of their functional connections (“disruptive”) and regions that are further consolidating their functional connections over the course of adolescence in line with their previous trend (“conservative”).

**Table 1.** Adolescent developmental MRI sample. Total *N* = 298 healthy young people participated in an accelerated longitudinal MRI study, with recruitment balanced for sex in each of five age-defined strata and each subject scanned between one and three times (with follow-up scans taking place approximately 6 and 18 months after baseline). FD, a measure of head movement in millimeters, was significantly greater in males compared to females on average over all ages and in the youngest two age strata specifically (*P* < 0.05, uncorrected; fig. S3).

	**Sex**	**Age stratification**					**All ages**
		**14–15**	**16–17**	**18–19**	**20–21**	**22–25**	
*N* subjects	Female	22	151	24	32	22	151
	Male	32	33	24	35	23	147
FD (mm)	Female	0.13*	0.10*	0.12	0.10	0.13	0.11*
	Male	0.15*	0.13*	0.12	0.14	0.13	0.13*
*N* scans/subject (1|2|3)	Female	9|22|3	11|25|3	6|16|2	14|16|2	14|7|1	54|86|11
	Male	7|24|1	8|24|1	7|16|1	11|20|4	8|14|1	41|98|8

In relation to our first hypothesis, we found that there was a sex-related difference in adolescent brain network development: Females had significantly more disruptive development of FC in a default mode cortical, limbic, and subcortical network. In relation to our second hypothesis, we found that this developmentally divergent brain system was colocated with loci of brain activation by reward-related tasks, with expression of a weighted function of the whole genome enriched for X chromosome genes, genes expressed during various phases of brain development, and genes identified by genome-wide association with major depressive disorder (MDD), and colocated with an anatomical map of depression-related differences in FC from an independent case-control fMRI study of MDD (*39*, *40*). The robustness of all these results to potentially confounding effects of sex differences in head movement, intracranial volume, or global FC was evaluated (and supported) by five sensitivity analyses; see the “Sensitivity analyses” section in Supplementary Text. Last, we assessed the diagnostic specificity of the relationships between sexually divergent brain development (indexed by the ΔMI map) and (i) MDD case-control differences in FC and (ii) brain expression profiles of MDD risk genes. To do this, we repeated analyses (i) and (ii) using comparable data from prior independent studies of schizophrenia. Specifically, we tested for significant association between ΔMI and schizophrenia-related differences in FC estimated from The Centre for Biomedical Research Excellence (COBRE) case-control fMRI study of schizophrenia (*41*, *42*), and we tested for significant enrichment of the list of genes transcriptionally colocated with the ∆MI map by risk genes for schizophrenia identified by a large prior genome-wide association study (GWAS) (*43*). Thus, we conclude that normative sexual divergence in adolescent development of FC in a cortico-subcortical brain network is anatomically, genetically, and psychologically relevant to depression.

## RESULTS

### Analyzable sample, head movement, and sensitivity analyses

A total of 36 scans were excluded by quality control criteria including high in-scanner motion [mean framewise displacement (FD) > 0.3 mm or maximum FD > 1.3 mm], co-registration errors, or extensive fMRI dropout. The analyzable sample thus consisted of 520 scans from 298 participants (151 females; Table 1 and table S1), with regional signal available in 346 cortical and subcortical regions (fig. S2). Males had significantly more head movement than females in the youngest two age strata (*P* < 0.05, uncorrected) and on average over all ages (Table 1 and figs. S3 and S4).

After preprocessing for within-subject correction of head motion effects on individual fMRI time series, FC was positively correlated with individual differences in mean FD and this effect scaled with distance between the nodes (fig. S4A). We therefore also corrected for between-subject differences in head motion by regressing each interregional correlation on mean FD across all participants. This removed the relationship between connectivity and FD, as well as the distance dependence in this relationship (fig. S4D) (*19*, *44*).

### Age and sex effects on FC

**Fig. 1.**
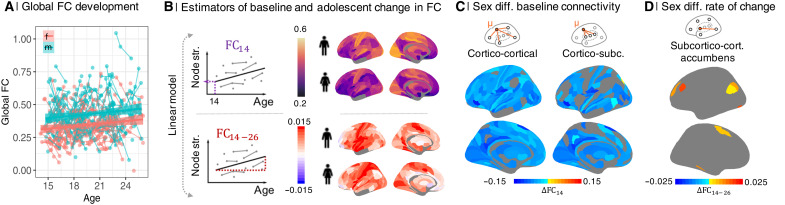
Sex differences in FC at age 14 (FC_14_) and adolescent rate of change of connectivity (FC_14−26_) per year. (**A**) Global functional connectivity (FC) strength increased with age [*t*(219) = 2.3, *P* < 0.05] and was higher in males [*t*(296) = 5.5, *P* < 0.0001]. (**B**) To estimate two parameters of development at each regional node, we fit a linear model to the relationship between age and weighted degree (nodal strength of connectivity to the rest of the network) for males (m) and females (f) separately. The two model parameters are the intercept, or “baseline” connectivity at age 14 (FC_14_), and the linear rate of change in connectivity during adolescence (FC_14 − 26_). (**C**) We found that 321 of 330 regions had significantly increased cortico-cortical connectivity and 230 of 330 regions had increased cortico-subcortical connectivity (*P*_FDR_ < 0.05) at baseline (FC_14_) in males. (**D**) FC_14 − 26_ was only significantly different between sexes, decreased in females, in 27 of 330 subcortico-cortical connections of the nucleus accumbens.

We modeled age and sex effects on global FC of each participant, estimated as mean weighted degree, using linear mixed effects models (LMEs). FC increased with age [*t*(219) = 2.3, *P* < 0.05], and males had higher global mean weighted degree than females [*t*(296) = 5.5, *P* < 0.0001] (Fig. 1A and table S2).

**Fig. 2.**
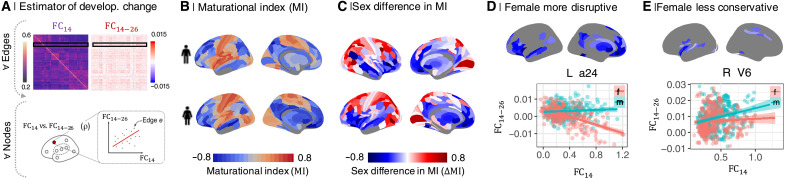
Sex differences in MI. (**A**) The MI was estimated as the correlation between edgewise baseline connectivity at age 14 (FC_14_) and the adolescent rate of change in connectivity per year (FC_14 − 26_) at each regional node. (**B**) MI maps for males and females separately. MI was generally negative (blue) in frontal and association cortical areas and positive (orange) in primary motor and sensory cortices. (**C**) The sex difference in MI, ΔMI = MI_female_ − MI_male_, was significant in 230 of 346 regional nodes (*P*_FDR_ = 0.05). ΔMI was significantly negative in the ventral and medial prefrontal gyrus, ventrolateral prefrontal cortex, anterior and posterior cingulate gyrus, medial temporal gyrus, and subcortical nuclei (table S3), indicating sex differences in adolescent development of connectivity of these regions. More specifically, negative ΔMI defined a set of brain regions where adolescent development was either more disruptive (weak connections at 14 years became stronger during adolescence, and strong connections became weaker) or less conservative (strong connections at 14 years became stronger or weak connections became weaker during adolescence) in females compared to males. (**D**) Map of brain regions where development was more disruptive in females. As exemplified by the left area 24 (L a24), functional connections of disruptively developing nodes that were strong at 14 years (high FC_14_, *x* axis) became weaker over the period 14 to 26 years (FC_14 − 26_ < 0, *y* axis), and edges that were weakly connected at 14 years became stronger over the course of adolescence, especially in females. (**E**) Map of brain regions where development was less conservative in females. As exemplified by right visual area V6 (R V6), connections that were strong at baseline become stronger over the period 14 to 26 years, especially in males.

Regional FC was estimated between and within cortical and subcortical subsets of nodes by averaging the relevant parts of the connectivity matrix (fig. S5). To model development of FC during adolescence, we focused on three parameters: regional baseline connectivity at age 14, FC_14_, regional linear change in connectivity between 14 and 26 years, FC_14 − 26_ (Fig. 1B), and the signed Spearman correlation of these two parameters, termed MI (−1 < MI < + 1; Fig. 2A) (*6*). Previous work on this sample has reported developmental change (controlling for sex) in terms of these parameters estimated at each regional node of a whole-brain fMRI network (*6*). Here, we estimated each of these parameters for males and females separately and the between-sex difference for each parameter, e.g., ΔMI = MI_female_ − MI_male_. We tested the significance of the between-sex difference in each parameter at each regional node using parametric tests (see the “Analysis of sex effects on parameters of adolescent brain development” section in Supplementary Text).

Baseline connectivity at age 14 was quantitatively (but not significantly) greater in primary sensorimotor cortex than in association cortex for both sexes (Fig. 1B and figs. S6A, S7, and S8). As predicted by the sex difference in global FC at all ages (Fig. 1A), males had significantly stronger baseline connectivity than females at 14 years, i.e., ΔFC_14_ = FC_14, female_ − FC_14, male_ < 0, in cortico-cortical and cortico-subcortical connections (Fig. 1C).

The pattern of adolescent rate of change in connectivity was strongly positive in sensorimotor cortex and was quantitatively (but not significantly) less positive or slightly negative in association cortical and limbic areas, for both sexes. After correction for multiple comparisons, there was no significant sex difference (at *P*_FDR_ < 0.05), i.e., the null hypothesis that ΔFC_14 − 26_ = 0 was not refuted, for cortico-cortical or cortico-subcortical connectivity, but a subset of 27 subcortico-cortical connections, involving the nucleus accumbens, had significantly less positive rates of change in females compared to males (*P*_FDR_ < 0.05; Fig. 1D and figs. S6A, S9, and S10).

MI was positive in sensorimotor cortex and negative in association cortex and subcortical areas, in both sexes separately (Fig. 2B and fig. S11), as previously reported for both sexes on average (*6*). However, there were many areas of significant sex difference in MI (*P*_FDR_ < 0.05; fig. S11). Females had more negative MI than males in 107 regions (Fig. 2B; for a full list, see table S3; effect sizes are shown in fig. S12). In 84 of these regions, exemplified by the left area 24 (Fig. 2D), there was more disruptive development in females, i.e., weak connections at 14 years became stronger during adolescence and strong connections became weaker in females compared to males. In 23 regions, exemplified by the right visual area V6 (Fig. 2D), there was less conservative development in females, i.e., strong connections at 14 years became stronger during adolescence in males compared to females (figs. S13 and S14). Thus, the brain system defined by regions with a negative ΔMI is predominantly characterized by a weak-getting-stronger profile of developmental change in FC that was greater in females than males.

**Fig. 3.**
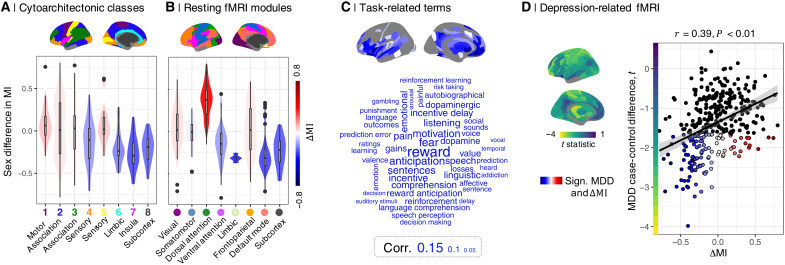
Sex difference in MI in psychological and psychiatric context. (**A**) ΔMI was most negative in cytoarchitectonically defined secondary sensory, limbic, and insula cortex and subcortical structures (**B**) as well as functionally defined (fMRI) DMN, ventral attention network, limbic systems, and subcortical structures. (**C**) Word cloud of NeuroSynth meta-analytical cognitive terms scaled according to their strength of association with the disruptively developing brain regions (cortical map of ΔMI < 0). (**D**) Scatterplot of MDD case-control *t* statistics (*y* axis) versus ΔMI (*x* axis). Each point represents one of 346 cortical or subcortical regions; regions of interest that show a significant MDD case-control difference, *t* ≠ 0, and a significant sex difference in MI, *t* ≠ 0, are highlighted. The fitted line and 95% confidence interval indicate the positive correlation (*r* = 0.4, *P* < 0.001, *P*_spin_ < 0.001) between the spatial maps of MDD case-control differences, *t*, and ΔMI, shown alongside the *y* and *x* axes, respectively. Regions with sexually divergent disruptive development in adolescence (negative ΔMI) had reduced degree of connectivity (negative *t*) in adult MDD cases.

The unthresholded map of ΔMI was co-registered with a prior map of cortical cytoarchitectonic classes (Fig. 3A) and a prior map of resting state networks (*45*) from an independent component analysis (ICA) of adult fMRI data (Fig. 3B). Regions of negative ΔMI were concentrated in secondary sensory, limbic, and insular classes of cortex; in subcortical structures, defined anatomically (Fig. 3A); and in default mode, limbic, ventral attentional, and subcortical systems defined functionally (Fig. 3B).

It has recently been proposed that many aspects of brain organization conform to a gradient or axis between unimodal and transmodal cortical areas (*46*). We found that the map of MI (*6*), estimated on average over both sexes, was significantly negatively correlated with a prior map of the unimodal-transmodal axis (U-T axis) (*46*) such that positive MI (conservative development) was associated with unimodal regions and negative MI (disruptive development) was associated with transmodal regions (*r* = −0.63, *P* < 0.01, *P*_spin_ < 0.01); see fig. S15A. The map of ΔMI, representing sex differences in MI, was also negatively correlated with the U-T axis map, such that transmodal regions tended to have more negative ΔMI, indicating more disruptive development in females (*r* = −0.28, *P* < 0.01, *P*_spin_ = 0.08); see fig. S15B. However, the nominally significant correlation between the ΔMI map and the U-T axis map was not robust to significance testing using the spin-test method to control for spatial autocorrelation in the maps, and the correlation between the ΔMI map and the U-T axis map was evidently not as strong as the correlation between the MI map and the U-T axis map.

Automated meta-analytic referencing of the unthresholded map of negative ΔMI was conducted using the NeuroSynth database of task-related fMRI activation coordinates (*47*). This indicated that regions with more disruptive (or less conservative) development in females were typically activated by tasks related to reward processing, emotion, motivation, incentive delay, and dopamine (Fig. 3C; for results for positive ΔMI, see fig. S16).

### Sexually divergent brain network development and gene expression

**Fig. 4.**
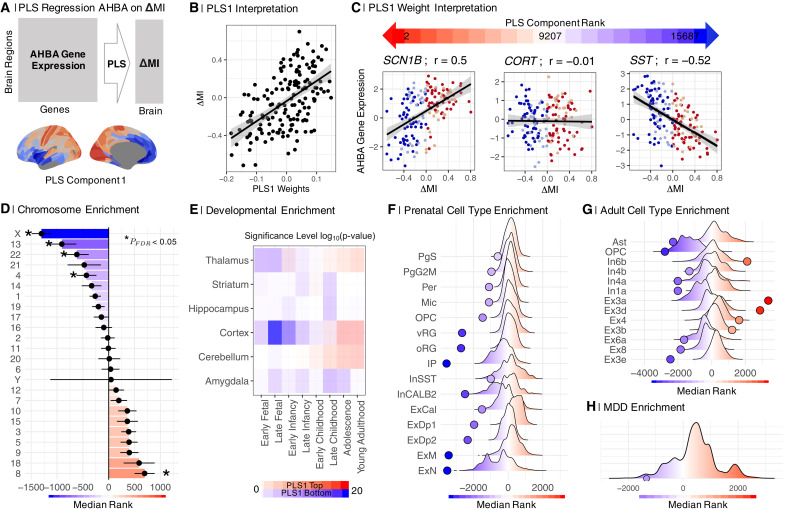
Sexually divergent, disruptive brain systems are colocated with brain tissue transcripts enriched for X chromosome, neurodevelopmental, and MDD risk genes. (**A**) We used PLS regression to map gene expression data (*43*) onto ΔMI. (**B**) PLS1 was positively correlated with ΔMI; thus, low PLS1 scores were colocated with low ΔMI or predominantly female more disruptive regions. (**C**) Relationship of ΔMI to expression of exemplary genes: sodium voltage-gated channel beta subunit 1 (*SCN1B*), a positively weighted gene near the top of the ranked PLS1 weights list; cortistatin (*CORT*), a near-zero weighted gene in the middle of the list; and somatostatin (*SST*), a negatively weighted gene near the bottom. Negatively weighted genes were more strongly expressed in regions of negative ΔMI, that is, predominantly female > male disruptive regions, whereas positively weighted genes were more strongly expressed in regions with female > male conservative development indicated by positive ΔMI. (**D**) Enrichment analysis for chromosomal genes. Plot of median ranks of genes from each chromosome on PLS1. (**E**) Enrichment analysis for neurodevelopmental genes. Negatively weighted genes were enriched for genes expressed in cortex during late fetal and early postnatal development and for genes expressed in the amygdala, hippocampus, and striatum during late childhood and adolescence. Positively weighted genes were enriched for genes typically expressed in cortex and cerebellum during adolescence and early adult life. (**F**) Enrichment analysis for prenatal cell type–specific genes. Negatively weighted genes (blue) were significantly enriched for genes expressed by prenatal radial glia (vRG and oRG), microglia (Mic), oligodendrocyte progenitor cells, and excitatory neurons. (**G**) Enrichment analysis for adult cell type–specific genes. Negatively weighted genes were significantly enriched for genes expressed by adult astrocytes, oligodendroglial precursor (OPC) cells, and excitatory neurons. (**H**) Enrichment analysis for MDD-related genes. Negatively weighted genes were enriched for genes associated with major depressive disorder by an independent GWAS (*52*).

To investigate the relationships between gene expression profiles and sexually divergent adolescent brain development, we used partial least squares (PLS) regression to find the weighted gene expression pattern that was most closely colocated with the ΔMI map (Fig. 4A) (*5*, *14*, *48*). Whole-genome transcripts were estimated for the average of each of 180 bilaterally homologous cortical regions using adult postmortem data (*N* = 6) provided by the Allen Human Brain Atlas (*49*).

The first PLS component (PLS1; Fig. 4A) explained 34.6% of the variance in ΔMI, significantly more than expected by chance (*P*_perm_ < 0.05, *P*_spin_ < 0.05). The PLS1 gene expression weights were positively correlated with ΔMI. This means that negatively weighted genes, at the bottom of the ranked PLS1 list, were overexpressed in regions with negative ΔMI, or more disruptive maturational change in females. Conversely, positively weighted genes, at the top of the ranked PLS1 list, were underexpressed in regions with negative ΔMI (Fig. 4B).

To test the hypothesis that sex chromosomal gene expression, particularly expression of X chromosome genes, was related to the sexual differences in adolescent brain development, we assessed chromosomal enrichment of the genes on PLS1. We hypothesized that gene expression patterns related to sex differences in adolescent brain development might be enriched for X chromosomal genes. First, genes on the X chromosome are enriched for sex-differential gene expression in multiple tissues, including the prenatal (*50*) and postnatal brain (*51*). Furthermore, the X chromosome is diploid in females (XX) and haploid in males (XY), and while X chromosome inactivation silences transcription of one of the two X chromosomes in females, incomplete inactivation has been shown to affect at least 23% of X chromosomal genes, which results in sex biases in gene expression and is likely to introduce phenotypic diversity (*52*). We found that the most negatively weighted genes, which were highly expressed in brain regions that demonstrated more disruptive development in females, i.e., regions with negative ΔMI or more negative MI in females, were most strongly enriched for X chromosome genes (*P*_perm_< 0.001; Fig. 4C, fig. S17, and table S4).

Regional differences in cortical gene expression have been attributed to different proportions of functionally specialized neuronal, glial, and other cell types in different cortical areas (*53*). We therefore used the Cell Type–Specific Enrichment Analysis (CSEA) tool (*54*) to assess cell type enrichment of the most positively and negatively weighted genes on PLS1. We found that negatively weighted genes (*Z* < −2.58) were enriched for genes with cortical expression in late fetal and early postnatal life and for genes with amygdala, hippocampal, and striatal expression in late childhood and adolescence (Fig. 4D). In contrast, positively weighted genes (*Z* > 2.58) were enriched for genes with cortical, cerebellar, and thalamic expression during adolescence and young adulthood (Fig. 4D). These results indicate that the negatively weighted genes, most strongly expressed in cortico-subcortical regions that demonstrated more disruptive development in females, i.e., regions with negative ΔMI or more negative MI in females, were specialized for perinatal development of cortex and later development, in childhood and adolescence, of subcortical structures.

We further explored developmental aspects of the sexually divergent system by testing for enrichment by genes specific to prenatal and postnatal cell types (*55*, *56*). We found that negatively weighted genes, which were overexpressed in cortico-subcortical regions that demonstrated more disruptive development in females, i.e., regions with negative ΔMI or more negative MI in females, were enriched for prenatal cell types (*54*), including oligodendroglial precursor cells, microglia, astrocyte progenitor radial cells, inhibitory and excitatory cortical neurons (Fig. 4E and table S5), as well as for multiple adult glial and neuronal cell classes (Fig. 4F, fig. S18, and table S6).

### Sexually divergent brain network development and depression

Extending the enrichment analysis to consider depression-related genes, we found that the list of genes strongly coexpressed with sexually divergent disruptive brain systems was significantly enriched for risk genes for MDD (Fig. 4H and table S7) (*57*). For this analysis, risk genes for MDD were defined by a prior study, in which single-nucleotide polymorphisms (SNPs) significantly associated with MDD in one of the largest available GWASs (*58*–*60*) were mapped to functionally relevant genes using epigenetic (Hi-C) data to guide the interpretation of GWAS-significant SNPs in noncoding loci (*57*). More than 80% of risk variants identified by GWAS are found in the noncoding genome, which makes the interpretation of underlying biological mechanisms challenging. Noncoding SNPs can regulate distal genes via long-range regulatory interactions since the three-dimensional structure of the genome allows for distal enhancers to be brought into contact with sequentially distant promoters. Our enrichment results showed that the MDD risk genes were negatively weighted and ranked toward the bottom of the PLS1 list, indicating that they were more highly expressed in brain regions with disruptive development, indexed by negative ΔMI.

To assess the anatomical correspondence between the sexually divergent disruptive brain system and mood disorder–related changes in fMRI connectivity, we used resting state fMRI data from a prior case-control study of adult MDD cases (*N* = 50) and healthy controls (*N* = 46); see table S8. The parcellated, unthresholded map of MDD case-control differences in weighted degree (comprising 346 regional *t* statistics) was significantly colocated with the identically parcellated, unthresholded map of ΔMI (*r* = 0.4, *P* < 0.001, *P*_spin_ < 0.001; Fig. 3D). Brain regions with sexually divergent development in adolescence (negative ΔMI) had reduced degree of FC in MDD cases compared to controls. Perhaps, as expected, given that MI is estimated for each regional node as the correlation between FC_14_ and FC_14 − 26_ over all edges connecting the node in question to the rest of the network, we also found a correlation between the MDD case-control map and sex differences in baseline FC (∆FC_14_) and sex differences in the adolescent rate of change of connectivity (∆FC_14–26_; see fig. S19).

### Sensitivity analyses

To assess the robustness of key results to the two-step process for head motion correction, we conducted three sensitivity analyses (see the “Sensitivity analysis” section in Supplementary Text): (i) Sex-specific motion correction. FC matrices were regressed on FD separately for males and females (figs. S20 to 24). (ii) GSR correction. The fMRI time series at each node were regressed on the global fMRI signal per participant (figs. S25 to 28). (iii) Motion-matched subsample analysis. We used a subset of data (*N* = 314), comprising equal numbers of males and females, for which there was no statistical difference in FD (figs. S29 to S33).

Given the male > female sex difference in intracranial volume (*61*, *62*), we ran two further sensitivity analyses: (iv) Intracranial volume correction. We regressed global and edgewise fMRI metrics on intracranial volume estimated from structural MRI data on the same sample (figs. S34 to S37). (v) Global FC correction. We regressed edgewise fMRI metrics on global FC (figs. S38 to S41).

Results of these five sensitivity analyses were quantitatively compared to the corresponding results at all stages of the principal analysis. There was a significant correlation between the developmental fMRI metrics (FC_14_, FC_14 − 26_, and MI) estimated by each of these sensitivity analyses, and the same parameters estimated by our principal analysis (fig. S42). The key findings of sexually divergent adolescent development of FC between DMN, limbic, and subcortical regions (mean correlation *r* = 0.8 between principal and sensitivity analyses of ΔMI); gene expression enrichment for MDD-related genes; and colocation with MDD case-control dysconnectivity, were conserved in all five sensitivity analyses. In all sensitivity analyses, X chromosome genes ranked toward the bottom of PLS1; however, this enrichment was not always significant at *P* < 0.05. The cell-specific enrichment was largely conserved across all sensitivity analyses, with PLS1 consistently enriched for excitatory neurons. In particular, the potentially confounding effect of cortical volume on FC (*62*) did not appear to significantly alter our results; see figs. S20 to S41 for figures corresponding to Figs. 1 to 4 for each sensitivity analysis.

### Diagnostic specificity

To assess the specificity of the relationships between sexually divergent brain development (indexed by the ΔMI map) and (i) MDD case-control differences in FC and (ii) brain expression profiles of MDD risk genes, we repeated these analyses using comparable independent data on schizophrenia. First, we tested the colocation of the ΔMI map with a map of FC differences in schizophrenia cases (*N* = 67) compared to healthy controls (*N* = 81), reported in a prior case-control resting-state fMRI study (*41*, *42*); see the “Diagnostic specificity” section in Supplementary Text. We found that schizophrenia case-control differences in weighted degree were not significantly colocated with the unthresholded map of ΔMI (*r* = 0.05, *P* = 0.35, *P*_spin_ = 0.47); see fig. S43B. Second, we tested the list of genes transcriptionally colocated with ΔMI for enrichment by schizophrenia-related risk genes. We used the largest currently available GWAS of schizophrenia, which identified 270 schizophrenia-associated SNPs and mapped these SNPs to genes using epigenetic information (*43*). We found that genes transcriptionally colocated with ΔMI were not significantly enriched for genes associated with schizophrenia (*P* = 0.25); see fig. S43C.

## DISCUSSION

This study was motivated by the twin hypotheses that there are sex-divergent differences in brain functional network development of healthy adolescents and that these normative developmental differences are located in cortical areas and subcortical nuclei that are psychologically, genomically, and clinically relevant to depression. In this accelerated longitudinal fMRI study of healthy young people, we first identified human brain systems that demonstrated a significantly different pattern of adolescent development in females compared to males. We found sex differences in several aspects of FC: Females had lower global mean FC across all ages and reduced nodal strength of connectivity in most regional nodes at 14 years, FC_14_. However, there were more anatomically specific sex differences in two developmentally sensitive parameters: The rate of change in FC during adolescence, FC_14 − 26_, was significantly reduced in females for connections between one cortical nucleus (nucleus accumbens) and 27 cortical structures, and the MI, a coefficient of the linear relationship between edgewise FC_14_ and FC_14 − 26_ at each node, was significantly more negative in females for 107 cortical areas concentrated in the DMN, ventral attentional, and limbic networks, as well as subcortical nuclei.

The MI can be used to define two modes of adolescent brain functional network development (*6*). A conservative node is defined by a positive MI, indicating that it is highly connected or “hub-like” at baseline (14 years) and becomes even more strongly connected over the course of adolescence (14 to 26 years). Theoretically, conservative nodes could also be weakly connected at baseline and become even more weakly connected during adolescence; however, empirically, we found that this was not the case (fig. S14). A disruptive node is defined by a negative MI, indicating either that it is weakly connected at age 14 but becomes more strongly connected or hub-like during adolescence or that it is a strongly connected node at 14 years but becomes more weakly connected or less hub-like during adolescence. The disruptive developmental profile of weak-getting-stronger during adolescence hypothetically represents a “rewiring” in the functional connectome, which could be relevant to the acquisition of social, cognitive, and other skills (*6*). Similar selective strengthening of connections has also been observed on the cellular level in the developing *Caenorhabditis elegans* connectome (*63*). It has also been argued that brain networks that are most developmentally active during adolescence are most likely to contribute to the coincidentally increased risk of mental health symptoms, i.e., “moving parts get broken” (*11*). For these reasons, our analysis focused particularly on sexual differences in weak-getting-stronger disruption in cortico-subcortical networks; results for strong-getting-stronger or conservative development are summarized in fig. S16.

The first explanation that we considered for this sex difference in developmental fMRI parameters is that they were attributable to sex differences in potentially confounding variables, including head motion during scanning. Head movement is known to be a potentially problematic confounder in developmental fMRI (*19*–*21*), and males, especially younger males, had more head movement than females in this sample. We initially addressed this issue by a two-stage preprocessing pipeline that statistically corrected each participant’s functional connectome for between-subject differences in head motion, indexed by FD. These preprocessed data passed the standard quality control criteria for movement-related effects on FC. In addition, we conducted three sensitivity analyses of head movement, repeating the entire analysis for male and female data separately, for a “motion-matched” subset of the data in which there was no significant sex difference in FD, and for all data after GSR (figs. S20 to S33) (*24*). In parallel, we conducted two additional sensitivity analyses to assess whether the male > female differences in intracranial volume, or global FC, might have confounded our principal results. In all five sensitivity analyses, our key results were qualitatively and quantitatively conserved, e.g., ΔMI maps estimated by the principal analysis were strongly correlated (mean *r* ∼ 0.8) with corresponding maps estimated by each sensitivity analysis. We therefore consider that sex differences in head movement, intracranial volume, and global FC can be discounted as sufficient explanations for sex differences in these parameters of brain network development.

An alternative explanation is that sex differences in FC_14 − 26_ and MI reflect divergent development of specific cortico-subcortical circuits. In particular, females have a significantly more disruptive pattern of adolescent development, indexed by negative ΔMI, because functional connections that were weak at 14 years became stronger, and connections that were strong became weaker, over the course of adolescence. This sex difference in terms of FC could be related to sex differences in an underlying process of reconfiguration or remodeling of cortico-subcortical connectivity at a synaptic or neuronal scale. To assess the plausibility of this biological interpretation, we used preexisting data on human brain gene expression, and the dimension-reducing multivariate method of PLS to identify the set of genes that were most over- or underexpressed in brain regions corresponding to the divergent system defined by developmental fMRI. Enrichment analysis demonstrated that the genes that were most strongly expressed in brain regions with more disruptive (or less conservative) development in females included significantly more X chromosome genes than expected by chance. The same set of genes was also significantly enriched for genes that are known a priori to be expressed in cortical areas during early (perinatal) development and in subcortical structures, such as amygdala, during adolescent development.

Sexual differentiation of the brain has been proposed to occur in two stages: an initial “organizational” stage before and immediately after birth and a later “activational” stage during adolescence (*64*). It has long been argued that these events are driven by gonadal hormones. However, more recent work suggests a complex interplay of sex chromosomes and their downstream products leading to sexual differentiation of brain cells (*65*–*67*). The results of our enrichment analysis, indicating colocation of the sexually divergent fMRI-derived map with brain regions enriched for expression of X chromosomal and neurodevelopmental genes, are compatible with interpretation of adolescent change in fMRI connectivity as a marker of an underlying program of transcriptional changes in genes previously linked to postnatal sexual differentiation at a neuronal level.

We assessed the relevance to depression of this sexually divergent profile of brain network development in several ways. Anatomically, the DMN and subcortical structures that had more disruptive development in females, e.g., ventral medial prefrontal cortex, medial temporal gyrus, and anterior and posterior cingulate cortex, have previously been implicated as substrates of depressive disorder (*68*, *69*). This anatomical convergence was quantified by the significant spatial correlation between the whole brain map of sex differences in MI and an independent map of MDD case-control differences in nodal degree of FC. Cortical and subcortical areas with reduced degree of connectivity or “hubness” in MDD cases had more disruptive development in adolescent females. Genomically, the list of genes transcriptionally colocated with this divergently developing network was enriched for risk genes from prior genome-wide association studies of MDD. Further contextualizing the genes that were found to be significantly overexpressed in regions displaying more disruptive development in females, we noticed that this list included two (*SST* and *NPY*) of three genes previously reported (*70*), as specifically expressed by adult neuronal and glial cells and linked to neuroimaging phenotypes of depression (fig. S44). It is also notable that MDD has been previously associated with up-regulation of X-linked escapee genes and genes that control X-inactivation (*71*). Psychologically, by meta-analysis of a large prior database of task-related fMRI studies, we found that brain regions comprising the sexually divergent system were psychologically specialized for reward- and emotion-related processes that are fundamental to core depressive symptoms, e.g., anhedonia. Collectively, these results do not prove that there is a causal relationship between sexually divergent brain development and risk of depression. However, they demonstrate that there is a sexually divergent process of adolescent development of a cortico-subcortical system that is anatomically, genomically, and psychologically relevant to depression. These insights motivate and focus future studies purposively designed to test the hypothesis that sexual divergence of adolescent brain development causes contemporaneous or subsequent sex differences in the risk for mood disorders.

It is increasingly recognized that clinical phenotypes and genetic and environmental risk factors may be shared in common between depression and other mental health disorders arising in adolescence (*72*, *73*). In particular, abnormalities in fMRI connectivity have been reported as trans-diagnostic phenotypes, characteristic of multiple, diagnostically distinct disorders (*72*), and risk genes associated with individual mental health and neurodevelopmental disorders have been found to overlap across disorders, implying that some genes confer trans-diagnostic risk for multiple neuropsychiatric disorders (*73*). In this context, it is reasonable to ask whether the significant associations that we have demonstrated between ΔMI and both fMRI and genetic data on MDD are specific to depression or whether they are representative of a trans-diagnostic association between ΔMI and functional dysconnectivity and/or risk genes for mental health disorders more generally. As a first step in addressing this question, we tested for spatial colocation of the ΔMI map and a map of functional dysconnectivity derived from a prior case-control fMRI study of schizophrenia. We found no significant association, indicating that the abnormalities of FC associated with adult schizophrenia do not coincide anatomically with the cortico-subcortical network that demonstrated sex differences in adolescent development. In a second step, we tested for enrichment by schizophrenia-associated genes of the list of genes that were identified by PLS analysis as transcriptionally colocated with the ΔMI map. We found no evidence for significant enrichment of this gene list by risk genes for schizophrenia. In summary, these two specificity analyses indicated that the brain systems demonstrating sexually divergent development in adolescence were not anatomically or genetically linked to schizophrenia, suggesting that this normative neurodevelopmental process may be specifically relevant to depression. However, we note that we have only tested for a relationship between ΔMI and two mental health disorders (MDD and schizophrenia). It will be important in the future to explore this relationship across a wider range of disorders to characterize its diagnostic specificity more comprehensively and conclusively. It is conceivable that sex differences in development of this system could be relevant to sex differences in risk for other mental health disorders.

### Methodological limitations

It is a strength of the study that our analysis of sexually divergent brain network development is based on a large, accelerated longitudinal fMRI dataset with approximately equal numbers of males and females in each stratum of the adolescent age range. However, previous work has found substantial overlap in male and female distributions of multiple brain measures (*74*, *75*), and the metrics analyzed here (FC_14_, FC_14 − 26_, and ΔMI) are group-level parameters. Thus, all reported sex differences are reflective of a group mean difference, estimated from FC distributions that substantially overlap between the sexes. On this basis, we are not arguing that female and male brains are distinctly dimorphic (*76*). Furthermore, this study included only data on biological sex such that we cannot comment on the effects of gender.

Limitations of the study include our reliance on gene expression maps from postmortem examination of six adult, mostly male, brains. This dataset is used widely and has been invaluable in shedding new light on the molecular correlates of neuroimaging phenotypes (*77*). Biological validation of sexually divergent adolescent development of this cortico-subcortical system derived from fMRI would be more directly informed by sex-specific human brain maps of whole-genome transcription in adolescence, but to the best of our knowledge, these data are not currently available. It will also be important in the future to test the hypothesis that an anatomically homologous cortico-subcortical system has divergent adolescent development in animal models that allow more precise but invasive analysis of the cellular and molecular substrates of fMRI phenotypes than is possible in humans.

Here, we used spin tests to correct for the confounding effects of spatial autocorrelation. Spatial autocorrelation of statistical brain maps can cause inflated estimates of the probability of spatial colocation or correlation between two maps (*78*). The spin-test procedure addresses this issue by conserving the spatial autocorrelational structure of the maps by randomly “spinning” or spherically rotating each map over the surface of the brain and calculating the spatial colocation statistic after each spin permutation (*79*). Other methods for testing spatial colocation in the context of spatial autocorrelation have been proposed, and this remains an active focus for ongoing research, especially in relation to colocation of neuroimaging phenotypes and brain gene transcriptional maps (*78*).

Social and environmental factors are relevant modulators of psychiatric disorders (*80*) and have not been assessed in this study. These factors (i) can be neurodevelopmentally relevant, i.e., childhood socioeconomic status influences the pace of brain development (*81*), and (ii) can help explain sex and gender differences in mental health outcomes, i.e., previous studies have demonstrated a relationship between social inequality and gender disparities in mental health (*82*). This naturally leads to the question of how sexually divergent functional network development might be modulated by socioeconomic deprivation or other environmental risk factors for mental health disorder. We suggest that deeper understanding of these potential interactions between biological programs of sexually divergent brain development on one hand and gendered or generic social stressors in childhood and adolescence on the other hand will be an important strategic goal for the future of mental health science.

## MATERIALS AND METHODS

### Study sample

A total of 520 analyzable fMRI scans were available for *N* = 298 healthy participants, aged 14 to 26 years, each scanned one to three times as part of an accelerated longitudinal study of adolescent brain development [Neuroscience in Psychiatry Network (NSPN); see the “Data” section in Supplementary Text] (*1*, *2*, *6*, *14*, *38*). Participants self-identified their sex as either male or female. There were approximately equal numbers of males and females in each of five age-defined strata at baseline (Table 1). All participants aged 16 years or older gave informed consent; participants younger than 16 gave informed assent, and consent was provided by their parent or guardian. The study was ethically approved by the National Research Ethics Service and conducted in accordance with U.K. National Health Service research governance standards.

### fMRI data acquisition

fMRI data were acquired at three sites, on three identical 3T Siemens MRI scanners (Magnetom TIM Trio, VB17 software version), with a standard 32-channel radio frequency (RF) receive head coil and RF body coil for transmission using a multiecho (ME) echo-planar imaging sequence with the following scanning parameters: repetition time, 2.42 s; GRAPPA with acceleration factor = 2; flip angle = 90°; matrix size = 64 × 64 × 34; field of view = 240 mm by 240 mm; in-plane resolution = 3.75 mm by 3.75 mm; slice thickness = 3.75 mm with 10% gap, sequential slice acquisition, 34 oblique slices; bandwidth, 2368 Hz/pixel; and echo times (TE) = 13, 30.55, and 48.1 ms.

### fMRI data preprocessing

fMRI data were preprocessed using multi-echo independent component analysis (ME-ICA) (*83*, *84*), which identifies and removes sources of variance in the time series that do not scale linearly with TE and are therefore not representative of blood oxygenation level–dependent contrast. Ventricular time series, representing variance in cerebrospinal fluid, were regressed from parenchymal time series using Analysis of Functional NeuroImages (AFNI) (*85*). Scans were parcellated into 360 bilateral cortical regions using the Human Connectome Project (*86*) template, and 16 bilateral subcortical regions (amygdala, caudate, diencephalon, hippocampus, nucleus accumbens, pallidum, putamen, and thalamus) were defined by Freesurfer’s “aseg” parcellation template (*87*, *88*). Regional time series were averaged over all voxels within each parcel and band-pass–filtered by the discrete wavelet transform, corresponding to the frequency range 0.025 to 0.111 Hz (*89*).

After preprocessing and quality control of each individual scan, we retained regional time series for 330 cortical and 16 subcortical nodes. Each regional time series was normalized by subtracting the mean and dividing by the SD. Thirty cortical regions were excluded because of low regional mean signal, defined by a low *Z* score of mean signal intensity in at least one participant (*Z* < −1.96; see fig. S2 for details on retained regions). The FC between each regional pair of normalized fMRI time series was estimated by Pearson’s correlation coefficient (*r*) for each possible pair of regions, resulting in a 346 × 346 symmetric association or FC matrix. The FC *r* values were subsequently transformed to *Z* scores by Fisher’s transformation (*90*), so the units of FC are SDs of the normal distribution. Last, we regressed each pairwise correlation or edge on the time-averaged head motion of each participant (mean FD). The residuals of this regression, i.e., motion-corrected *Z* scores, were the estimates of FC used for further analysis (fig. S4). The marginal means of the rows (or columns) of this motion-corrected, *Z*-transformed connectivity matrix yield the vector of regional or nodal weighted degrees (*13*). Thus, for each region *i*, we calculated the mean weighted degree *k* as follows

$$k_{i}=\frac{1}{N-1}*\sum_{j=1;j\neq i}^{N}w_{i,j}$$ (1)

where *k_i_* is the mean weighted degree of node *i*, *N* is the number of nodes in the network, and *w*_*i*, *j*_ is the weight of the edge between node *i* and an arbitrary node *j*. The sum is taken over all edges *w*_*i*, *j*_(*j* ≠ *i* = 1,2,3, …*N* = 346).

### Estimating parameters of adolescent development and testing sex differences

Previous work on this dataset did not find evidence for nonlinear trajectories of development of FC between the majority of all possible pairs of regional nodes (see the “Nonlinear effects of age” section in Supplementary Text; fig. S45) (*6*). Therefore, we used a linear function to model the fixed effect of age on regional and edgewise metrics of cortico-cortico, subcortico-cortical, and cortico-subcortical FC (fig. S7), also including the fixed effect of site and a subject-specific intercept as a random effect, in LMEs fit separately for males and females (see the “Sex stratified analysis of developmental parameters” section in Supplementary Text; figs. S46 and S47).

Baseline connectivity (FC_14_) was estimated as the predicted FC at age 14, and the adolescent rate of change (FC_14 − 26_) was estimated as linear rate of change of connectivity between 14 and 26 years (Fig. 1). We calculated the MI, as the Spearman’s correlation (ρ) of edgewise FC_14_ and FC_14 − 26_ (Fig. 2). The sign of this correlation defines the two different modes of maturational development during adolescence: disruptive and conservative. In conservative development, that is, in the case of a positive MI, nodes are highly connected at baseline consolidate, that is, they become (even) more strongly connected over the course of adolescence. A disruptive change, that is, a negative MI, can mean that either the respective node was highly connected at baseline and becomes weaker with age or it was weakly connected at baseline and becomes stronger over the course of adolescence.

We parametrically tested for the significance of the sex difference in all developmental parameters using a *Z* test (*91*). In short, the *Z* statistic is estimated as the difference in developmental parameters divided by the SE of the difference in the parameters. More specifically, the *Z* score for sex difference in MI (ΔMI) was estimated by

$$z=\frac{{\text{MI}}_{\text{female}}-{\text{MI}}_{\text{male}}}{SE_{{\text{MI}}_{\text{female}}-{\text{MI}}_{\text{male}}}}=\frac{{\text{MI}}_{\text{female}}-{\text{MI}}_{\text{male}}}{\sqrt{S{E_{\text{female}}}^{2}+S{E_{\text{male}}}^{2}}}$$ (2)

where MI_female_ and MI_male_ are the MI for each sex and SE_female_ and SE_male_ are the SEs of MI for each sex.

For FC_14_ and FC_14 − 26_, the SE of the sex difference in parameters is defined differently. The *Z* score for the sex difference in FC_14_ (ΔFC_14_), for example, is estimated by

$$Z=\frac{F{C_{14}}_{\text{female}}-F{C_{14}}_{\text{male}}}{SE_{F{C_{14}}_{\text{female}}-F{C_{14}}_{\text{male}}}}=\frac{F{C_{14}}_{\text{female}}-F{C_{14}}_{\text{male}}}{\sqrt{{\left(\frac{SE_{\text{female}}}{N_{\text{female}}}+\frac{SE_{\text{female}}}{N_{\text{male}}}\right)}^{2}}}$$ (3)

where FC_14female_ and FC_14male_ are the baseline connectivity at age 14 for females and males, respectively, SE_female_ and SE_male_ are the SEs of FC_14_ for each sex, and *N*_female_ and *N*_male_ are the numbers of females and males. The same estimator of *Z* scores (Eq. 3) was also specified for analysis of between-sex differences in FC_14 − 26_. The difference between estimators (Eqs. 2 and 3) of the sex differences in these developmental parameters results from the fact that the SE of MI is the SE of the correlation between FC_14_ and FC_14 − 26_, whereas the SEs of FC_14_ and FC_14 − 26_ are the SEs of the regression coefficients, which include the number of observations in the denominators.

Thus, the *Z* score for sex difference in three developmental fMRI parameters was estimated at each of 346 cortical and subcortical regions. We tested for statistical significance using *P* values from the standard normal distribution controlled for multiple comparisons by the false discovery rate (FDR). For each whole brain map, comprising 346 regional *P* values, we used FDR = 5% as the threshold for significant sex difference in developmental fMRI parameters.

Each analysis of spatial colocation of two cortical maps is reported with both the parametric *P* value corresponding to the Pearson correlation (**r**), as well as a *P* value derived from the more conservative spin-test permutation (*4*, *79*), which conserves the spatial autocorrelation of both maps (**P**_**spin**_). Briefly, *P* values for the spatial correlation between two maps were estimated by comparing the magnitude of the empirical correlation coefficient between the two maps to a null distribution of correlations based on a set of 10,000 random spatial permutations. The permutation was applied in both directions (i.e., by permuting both maps, before comparing each permuted map to the empirical version of the other map) before calculating the average *P* value (see the “Spatial autocorrelation” section in Supplementary Text) (*4*).

### Enrichment analysis

We extracted the first component (PLS1) of a PLS regression of ΔMI on postmortem gene expression data collected from six donor brains (five males) (*49*) and provided by the Allen Human Brain Atlas (Fig. 4). We controlled the PLS regression for spatial autocorrelation using spin tests (see the “Spatial permutation ‘spin’ testing” section in Supplementary Text for details). We first tested for developmental gene enrichment using the CSEA tool (*54*). We then used a median rank–based approach to assess the enrichment of PLS1 on several published gene lists (*92*). This approach estimates the degree to which the ranked list of genes identified by PLS is enriched for a prior reference set of genes (e.g., MDD risk genes from GWAS) by comparing median rank of the prior reference set of genes observed in the PLS1 list to a null distribution of the median rank of a randomly sampled set of genes matched to the prior reference set in terms of number of genes and gene length (see the “Enrichment analysis” section in Supplementary Text for details). Thus, if the prior reference gene set has a real median rank that is significantly lower than the median rank of comparable random sets of genes, then the reference gene set is enriched in the bottom of the PLS1 list, whereas if the median rank of the reference gene set is significantly higher than the rank of the random gene sets, then the reference gene set is enriched in the top end of the PLS1 list. In this way, we tested for gene enrichment for prenatal (*56*) and adult (*55*) cell types and MDD-related genes (*57*).

### fMRI connectivity in major depressive disorder

We constructed a MDD case-control map by conducting multiple *t* tests for the difference in nodal weighted degree of FC between two groups of resting state fMRI data from an independent sample (*39*, *40*) of 46 healthy controls and 50 MDD cases (see the “Colocation with depression” section in Supplementary Text and table S8). We then correlated the ΔMI map with the MDD case-control *t* map and tested for significant colocation using the spin-test procedure to control for spatial autocorrelation in both maps (Fig. 3).

### Diagnostic specificity

Using identical methods to those described in detail for MDD case-control analysis of abnormalities in resting state fMRI connectivity, we constructed a map of schizophrenia case-control differences in FC from a prior study of 81 healthy controls and 67 schizophrenia cases (see the “Diagnostic specificity” section in Supplementary Text) (*41*, *42*). We then correlated the ΔMI map with the schizophrenia case-control *t* map and tested for significant anatomical colocation using the spin-test procedure to control for spatial smoothness of both maps. Using the same methods as described in detail for MDD risk gene enrichment analysis, we also tested the list of genes transcriptionally colocated with ΔMI (PLS1) for enrichment by a set of 130 schizophrenia-related risk genes based on the largest available GWAS of schizophrenia (*43*).

## Supplementary Materials

### This PDF file includes:

Supplementary Text Figs. S1 to S47 Tables S1 to S9 References

## Notes

View/request a protocol for this paper from *Bio-protocol*.
