# Supplementary material for: Sexually divergent development of depression-related brain networks during healthy human adolescence
Source: Sci Adv. 2022 May 27;8(21):eabm7825. doi: 10.1126/sciadv.abm7825 (PMC9140984; doi:10.1126/sciadv.abm7825)
Supplement: Supplementary file 1 — Supplementary Text Figs. S1 to S47 Tables S1 to S9 References [file sciadv.abm7825_sm.pdf]

Science Advances
AAAS

Supplementary Materials for

Sexually divergent development of depression-related brain networks during healthy human adolescence

Lena Dorfschmidt *et al.*

Corresponding author: Lena Dorfschmidt, ld548@cam.ac.uk

Sci. Adv. **8**, eabm7825 (2022)
DOI: 10.1126/sciadv.abm7825

This PDF file includes:

Supplementary Text
Figs. S1 to S47
Tables S1 to S9
References

Science Advances

AAAS

Supplementary Materials for

Sexually divergent development of depression-related brain networks during healthy human adolescence

Lena Dorfschmidt*, Richard A. Bethlehem, Jakob Seidlitz, František Váša, Simon R. White, Rafael Romero-García, Manfred G. Kitzbichler, Athina R. Aruldass, Sarah E. Morgan, Ian M. Goodyer, Peter Fonagy, Peter B. Jones, Ray J. Dolan, the NSPN consortium, Neil A. Harrison, Petra E. Vértes, Edward T. Bullmore

*Corresponding author. Email: ld548@cam.ac.uk

This PDF file includes:

Supplementary Text
Figs. S1 to S47
Tables S1 to S9

Table of Contents

Supplementary Text **6**
Data **6**
Data Collection 6
MRI Sample 6
MRI Data Acquisition 6
MRI Data Preprocessing 7
Exclusion Criteria 8
Analysis of Sex Effects on Parameters of Adolescent Brain Development **9**
Non-Linear Effects of Age 9
Sex Stratified Analysis of Developmental Parameters 9
Age x Sex Effects on Maturational Index 11
Spatial Permutation “spin” Testing: 13
Enrichment Analysis **14**
Partial Least Squares Regression 14
Median Rank-Based Gene Enrichment 14
Matching for Gene Length 15
Developmental Enrichment 15
Prenatal Cell Type Enrichment 15
Adult Cell Type Enrichment 16
Co-location with Depression **17**
Sample Overview 17
MRI Preprocessing 17
Case-Control Map 18
Sensitivity Analyses **19**
Motion-Matched Sample 19
Global Signal Regression Sample 19
FD regression by sex sample 19
Effects of Intracranial Volume 19
Effects of global functional connectivity 21
Diagnostic Specificity **23**
Sample Overview 23
MRI Preprocessing 23
Case-Control Map 24
NSPN Consortium Author List **25**
NIMA Consortium Author List **26**

Supplementary Figures 29
Rationale 29
Fig. S1. Maturational Index Estimation: 29
Data 30
Fig. S2. Regional nodes included after preprocessing and quality control: 30
Head Motion 31
Fig. S3. Sex differences in framewise displacement: 31
Fig. S4. Effect of head motion (FD) on functional connectivity (FC) in the original sample: 32
Analysis of Effects on Parameters of Adolescent Brain Development 33
Fig. S5. Functional connectivity estimation: 33
Fig. S6. Additional Subcortical Plots: 34
Fig. S7. All baseline connectivity $\mathrm{FC}14$ plots: 35
Fig. S8. Significance of sex difference in baseline connectivity $\mathrm{FC}14$: 36
Fig. S9. All adolescent rate of change $\mathrm{FC}14-26$ plots: 37
Fig. S10. Significance of sex difference in adolescent rate of change $\Delta\mathrm{FC}14-26$: 38
Fig. S11 All maturational index plots: 39
Fig. S12. Significance of Sex Difference in MI: 40
Fig. S13. Trends in disruptive and conservative development of connectivity: 41
Fig. S14. Trends in negative $\Delta\mathrm{MI}$: 42
Fig. S15. Unimodal-Transmodal Axis was correlated with MI and $\Delta\mathrm{MI}$: 43
Fig. S16. Positive $\Delta\mathrm{MI}$: 44
Enrichment Analysis 45
Fig. S17. Chromosomal null models: 45
Fig. S18. Cell type enrichment: 46
Co-Location with Depression 47
Fig. S19. Specificity of Anatomical Co-Location with Depression 47
Sensitivity Analyses 48
Fig. S20. Effect of head motion (FD) on functional connectivity (FC) in the FD regression by sex sample: 48
Fig. S21. Replication of key elements of Fig. 1 in sample where FD was regressed per sex group: 49
Fig. S22. Replication of key elements of Fig. 2 in sample where FD was regressed per sex group: 50
Fig. S23. Trends in the sex difference in maturational development (ΔMI) in the sample where FD was regressed per sex group: 51
Fig. S24. Replication of key elements of Figs. 3 and 4 in the sample where FD was regressed per sex group: 52
Fig. S25. Replication of key elements of Fig. 1 in the GSR sample: 54
Fig. S26. Replication of key elements of Fig. 2 in the GSR sample: 55
Fig. S27. Trends in the sex difference in maturational development (ΔMI) in the GSR sample: 56
Fig. S28. Replication of key elements of Figs. 3 and 4 in the GSR sample: 57
Fig. S29. Effect of head motion (FD) on functional connectivity (FC) in the motion-matched sample: 58
Fig. S30. Replication of key elements of Fig. 1 in the motion-matched sample: 59

Fig. S31. Replication of key elements of Fig. 2 in the motion-matched sample: 60
Fig. S32. Trends in the sex difference in maturational development (ΔMI) in the motion-matched sample: 61
Fig. S33. Replication of key elements of Figs. 3 and 4 in the motion-matched sample: 62
Fig. S34. Replication of key elements of Fig. 1 in edge-wise ICV-corrected sensitivity analysis: 63
Fig. S35. Replication of key elements of Fig. 2 in edge-wise ICV-corrected sensitivity analysis: 64
Fig. S36. Trends in the sex difference in maturational development (ΔMI) in the ICV-corrected sample: 65
Fig. S37. Replication of key elements of Figs. 3 and 4 in edge-wise ICV-corrected sensitivity analysis: 66
Fig. S38. Replication of key elements of Fig. 1 in global FC-corrected sensitivity analysis: 67
Fig. S39. Replication of key elements of Fig. 2 in global FC-corrected sensitivity analysis: 68
Fig. S40. Trends in the sex difference in maturational development (ΔMI) in global FC-corrected sensitivity analysis: 69
Fig. S41. Replication of key elements of Figs. 3 and 4 in global FC-corrected sensitivity analysis: 70
Fig. S42. Sex Difference in Maturational Index in Motion Sensitivity Analyses: 72
Diagnostic Specificity **73**
Fig. S43. Specificity analysis of the relationships between ΔMI, functional dysconnectivity in schizophrenia, and brain expression of risk genes for schizophrenia. 73
Contextualization **74**
Fig. S44. Illustrative correlations between ΔMI and three genes (*SST*, *NPY* and *CORT*): 74
Methodological Considerations **75**
Fig. S45. Non-linear Effects of Age on FC: 75
Fig. S46. Random effect variance: 76
Fig. S47. Age x Sex Interaction Model: 77

Tables 78
Data 78
Table S1 NSPN sample overview: 78
Analysis of Effects on Parameters of Adolescent Brain Development 79
Table S2. Age Effects on FC per Sex 79
Table S3. ROIs with significantly different ΔMI: 80
Enrichment Analysis 87
Table S4. Chromosomal Enrichment Null Model Statistics: 87
Table S5. Prenatal Enrichment Null Model Statistics: 89
Table S6. Adult Cell Enrichment Null Model Statistics: 90
Table S7. MDD Enrichment Null Model Statistics: 92
Co-Location with Depression 93
Table S8. BioDep Sample Overview: 93
Sensitivity Analyses 94
Table S9. NSPN Motion-Matched Sample Overview: 94

Supplementary Text

Data

Data Collection

The data was collected as part of the Neuroscience in Psychiatry Network (NSPN), a joint initiative by the University of Cambridge and the University College London, with the aim of collecting a general population sample in an accelerated longitudinal design to measure developmental change in the population of Greater London and Cambridgeshire, broadly representative of the populations of England and Wales. The final sample consisted of 2402 subjects, aged 14 to 26, that underwent a self-assessment of well-being and demographics, with a subset undergoing functional and structural imaging procedures as detailed below.

All participants aged 16 and older provided informed written consent for each aspect of the study, and parental consent was obtained for those aged 14–15 years. The study was ethically approved by the National Research Ethics Service and was conducted in accordance with NHS research governance standards.

MRI Sample

A subsample of 306 adolescents was invited to undergo functional and structural neuroimaging assessments. The exclusion criteria for this sample included: a current or past history of neurological disorder or learning disability, and current treatment for psychiatric disorder or drug or alcohol dependence. Each participant in the scanning sample was invited to provide MRI data on at least two occasions; at baseline and at follow-up 12-18 months later, with 29 participants additionally invited to attend follow-up scanning six months after baseline. The fMRI scan was the first in a series of scans collected in each scanning session, which also included structural MRI using the multi-parameter mapping (MPM) sequence (*93*), and diffusion weighted imaging. A total of 556 functional scans were available after repeated scanning and quality control of this cohort.

MRI Data Acquisition

Functional MRI data were acquired at three sites, on three identical 3T Siemens MRI scanners (Magnetom TIM Trio, VB17 software version), with a standard 32-channel radio-frequency (RF) receive head coil and RF body coil for transmission using a multi-echo echo-planar (EPI) imaging sequence (*94*) with the following scanning parameters: repetition time (TR): 2.42s; GRAPPA with acceleration factor 2; flip angle: 90; matrix size: 64x64x34; FOV: 240x240mm; in-plane resolution: 3.75x3.75 mm; slice thickness: 3.75 mm with 10% gap, sequential slice acquisition, 34 oblique slices; bandwidth 2368 Hz/pixel; echo times (TE) 13, 30.55 and 48.1 ms; total scan time 11 minutes.

MRI Data Preprocessing

Freesurfer v5.3.0 was used to process individual structural scans with a pipeline compromising skull-stripping, segmentation of cortical grey and white matter and reconstruction of the cortical surface and grey-white matter boundary (*95*). Subsequently, all scans were stringently quality controlled by re-running the reconstruction algorithm after the addition of control points and white matter edits as previously described (*6*, *14*).

AFNI was used for basic preprocessing of functional MRI scans as follows: All volumes acquired during steady-state equilibration (15 s) were discarded. Motion correction parameters and parameters for anatomical-functional coregistration were calculated from the images acquired with TE = 30.55 ms. The first volume after equilibration was used as the base EPI image. Matrices for de-obliquing and six-parameter rigid body motion correction were computed. Then, 12-parameter affine anatomical-functional coregistration was computed using the LPC cost function (*96*), with the EPI base image as the LPC weight mask. Matrices for de-obliquing, motion correction, and anatomical-functional coregistration were combined into a single alignment matrix using the concatenation approach from the AFNI tool *alignepianat.py*. The images for each TE were then slice-time corrected and spatially aligned through application of the alignment matrix. The coregistration of structural and functional scans was visually assessed.

Functional MRI data were preprocessed using multi-echo independent component analysis (ME-ICA; (*83*, *84*)) which identifies and removes sources of variance in the times series that do not scale linearly with TE and are therefore not representative of the BOLD signal. The retained independent components, representing BOLD contrast, were optimally recomposed to generate a broadband denoised fMRI time series at each voxel. Regional time series were averaged over all voxels within each parcel and bandpass filtered by the discrete wavelet transform, corresponding to a frequency range of 0.025-0.111 Hz (*89*).

An overall estimate of head motion by each participant, mean framewise displacement (FD), was calculated from the six motion parameter time series (three rotation and three translation parameters) estimated during scan re-alignment. More specifically, the framewise displacement at timepoint t was calculated as:

$$FD_t = \sum_{d} |d_{t-1} * d_t| + 50 * \frac{\pi}{180} * \sum_{d} |\omega_{t-1} * \omega_t| \qquad (1)$$

where d denote the translation distances {x,y,z}, ω denotes the rotation angles $\{\alpha, \beta, \gamma\}$.

Scans were parcellated into 360 bilateral cortical regions using the Human Connectome Project (HCP; (*86*)) template and 16 bilateral sub-cortical regions (amygdala, caudate, diencephalon, hippocampus, nucleus accumbens, pallidum, putamen, and thalamus) defined by Freesurfer's *aseg* parcellation (*87*). After within-subject preprocessing and quality control, we retained regional time

series for 330 cortical and 16 subcortical nodes. 30 cortical regions were excluded due to low regional mean signal ($Z < -1.96$); see SI Fig. S2 for a map of retained regions. Individual functional connectivity matrices were estimated by Pearson's correlation for each possible of pair of nodes. Finally, we regressed each pairwise correlation or edge on the time-averaged head motion of each participant (mean FD). The residuals of this regression were the estimates of functional connectivity used for further analysis.

Exclusion Criteria

A total of 36 scans were excluded. 17 scans were excluded due to high in-scanner motion (defined as mean FD > 0.3 mm or maximum FD > 1.3 mm), 9 due to coregistration errors, 7 due to a lack of convergence of the ME-ICA algorithm, 2 due to parcellation errors, and 1 due to extensive signal dropout (as defined above).

Analysis of Sex Effects on Parameters of Adolescent Brain Development

Non-Linear Effects of Age

It is conceivable that there could be non-linear effects of age on functional connectivity. However, in previous work on the same data set (*6*), we investigated potential non-linear effects of age and found no substantial evidence for non-linearity in these data. Specifically, Váša et al. (2020), fitted smoothing splines (generalized additive mixed models) to edge-wise trajectories of functional connectivity development, using the "gamm" function in R, with the effect of age modelled as the weighted sum of 10 cubic b-splines with knots placed at quantiles of the data and smoothing optimized using restricted maximum likelihood (*97*). This modeling strategy is adaptive to the non-linearity of age effects, such that non-linear trajectories will be best fitted by spline functions with degrees of freedom greater than 2, whereas linear trajectories will be best fitted by spline functions with 2 df, analogous to the intercept and gradient parameters of a simple linear model. We found that approximately 70% of all edges had linear trajectories that were best fit by spline functions with 2 df (Supplementary Figure S8, (*6*)). Moreover, there was no evidence from this analysis that the minority of edges with non-linear trajectories were concentrated on anatomically specific brain regions or systems. For these reasons, we adopted a linear function for age-related effects on functional connectivity in our modeling of these data. However, to mitigate any residual concerns that non-linearity of age-related changes in functional connectivity might confound sex differences in MI, we examined the relationship between ΔMI and the nodal mean degrees of freedom for spline functions of age. As shown in Figure S45 there was no significant correlation ($r = 0.006, P = 0.9$). This indicates that there is no evidence for a relationship between non-linear trajectories of functional connectivity development and sex differences in maturational index.

Sex Stratified Analysis of Developmental Parameters

For the principal analyses reported in this paper, we used a sex-stratified approach to analyse (sex effects on) parameters of adolescent brain development. This means we fit a linear mixed effects (LME) model to estimate FC_{14} and FC_{14-26} separately for each sex, as detailed below.

For each sex, we predicted functional connectivity at edge level using linear mixed effects models. These models included age as the main fixed effect of interest and scanner site as a fixed effect covariate, as well as a subject-specific intercept as a random effect, as follows:

$$FC_{edge} \sim 1 + \beta_{age} * age + \beta_{site} * site + \gamma_{subject} * (1|subject) + \epsilon \qquad (2)$$

where FC refers to the functional connectivity at edge level, β refers to coefficients for the fixed effects, $\gamma_{subject}$ refers to the coefficients for random effects, and ϵ represents the residual error.

We then derived baseline connectivity at age 14 from Equation 2 as:

$$FC = 1 + \beta_{age} * 14 + \beta_{site_2} * (1/3) + \beta_{site_3} * (1/3) \quad (3)$$

Whereas the adolescent rate of change is simply the β coefficient of age from (2) as:

$$FC_{14-26} = \beta_{age} \quad (4)$$

We then combined the sex specific estimates of these two developmental parameters to estimate a sex-specific estimate of MI. Thus, in each sex we evaluated at each node the linear relationship (using Spearman's ρ) between the ranked ($R(\ldots)$) edge-wise parameters of baseline connectivity ($R(FC_{14}$)) and the adolescent rate of change ($R(FC_{14}$) for each sex, e.g.:

$$MI = \frac{cov(R(FC_{14}), \ R(FC_{14-26}))}{\sigma_{R(FC_{14})}, \sigma_{R(FC_{14-26})}} \quad (5)$$

Where $\sigma_{R(FC_{14})}$ and $\sigma_{R(FC_{14-26})}$are the standard deviations of the ranked variables.

Finally, between-sex differences were estimated by subtracting each male-specific parameter from the corresponding female-specific parameter:

$$\Delta FC_{14} = FC_{14_{female}} - FC_{14_{male}} \quad (6)$$

$$\Delta FC_{14-26} = FC_{14-26_{female}} - FC_{14-26_{male}} \tag{7}$$

$$\Delta MI = MI_{female} - MI_{male} \tag{8}$$

Our principal reason for choosing a sex stratified approach was that it allows the variance of the random effects estimated in Equation 2, Var($\gamma_{subject}$), to differ between sexes. As shown in Fig. S46 the distributions of random effects were indeed not identical in males and females – males were more variable. Higher variance of random effects was negatively correlated with lower residual variance (denoted ϵ in Equation 2) in both sexes; but the strength of correlation was greater in females than males, as shown in Fig S46B. Thus, although the between-sex difference in random effects variance was not statistically significant by a permutation test (Fig S46C) there was a degree of difference which would influence the residual variance and therefore the significance of the standardised developmental parameters. It is for this reason that we principally used the sex-stratified approach to linear mixed effects modeling of these longitudinal data.

Age x Sex Effects on Maturational Index

To demonstrate robustness of our results to an alternative modeling strategy, we also analysed all the data (male and female combined) using a linear mixed effects model to estimate the main effects of age and sex, and the age-by-sex interaction effect, on functional connectivity (FC) at each edge:

$$FC_{edge} \sim 1 + \beta_{age} * age + \beta_{sex} * sex + \beta_{age*sex} * age * sex + \beta_{site} * site + \gamma_{subject} * (1|subject) + \epsilon \tag{9}$$

where FC refers to the functional connectivity at edge level, β refers to coefficients for the fixed effects, $\gamma_{subject}$ refers to coefficients for random effects and ϵ represents the residual error.

On this basis, we can then estimate FC_{14} for males and females as follows:

$$FC_{14_{female}} = 1 + \beta_{age} * 14 + \beta_{sex} * 0 + \beta_{age*sex} * 0 * 14 + \beta_{site_2} * (1/3) + \beta_{site_3} * (1/3) \tag{10}$$

$$FC_{14_{male}} = 1 + \beta_{age} * 14 + \beta_{sex} * 1 + \beta_{age*sex} * 1 * 14 + \beta_{site_2} * (1/3) + \beta_{site_3} * (1/3) \tag{11}$$

And likewise we can estimate FC_{14-26} for males and females:

$$FC_{14-26_{female}} = \beta_{age} + \beta_{age*sex} * 0 \tag{12}$$

$$FC_{14-26_{male}} = \beta_{age} + \beta_{age*sex} * 1 \tag{13}$$

Finally, these sex-specific estimates of FC_{14} and FC_{14-26} can be combined to estimate sex-specific estimates of MI (by Spearman correlating the edgewise parameters at each node as described above) and the between-sex difference in MI, ΔMI, as:

$$\Delta MI = MI_{female} - MI_{male} \tag{14}$$

As in our principal analysis, we tested the significance of the sex difference in MI by a parametric approach, comparing the slopes of the regression of FC_{14} and FC_{14-26} (*91*).

We found that the correlation between our principal sex-stratified results and the results of this alternative analysis, based on a fixed term for the sex-by-age interaction, were nearly identical, with parameters estimated by sex-stratified and sex-by-age interaction models demonstrating a high degree of correlation ($r > 0.9$; SI Fig. S47). Thus, we conclude that our principal results from sex-stratified modelling are robust to an alternative modelling strategy that explicitly includes a fixed term for sex-by-age interaction. However, we continue to prefer the sex-stratified approach because of its greater adaptivity to the evident between-sex differences in random effects variance (SI Fig. S46).

Spatial Permutation "spin" Testing:

Spatial auto-correlation amongst neighboring brain regions leads to non-independence of regions which can inflate estimates of the *P*-values for spatial correlation between two (auto-correlated) brain maps. Alexander-Bloch et al. (*79*) first suggested a vertex-wise approach to correct for spatial auto-correlation. Here, we correct all spatial correlations between two cortical maps for spatial auto-correlation using a regional adaptation of the spherical permutation test implemented in Váša et al. (*4*) using this published code: https://github.com/frantisekvasa/rotate_parcellation. Since the publication by Váša et al. (*4*), the effect of parcel centroid definition on spatial permutation procedures has been further researched (*98*). It has been suggested that the previously used method of estimating the parcel centroid by averaging across all vertex coordinates leads to the centroids lying under the surface of the cortical mesh (*98*). Here, we thus define the centroids by computing the parcelwise vertex-by-vertex geodesic distance and assigning the vertex with the smallest average geodesic distance to all other vertices in the parcel as the centroid (*98*).

Briefly, this implementation of the spatial permutation test generated random permutation matrices by (i) generating 3 × 3 matrices with coefficients following independent standard normal distributions, (ii) applying a QR decomposition, and (iii) keeping the orthogonal matrices Q which are uniformly distributed. Subsequently the 'rotated' regions are matched to the coordinates of the original regions using Euclidean distance. Starting at the rotated region with the highest average Euclidean distance to all unrotated regions, the algorithm proceeds in descending order to match each rotated region to the unrotated centroid it is closest to.

For each spatial correlation between two cortical maps in this manuscript, we thus estimate a spin test *P-value* by comparing the magnitude of the empirical correlation coefficient to a null distribution of correlations, based on a set of 10,000 random spatial permutations. The permutation was applied in both directions (i.e. by permuting both maps, before comparing each permuted map to the empirical version of the other map) before calculating the average *P*-value.

We also used spherical permutation testing to assess the significance of the PLS analysis. In this case, we used partial least squares regression to find the cortical map of weighted gene expression (PLS1) that was most strongly co-located with the neuroimaging phenotype represented by the map of sex differences in adolescent brain maturation (ΔMI). The significance of the PLS components was estimated by comparing the empirical variance explained by each component to a null distribution. This null distribution was generated by permuting the order of regions in the outcome variable (Y). To correct this test for spatial auto-correlation, we permute Y using a spherical rotation of regions as described above.

Enrichment Analysis

Partial Least Squares Regression

Partial least squares (PLS) regression has been used as a powerful tool to describe the relationship between two sets of variables (represented as two matrices) which uses latent variables to model the covariance structure between the two. Its ability to handle situations with a large number of potentially multicollinear predictors has made it useful for analysis of neuroimaging data (*5*, *14*, *48*). Briefly, PLS finds components that explain the maximum covariance between the dependent and independent variables. Here, we use PLS as a means to find the weighted gene expression pattern that is most strongly correlated with the anatomical pattern of sex differences in adolescent functional connectivity maturation, ΔMI. Thus we regressed the vector of ΔMI on the 360 by 15,746 matrix of post mortem transcriptomic gene expression data from the Allen Human Brain Atlas collected from 6 donor brains (5 males) (*49*, *99*). We analysed whether the first PLS component explains more variance than expected by chance by randomly permuting the rows of the gene expression matrix and comparing the variance explained by PLS regression of ΔMI on the observed transcriptional data with the distribution of variance in ΔMI explained by 1000 random permutations of the brain gene expression matrix. For the first PLS component (PLS1), which accounted for the greatest proportion of variance, we estimated the variability of each transcript's weighting coefficient by bootstrap resampling (10,000 times) of the brain regional transcription matrix. The effect size and statistical significance of individual transcript weights on PLS1 were defined by the Z-score (observed coefficient divided by bootstrap standard error).

Median Rank-Based Gene Enrichment

We used a median rank-based approach to assess the enrichment of PLS1 on several published gene lists (*55–57*). This allows us to assess whether a given gene list is non-randomly represented among the most strongly weighted PLS1 genes that have brain expression anatomically co-located with the spatial pattern of sex differences in maturational index. To do this, each gene on the prior gene list of interest is ranked in terms of its *Z*-score weighting on PLS1 and the observed median rank is estimated; then an equivalent number of genes, matched for gene length, are randomly selected and their median rank on the PLS1 component is estimated. This second step of randomly selecting and ranking genes is repeated 10,000 times to sample the permutation distribution of median rank. Finally, the null hypothesis that the observed median rank (for the gene list of interest) was not significantly different from the median rank of a random list of genes (matched for gene length and number of genes) was tested by comparing the observed median rank to the centiles of the permutation distribution. For example, for a two-tailed test of significant enrichment with $P < 0.05$, if the observed median rank was lower than the 2.5th percentile of the permutation distribution, then the gene list of interest was significantly enriched among the most negatively weighted PLS1 genes; whereas if the observed median rank was greater than the 97.5th percentile of the permutation distribution then the gene list of interest was significantly enriched among the most positively weighted PLS1 genes.

Finally, statistical significance of observed gene set median ranks was established by comparison with the null median rank distributions from 10,000 gene rank permutations. The direction of the

effect is relative to the median rank expected by chance. Thus, if a gene set's real median rank is significantly lower than expected by chance, the gene set is associated with, or enriched for, the bottom of the PLS1 list since that set of genes ranks lower on PLS1 than expected for a random set of genes of similar length; if it is higher, its genes are enriched towards the top of the list of ranked gene weights on the first PLS component.

Matching for Gene Length

We matched the genes in our empirical gene list for gene length by finding a set of genes in the list of AHBA genes which were close in length by some criterion detailed further below. We then proceeded to resample from that subset of AHBA genes to find a set of genes matched for gene length with the empirical gene lists ($P < 0.05$). We used one of three approaches, in increasing order of strictness, to find this initial set of genes to resample from. (i) In the 'standard' approach, we matched for gene length by finding the 5 nearest neighbours (Mahalanobis distance) of any given gene from an external gene set in the list of AHBA (PLS1) genes. (ii) Should the algorithm fail to find a set of genes matched for gene length in that way ($P < 0.05$), we restricted the approach to find only the 2 nearest neighbours in the same way ('2 nearest neighbours' approach). (iii) Lastly, should that approach fail too, we resampled the 3 nearest neighbours without replacement ('without replacement' approach).

Developmental Enrichment

We uploaded a ranked list of genes with a significantly ($P_{FDR} < 0.05$) negative or positive PLS1 weight respectively to the cell specific enrichment analysis (CSEA) tool (*54*) under the category C*SEA across brain regions and development*. The CSEA tool uses human data from BrainSpan, an atlas of the developing human brain (*100*), with postmortem human brain specimens collected across 13 developmental stages (4 weeks post conception to 60 years of age) in 8-16 brain structures.

Prenatal Cell Type Enrichment

We tested PLS1 for cell type specific enrichment using single-cell transcriptomic gene expression data from mid gestation (gestation week 17 to 18; (*56*)). This data included 16 unique clusters: endothelial cells (End), excitatory deep layer 1 (ExDp1), excitatory deep layer 2 (ExDp2), maturing excitatory (ExM), newborn excitatory neurons (ExN), intermediate progenitor cells (IP), microglia (Mic), oligodendrocyte precursor cells (OPC), outer radial glia (oRG), pericytes (Per), cycling progenitor G2/M phase (PgG2M), cycling progenitor S phase (PgS), ventricular radial glia (vRG).

Adult Cell Type Enrichment

We tested PLS1 for cell type specific enrichment. We used gene expression data of 33 distinct cellular clusters (*55*), including cortical excitatory (Ex) and inhibitory (In) neurons, cerebellar granule (Gran) cells and Purkinje (Purk) neurons, as well as non-neuronal cells, including endothelial cells (End), smooth muscle cells or pericytes (Per), astrocytes (Ast), oligodendrocytes (Oli), oligodendrocyte precursor cells (OPCs), and microglia (Mic). We excluded cell types expressed in the cerebellum only.

Co-location with Depression

Sample Overview

The Biomarkers for Depression (BIODEP) study is a case-control study of adult subjects, aged 25-50 years, with and without major depressive disorder (MDD). Data was collected from a total of 129 subjects: 46 healthy controls, and 83 MDD patients, as measured in a Structured Clinical Interview for DSM-V Depressive Disorders (SCID), as well as by a global Hamilton Rating Scale for Depression (HAM-D) score of higher than 13. The MDD group contained cases with CRP < 3 mg/L (N=53), and cases with CRP > 3 mg/L (N=34). Here, we are focusing on the low CRP cases, only. The final sample after quality control contained 46 healthy control and 50 MDD patients (cf. Supplementary Table S8).

MRI Preprocessing

We used a multi-echo (ME) echoplanar imaging (EPI) sequence (*101*) to collect fMRI data under resting state conditions with the following parameters: relaxation time (TR) = 2.57s; echo times ($TE_{1,2,3}$) = 15, 34 and 54ms; acquisition time = 10mins 42.5s = 250 time points in each fMRI time series. MEEPI data were collected as 32 slices at -30 degrees to the AC-PC line, field of view: 240mm, matrix size: 64x64, voxel resolution: 3.75 3.75 4mm.

The first 6 volumes were discarded to ensure scanner equilibrium, and the remaining data were pre-processed using multi-echo independent component analysis (ME-ICA; (*83*, *84*)) to identify sources of variance in the fMRI time series that scaled linearly with TE and could be confidently regarded as BOLD signal. Other non-BOLD sources of variance, such as head movement, that do not scale with TE, were identified by ME-ICA and discarded. The retained independent components, representing BOLD contrast, were optimally recomposed to generate a broadband denoised fMRI time series at each voxel. This was bandpass filtered using the Maximal Overlap Discrete Wavelet Transform ("modwt" using "la8", the Daubechies orthonormal compactly supported wavelet of length L=8), resulting in a BOLD signal oscillating in the frequency range 0.02-0.1Hz (wavelet scales 2 and 3).

Geometric re-alignment was used to estimate 6 motion parameters for each participant (3 translation and 3 rotation parameters), which were used to calculate an overall estimate of motion - framewise displacement (FD; defined as the Euclidean norm of motion and rotation derivatives in mm: $FD^2 = |\overrightarrow{\Delta x}|^2 + |\overrightarrow{\Delta \theta}|^2$). For each participant, mean FD was calculated by averaging the FD time series. A total of 3 scans were excluded due to high in-scanner motion $\langle FD \rangle_{RMS}$ > 0.3mm or max(FD) > 1.3mm and one subject was dropped due to excessively high mean correlation > 0.7.

Each pre-processed fMRI image was regionally parcellated into 360 bilateral cortical regions using the Human Connectome Project (HCP; (*86*)) template and 16 bilateral sub-cortical regions (amygdala, caudate, diencephalon, hippocampus, nucleus accumbens, pallidum, putamen, and thalamus) provided by Freesurfer's 'aseg' parcellation template (*87*, *88*). The regional mean fMRI

time series estimated for each cortical and sub-cortical region using the non-zero mean variant of the AFNI *3dROIstats* command (*85*). Thus, we estimated a 376 regional time series matrix for each participant.

The functional connectivity between each regional pair of fMRI time series was estimated by Pearson's correlation coefficient r for each possible pair of regions, resulting in a 376x376 symmetric association or functional connectivity matrix. The functional connectivity r values were subsequently transformed to Z-scores by Fisher's transformation (*90*). The mean across rows (or columns) of this motion-corrected, Z-transformed connectivity matrix yields the vector of regional or nodal weighted degrees (*13*). Thus, for each region i we calculated the mean weighted degree k as follows:

$$k_i = \frac{1}{N-1} * \sum_{j=1; j \neq i}^{N} w_{i,j} \tag{15}$$

where k_i is the mean weighted degree of node i, N is the number of nodes in the network, and $w_{i,j}$ is the weight of the edge between node i and an arbitrary node j. The sum was taken over all edges $w_{i,j} (j \neq i = 1,2,3, \dots N = 376)$.

Case-Control Map

We constructed a case-control difference map by estimating the effect (t-value) of group (patient vs. control) on region-wise functional connectivity (FC) strength, controlling for sex. We corrected for multiple comparisons (P < 0.05, FDR-corrected).

Sensitivity Analyses

Motion-Matched Sample

We constructed a motion-matched subsample of the NSPN dataset by removing subjects with particularly high and low framewise displacement (FD) values from the original sample, until no significant difference was observed between males and females. If a subject was included in the sample, all of their follow-up scans were included, too. The final sample consisted of 314 subjects (156 females), 124 of which were scanned once, 89 twice and 4 three times (cf. Supplementary Table S9).

Global Signal Regression Sample

We re-preprocessed all data with an alternative pipeline. The first pre-processing steps were the same as in the original sample (cf. Methods). After ME-ICA pre-processing, however, we performed global signal regression (GSR). The global signal was estimated as the average time series of all cortical voxels. We regressed this time series from each region. From here, we proceeded with wavelet filtering using brainwaver v. 1.6 (https://rdrr.io/cran/brainwaver/) and all following steps as in the original sample.

FD regression by sex sample

We found that after our preprocessing pipeline including edgewise FD regression across the whole sample, there was a sex dependence of FC on motion. Therefore, we constructed a sample where we performed the edgewise FD regression per sex group instead.

Effects of Intracranial Volume

To estimate the effects of intracranial volume (ICV) on our main results, we have included ICV in both the analysis of global functional connectivity, as well as in the maturational index calculation. Specifically, we included ICV as follows:

To estimate the effect of ICV on global functional connectivity, we added ICV to our model as a covariate:

$$FC_{global} \sim 1 + age * \beta_{age} + sex * \beta_{sex} + site * \beta_{site} + ICV * \beta_{ICV} + \gamma_{subject} * (1|subject) + \epsilon \quad (16)$$

Likewise, we also included ICV in our analysis of edge wise functional connectivity.

$$FC_{edge} \sim 1 + age * \beta_{age} + site * \beta_{site} + ICV * \beta_{ICV} + \gamma_{subject} * (1|subject) + \epsilon \quad (17)$$

We predicted the mean ICV at age 14 from the model:

$$ICV_{sex} \sim 1 + age * \beta_{age} + site * \beta_{site} + \gamma_{subject} * (1|subject) + \epsilon \quad (18)$$

as:

$$\mu_{ICV_{14sex}} = 1 + 14 * \beta_{age} + 1/3 * \beta_{site_1} + 1/3 * \beta_{site_2} \quad (19)$$

We then carried the effect of ICV over into the calculation of baseline connectivity (FC_{14}), by adding β_{ICV} (from Equation [17]) multiplied by the mean ICV at age 14 for males and females respectively ($\mu_{ICV_{14sex}}$ from Equation [19]). Thus, at each edge, we calculated baseline connectivity at age 14 as:

$$FC_{14} = 1 + 14 * \beta_{age} + 1/3 * \beta_{site_2} + 1/3 * \beta_{site_3} + \mu_{ICV_{14}} * \beta_{ICV} + \gamma_{subject} * (1|subject) + \epsilon \quad (20)$$

where $\mu_{ICV_{14sex}}$ is the mean ICV at age 14 for males and females respectively. As before, the rate of change is calculated as:

$$FC_{14-26} = \beta_{age} \quad (21)$$

And the MI is Spearman's coefficient of the linear relationship between the ICV-corrected FC_{14} and ICV-corrected FC_{14-26}.

We find that our key results replicate well in the sensitivity analysis including ICV. Specifically, original and ICV-corrected baseline connectivity were moderately correlated ($r_{female} = 0.89, P_{female} < 0.001; r_{male} = 0.98, P_{male} < 0.001$; Fig. S34 A,C), whereas the adolescent rate of change was highly correlated ($r_{female} = 0.94, P_{female} < 0.001; r_{male} = 0.92, P_{male} < 0.001$; Fig. S34 B,D).

The ICV-corrected sex difference in maturational index (ΔMI) is qualitatively and quantitatively

similar to the original result from our main analysis ($r = 0.98, P < 0.01$; Fig. S35 A, B). Further, we confirmed that the correlation of ΔMI with the MDD-case-control map is conserved after edge-wise ICV correction ($r = 0.41, P < 0.001$; Fig. S37A). Negative ΔMI regions (blue) show decreased FC in MDD compared to controls. We further ran the gene enrichment analysis in the replication sample. The original and replication PLS1 components are significantly correlated ($r = 0.92, p < 0.001$). Negatively weighted genes were more strongly expressed in regions of negative ΔMI, that is predominantly female > male disruptive regions; whereas positively weighted genes were more strongly expressed in regions with female > male conservative development indicated by positive ΔMI. We showed that we can replicate the gene enrichment for MDD-related genes (Fig. S37G). Further, negatively weighted genes were significantly enriched for genes expressed by adult astrocytes, inhibitory and excitatory neurons and positively weighted genes were enriched for oligodendrocytes (Fig. S37F), as well as X-Chromosome genes (Fig. S37H).

Effects of global functional connectivity

To estimate the effect of global FC we included FC_{global} in our analysis of edge wise functional connectivity as such:

$$FC_{global} \sim 1 + age * \beta_{age} + sex * \beta_{sex} + site * \beta_{site} + \mu_{FC_{global}} * \beta_{FC_{global}} + \gamma_{subject} * (1|subject) + \epsilon \quad (22)$$

We then carried the effect of FC_{global} over into the calculation of baseline connectivity (FC_{14}), by adding $\beta_{FC_{global}}$ (from Equation [22]) multiplied by the mean global FC at age 14 for males and females respectively. Thus, at each edge, we calculated baseline connectivity at age 14 as:

$$FC_{14} = 1 + 14 * \beta_{age} + sex * \beta_{sex} + 1/3 * \beta_{site_1} + 1/3 * \beta_{site_1} + \mu_{FC_{global}} * \beta_{FC_{global}} + \gamma_{subject} * (1|subject) + \epsilon \quad (23)$$

where $\mu_{FC_{global}}$ is the mean global functional connectivity at age 14 for males and females, respectively.

As before, the rate of change is calculated as:

$$FC_{14-26} = \beta_{age} \quad (24)$$

And the MI is the Spearman's coefficient of the correlation between the global FC-corrected FC_{14} and global FC-corrected FC_{14-26}.

We find that our key results replicate well in the sensitivity analysis including global functional connectivity. Specifically, original and global-FC-corrected baseline connectivity are highly correlated ($r_{female} = 0.89, P_{female} < 0.001; r_{male} = 0.98, P_{male} < 0.0011$; Fig. S38A,C), and so is the adolescent rate of change ($r_{female} = 0.94, P_{female} < 0.001; r_{male} = 0.92, P_{male} < 0.001$; Fig. S38B,D).

The global-FC-corrected controlled sex difference in maturational index (ΔMI) is qualitatively and quantitatively similar to the original result from our main analysis ($r = 0.99, P < 0.01$; Fig. S39A,B). Further, we confirmed that the correlation of ΔMI with an MDD-case-control holds in this sample (Fig. S41A) and showed that we can replicate the gene enrichment for MDD-related genes (Fig. S41G), as well as X-Chromosome and neurodevelopmental genes (Fig. S41H).

Diagnostic Specificity

To estimate the specificity of our results, we used identical methods to repeat two key analyses demonstrating relationships between MDD and sexually divergent disruptive development using comparable fMRI and genetic data on schizophrenia

Sample Overview

The COBRE (*41*) study is an open schizophrenia case-control fMRI study of adults, aged 18 to 65 years, in which individuals are identified as either healthy controls or cases of schizophrenia, bipolar or schizoaffective disorder diagnosed using the Structured Clinical Interview for DSM Disorders (SCID; Diagnostic Manual of Mental Disorder, DSM-IV). Exclusion criteria included confirmed or suspected pregnancy, any history of neurological disorders and a history of mental retardation. We downloaded the data from the SchizConnect database (http:/schizconnect.org), where it had been obtained using the Collaborative Informatics and Neuroimaging Suite Data Exchange tool (COINS; http://coins.mrn.org/dx). We excluded 6 cases with a diagnosis of bipolar disorder. After pre-processing and quality control, the analysable fMRI sample included 148 subjects, 81 healthy controls and 67 cases (58 with a diagnosis of schizophrenia and 9 with a diagnosis of schizoaffective disorder).

MRI Preprocessing

Single echo fMRI was collected using an echo-planar imaging (EPI) sequence with the following parameters: 150 volumes, acquisition time = 5 minutes, 32 slices, echo time = 29 ms, repetition time = 2000 ms, voxel size = $3 \times 3 \times 4$ mm^3. A prior pipeline (*44*, *102*) was used to pre-process the fMRI data. This pipeline included: slice acquisition correction, rigid-body head motion correction, co-registration to the T1-weighted image, a standard space transform to the MNI152 template in Talairach space, spatial smoothing, and intensity normalization. We excluded 19 subjects due to excess motion using previously defined criteria. We bandpass filtered the timeseries using wavelet scale 2 (*103*), corresponding to the frequency ranges 0.0625–0.125 Hz.

Each pre-processed fMRI image was regionally parcellated into 360 bilateral cortical regions using the Human Connectome Project (HCP; (*86*)) template and 16 bilateral sub-cortical regions (amygdala, caudate, diencephalon, hippocampus, nucleus accumbens, pallidum, putamen, and thalamus) defined by Freesurfer's *'aseg'* parcellation template (*87*, *88*). 9 regions were excluded due to signal dropout in one or more subjects (missing data or low regional mean signal of $Z <- 1.96$), leaving 367 regions.

The functional connectivity between each regional pair of Z-scored fMRI time series was estimated by Pearson's correlation coefficient r for each possible pair of regions, resulting in a 367x367 symmetric association or functional connectivity matrix. The functional connectivity r values were subsequently transformed to Z-scores by Fisher's transformation (*90*). The mean across rows (or columns) of this motion-corrected, Z-transformed connectivity matrix yields the vector of regional or nodal weighted degrees (*13*). Thus, for each region i we calculated the mean weighted degree k as follows:

$$k_i = \frac{1}{N-1} * \sum_{j=1; j \neq i}^{N} w_{i,j} \tag{25}$$

where k_i is the mean weighted degree of node i, N is the number of nodes in the network, and $w_{i,j}$ is the weight of the edge between node i and an arbitrary node j. The sum was taken over all edges $w_{i,j}(j \neq i = 1,2,3, \ldots N = 367)$.

Case-Control Map

We constructed a case-control difference map by estimating the effect (*t*-value) of group (patient vs. control) on region-wise functional connectivity (FC) strength, controlling for sex, age and mean framewise displacement (FD). This schizophrenia case-control dysconnectivity map was finally tested for significant co-location with the ΔMI map using the spin test procedure to control for spatial auto-correlation in calculation of regional *P*-values and using FDR correction at 5% to control for multiple significance tests over all regions in the brain.

NSPN Consortium Author List

Principal investigators:
Edward Bullmore (CI from 01/01/2017)
Raymond Dolan
Ian Goodyer (CI until 01/01/2017)
Peter Fonagy
Peter Jones

NSPN (funded) staff:
Michael Moutoussis
Tobias Hauser
Sharon Neufeld
Rafael Romero-García
Michelle St Clair
Petra Vértes
Kirstie Whitaker
Becky Inkster
Gita Prabhu
Cinly Ooi
Umar Toseeb
Barry Widmer
Junaid Bhatti
Laura Villis
Ayesha Alrumaithi
Sarah Birt
Aislinn Bowler
Kalia Cleridou
Hina Dadabhoy
Emma Davies
Ashlyn Firkins
Sian Granville
Elizabeth Harding
Alexandra Hopkins
Daniel Isaacs
Janchai King
Danae Kokorikou
Christina Maurice
Cleo McIntosh
Jessica Memarzia
Harriet Mills
Ciara O'Donnell
Sara Pantaleone
Jenny Scott

Affiliated scientists:
Pasco Fearon
John Suckling
Anne-Laura van Harmelen
Rogier Kievit
Richard Bethlehem

NIMA Consortium Author List

Cambridge
Edward T. Bullmore (MD, PI, EC)[1,2,11]
Junaid Bhatti[1]
Samuel J. Chamberlain[1,2]
Marta M. Correia[1,12]
Anna L. Crofts[1]
Amber Dickinson*
Andrew C. Foster*
Manfred G. Kitzbichler[1]
Clare Knight*
Mary-Ellen Lynall[1]
Christina Maurice[1]
Ciara O'Donnell[1]
Linda J. Pointon[1]
Peter St George Hyslop[1,13,14]
Lorinda Turner[31]
Petra Vertes[1]
Barry Widmer[1]
Guy B. Williams[1,14]

Cardiff
B. Paul Morgan (PI)[15]
Claire A. Leckey[15]
Angharad R. Morgan*
Caroline O'Hagan*
Samuel Touchard[15]

Glasgow
Jonathan Cavanagh (PI, EC)[3]
Catherine Deith*
Scott Farmer[16]
John McClean[16]
Alison McColl[3]
Andrew McPherson*
Paul Scouller*
Murray Sutherland[16]

Independent advisor
H.W.G.M. (Erik) Boddeke (EC)[17]

GSK
Jill C. Richardson (EC)[18]
Shahid Khan[11]
Phil Murphy[1]
Christine A. Parker[19]
Jai Patel[11]

Janssen
Declan Jones (EC)[6]
Peter de Boer[4]
John Kemp[4]
Wayne C. Drevets[6]
Jeffrey S. Nye (deceased)
Gayle Wittenberg[6]
John Isaac[6]
Anindya Bhattacharya[6]
Nick Carruthers[6]
Hartmuth Kolb[6]

Kings College London
Carmine M. Pariante (PI)[10]
Federico Turkheimer (PI)[20]
Gareth J. Barker[20]
Heidi Byrom[10]
Diana Cash[20]
Annamaria Cattaneo[10]
Antony Gee[20]
Caitlin Hastings[10]
Nicole Mariani[10]
Anna McLaughlin[10]
Valeria Mondelli[10]
Maria Nettis[10]
Naghmeh Nikkheslat[10]
Karen Randall[20]
Hannah Sheridan*
Camilla Simmons[20]
Nisha Singh[20]
Victoria Van Loo*
Marta Vicente-Rodriguez[20]
Tobias C. Wood[20]
Courtney Worrell*
Zuzanna Zajkowska*

Lundbeck
Niels Plath (EC)[21]
Jan Egebjerg[21]

Hans Eriksson[21]
Francois Gastambide[21]
Karen Husted Adams[21]
Ross Jeggo*
Christian Thomsen[21]
Jan Torleif Pederson[21]
Brian Campbell*
Thomas Möller*
Bob Nelson*
Stevin Zorn*

University of Texas (sub-contracted to Lundbeck)
Jason O'Connor[22]

Oxford
Mary Jane Attenburrow (PI)[7,23]
Alison Baird
Jithen Benjamin[23]
Stuart Clare[25]
Philip Cowen[7]
I-Shu (Dante) Huang[24]
Samuel Hurley*
Helen Jones[23]
Simon Lovestone[7]
(AD, PI, EC) Francisca Mada*
Alejo Nevado-Holgado[7]
Akintayo Oladejo*
Elena Ribe7
Katy Smith[23]
Anviti Vyas*

Pfizer
Zoe Hughes*
Rita Balice-Gordon*
James Duerr*
Justin R. Piro*
Jonathan Sporn*

Southampton
V. Hugh Perry (PI)[27]
Madeleine Cleal*
Gemma Fryatt[27]
Diego Gomez-Nicola[27]
Renzo Mancuso[32]
Richard Reynolds[27]

Sussex
Neil A. Harrison (PI, EC)[28]
Mara Cercignani[28]
Charlotte L. Clarke[28]
Elizabeth Hoskins*
Charmaine Kohn*
Rosemary Murray*
Lauren Wilcock[29]
Dominika Wlazly[30]

University of Toronto (sub-contracted to Cambridge)
Howard Mount[13]

MD = Mood disorder workpackages lead
AD = Alzheimer's disease workpackages lead
PI = Principal Investigator
EC = Executive committee member

1 Department of Psychiatry, School of Clinical Medicine, University of Cambridge, CB2 0SZ, UK
2 Cambridgeshire and Peterborough NHS Foundation Trust, Cambridge, CB21 5EF, UK
3 Sackler Centre, Institute of Health & Wellbeing, University of Glasgow, Sir Graeme Davies Building , Glasgow, G12 8TA, UK
4 Neuroscience, janssen Research & Development, Janssen Pharmaceutica NV, Turnhoutseweg 30, B-2340, Beerse, Belgium
5 The Maurice Wohl Clinical Neuroscience Institute, Cutcombe Road, London, SE5 9RT, UK

6 Neuroscience, Janssen Research & Development, LLC, Titusville, NJ, 08560, USA 7 Department of Psychiatry, University of Oxford, Warneford Hospital, Oxford, OX3 7JX, UK
8 Brighton & Sussex Medical School, University of Sussex, Brighton, BN1 9RR, UK
9 Sussex Partnership NHS Foundation Trust, Swandean, BN13 3EP, UK
10 Kings College London, Institute of Psychiatry, Psychology and Neuroscience, Department of Psychological Medicine, London, SE5 9RT, UK
11 Immuno-Psychiatry, Immuno-Inflammation Therapeutic Area Unit, GlaxoSmithKline R&D, Stevenage SG1 2NY, UK
12 MRC Cognition and Brain Sciences Unit, 15 Chaucer Road, Cambridge CB2 7EF, UK
13 Tanz Centre for Research in Neurodegenerative Diseases, 60 Leonard Avenue, Toronto, ON M5T 2S8 Canada
14 Department of Clinical Neurosciences, University of Cambridge, CB2 0SZ, UK 15 Cardiff University, Cardiff CF10 3AT, UK
16 NHS Greater Glasgow and Clyde, 1055 Great Western Rd, Glasgow G12 0XH, UK 17 University of Groningen, 9712 CP Groningen, Netherlands
18 Neurosciences Virtual PoC DPU, GlaxoSmithKline R&D, Stevenage SG1 2NY, UK
19 Experimental Medicine Imaging, GlaxoSmithKline R&D, Stevenage SG1 2NY, UK
20 King's College London, Department of Neuroimaging Sciences, Institute of Psychiatry, Psychology & Neuroscience, De Crespigny Park, London SE5 8AF, UK
21 H. Lundbeck A/S Ottiliavej 9, 2500, Valby, Denmark
22 University of Texas Health Science Center at San Antonio, 7703 Floyd Curl Dr, San Antonio, TX 78229, USA
23 NIHR Oxford cognitive health Clinical Research Facility, Warneford Hospital, Oxford, OX3 7JX, UK
24 The Kennedy Institute of Rheumatology, Roosevelt Dr, Oxford OX3 7FY, UK
25 Oxford Centre for Functional MRI of the Brain, John Radcliffe Hospital, Oxford OX3 9DU, UK
26 Pfizer, Inc, 1 Portland Street, Cambridge MA, USA
27 Centre for Biological Sciences, University of Southampton, Southampton, UK
28 Clinical Imaging Sciences Centre (CISC), University of Sussex, Brighton, BN1 9RR, UK
29 Sussex Partnership NHS Foundation Trust, Nevill Avenue, Hove BN3 7HZ, UK
30 Brighton & Sussex University Hospitals NHS Trust, Brighton BN2 5BE, UK
31 Department of Medicine, School of Clinical Medicine, University of Cambridge, CB2 0SZ, UK
32 VIB-KU Leuven Center for Brain & Disease Research, Campus Gasthuisberg, Herestraat 49, bus 602, 3000 Leuven, Belgium

*Former consortium members

Supplementary Figures

Rationale

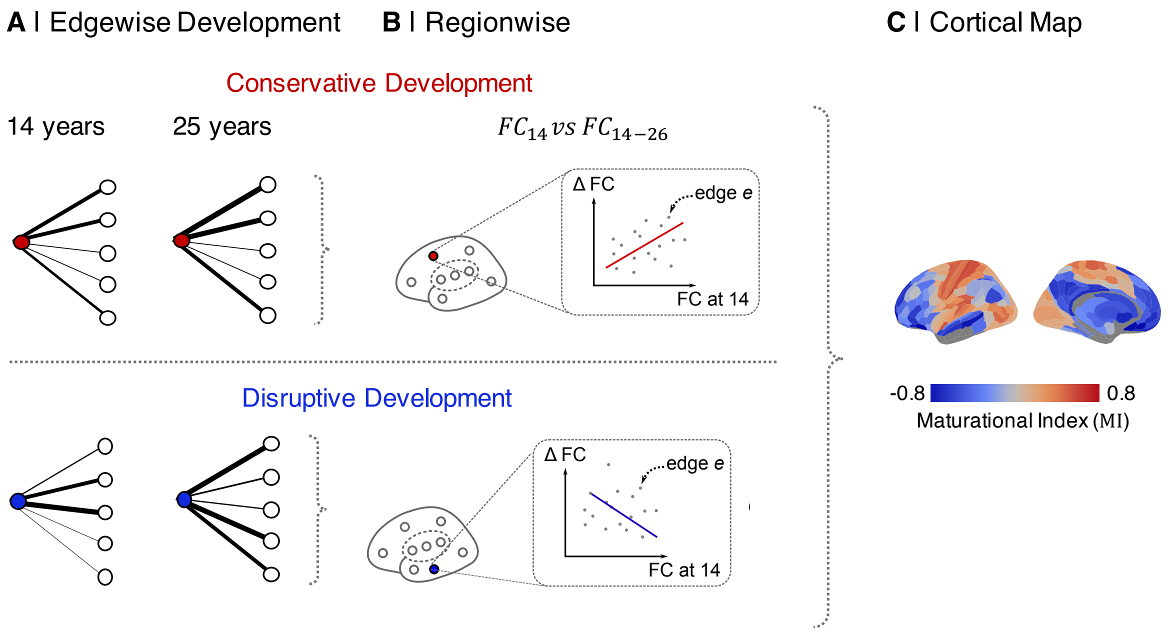

Fig. S1. Maturational Index Estimation:

In conservative development, a node's strong edges get stronger between 14 and 26 years (A; top). Thus, the Spearman correlation between baseline connectivity at age 14 (FC_{14}) and adolescent rate of change (FC_{14-26}) is positive (B, top). Conversely, in disruptive development, a node's weak edges get stronger over the course of adolescence, while its strong edges weaken (A, bottom). The correlation between FC_{14} and FC_{14-26} is negative. (C) Cortical map of regionwise MI. Thus, Maturational Index (MI) describes a 'system level' change in a node's wiring, reflecting maturational changes across all of a node's edges.

Data

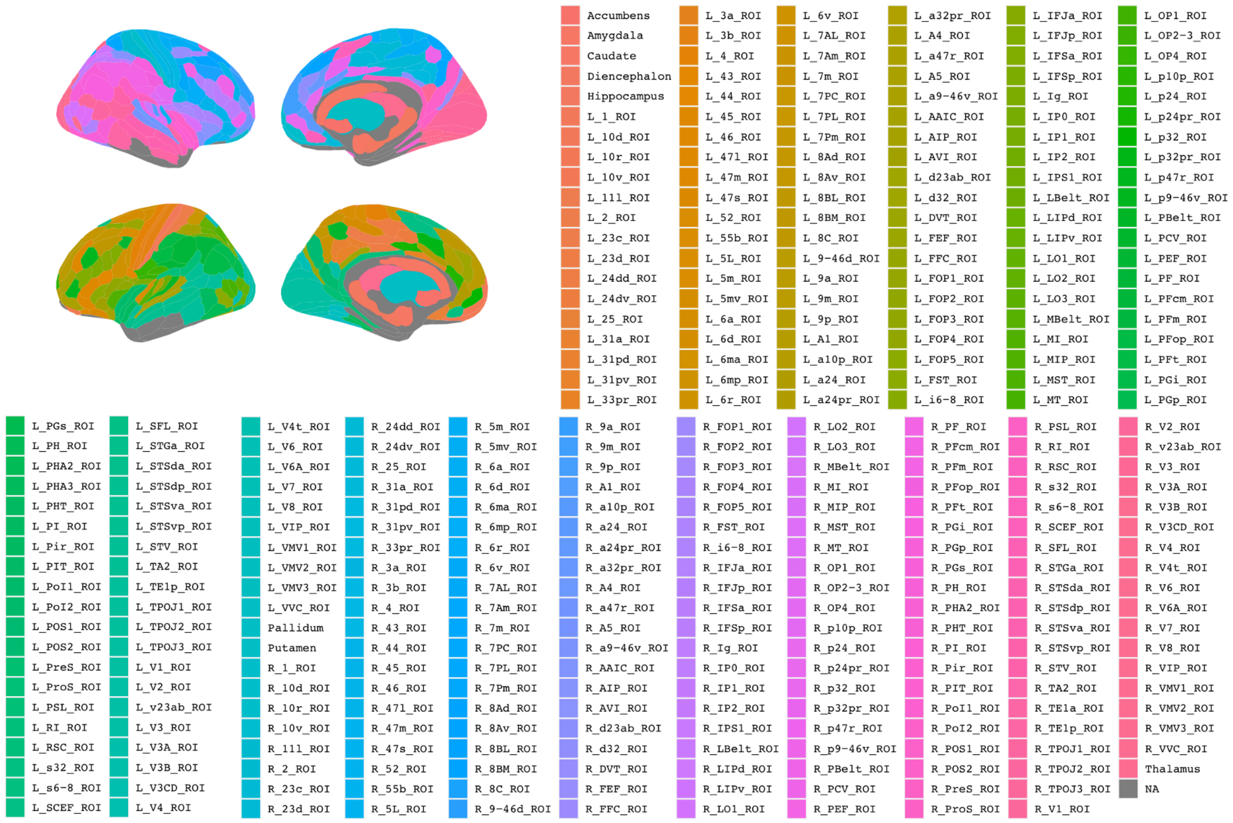

Fig. S2. Regional nodes included after preprocessing and quality control:

We parcellated the scans into 376 regional nodes (360 cortical regions in the Human Connectome Atlas, and 16 subcortical regions from the Freesurfer Software). 30 cortical regions were excluded due to low regional mean signal ($Z < -1.96$) in at least one scan. These regions were: L 10pp, L 13l, L OFC, L EC, L H, L PeEc, L PHA1, L TGd, L TE1a, L TE2a, L TF, L TE2p, L pOFC, L TGv, L TE1m, R 10pp, R 13l, R OFC, R EC, R H, R PeEc, R PHA1, R PHA3, R TGd, R TE2a, R TF, R TE2p, R pOFC, R TGv, R TE1m.

Head Motion

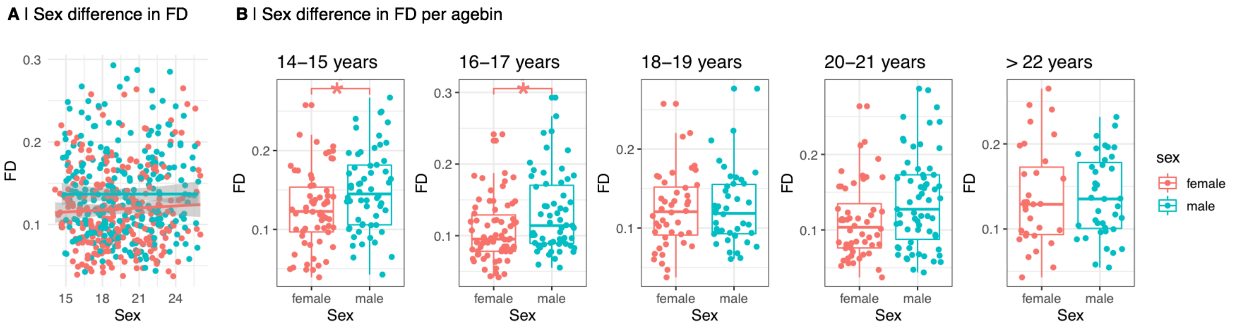

Fig. S3. Sex differences in framewise displacement:

(A) Across all age-related strata, males showed higher framewise displacement (FD) than females ($P_{sex} < 0.05, t(296) = 3.25$). (B) Males showed increased FD in the first two age-related strata ($P_{sex} < 0.05$), however the effect did not survive correction for multiple comparisons.

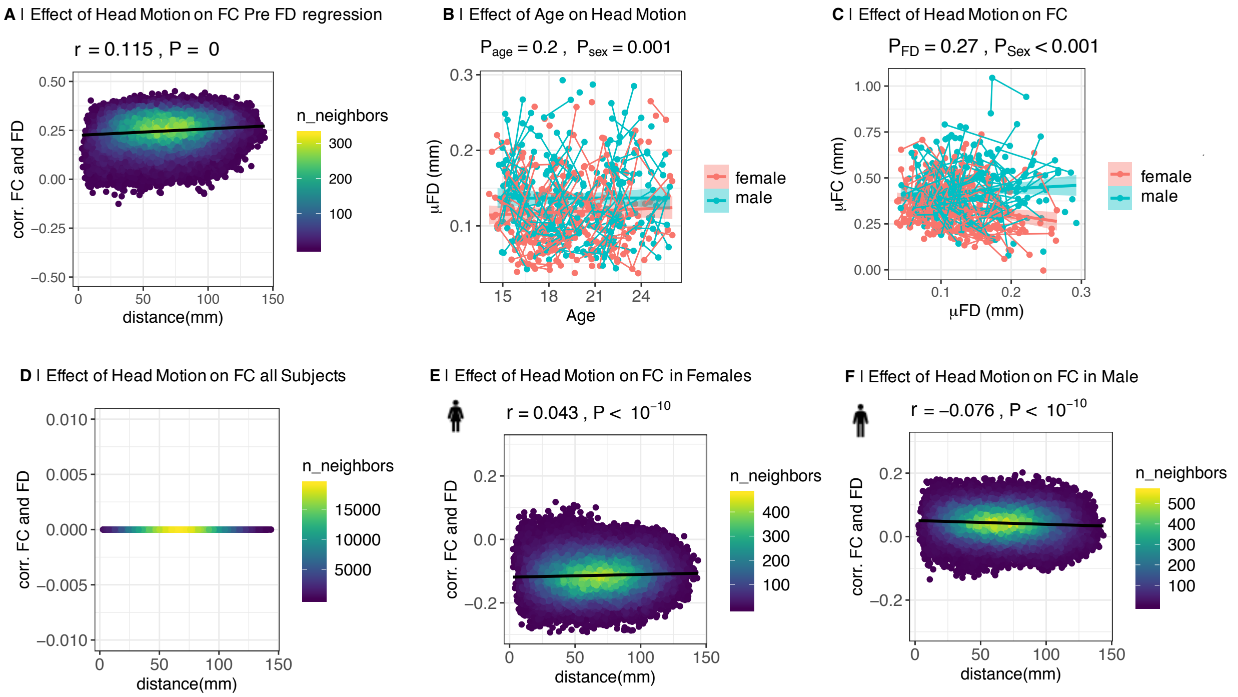

Fig. S4. Effect of head motion (FD) on functional connectivity (FC) in the original sample:

(A) After ME-ICA preprocessing, we found a weak relationship between the correlation of FC and head motion (across participants) and the Euclidean distance spanned by edges. Further, the average edge-wise correlation between FC and motion is non-zero. (B) To remove the dependence of FC on motion in our sample, mean FD was regressed from each edge; the residuals constitute participant-specific FD-corrected FC, with intercepts retained to maintain the relative importance of edges across the group as well as the interpretability of FC values. Thus, in the full sample, average head motion, quantified as mean frame-wise displacement (FD), did not change with age ($P_{age} = 0.2, t(220) = 1.25$). However, there was a weak, but significant effect of sex on FD ($\beta_{sex} = 0.02, P_{sex} < 0.01, t(296) = 3.3$). (C) The effect of participants' motion (across participants) on global FC was not significant ($P_{FD} = 0.27$). (D) By definition, there was no effect of distance on the correlation between FC and motion, and the average edge-wise correlation between FC and motion was non-zero (as evidenced by a non-zero intercept of the linear regression on the y-axis). (E) However, since our motion correction was performed across all subjects, we still observed weak, but significant effects of distance on the correlation of FC and FD for females ($r = 0.04, P < 0.001$) (F) and males ($r = -0.08, P < 0.001$) (G) separately.

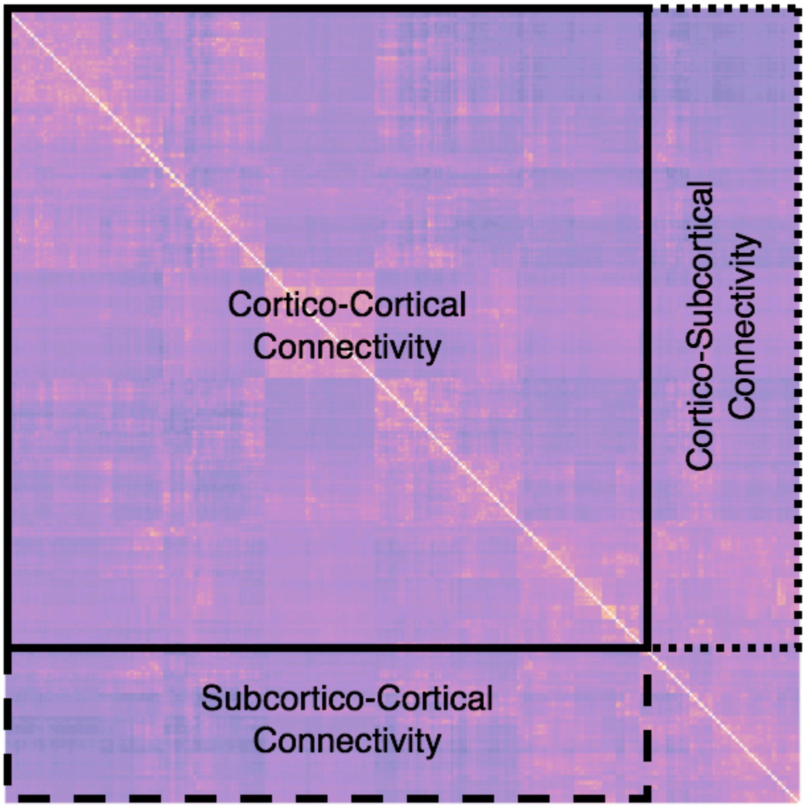

Fig. S5. Functional connectivity estimation:

We estimated functional connectivity by averaging the strength of each node's edges across different parts of the matrix: (1) cortico-cortical connectivity was estimated as the mean over a cortical node's connections with all 329 other cortical nodes; (2) cortico-subcortical connectivity was estimated as the mean over a cortical node's connections with all 16 subcortical nodes; (3) subcortico-cortical connectivity was estimated as the mean over a subcortical node's connections with all 330 cortical nodes.

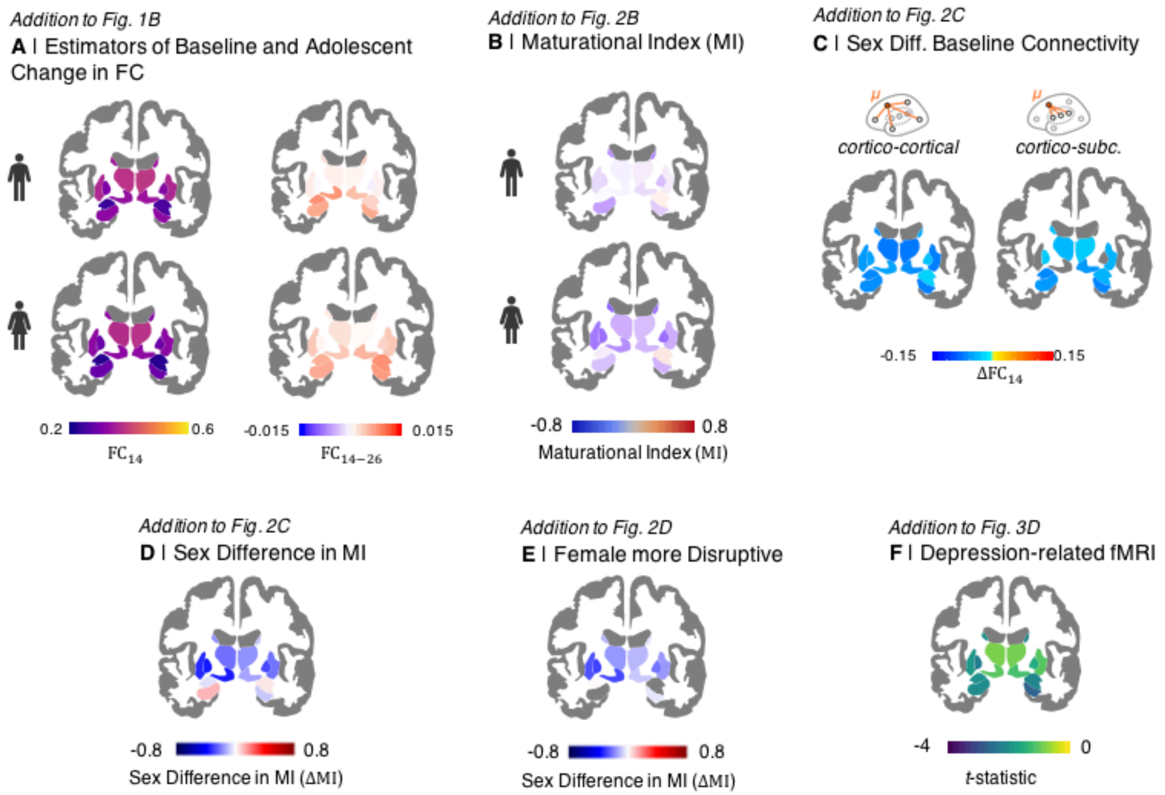

Fig. S6. Additional Subcortical Plots:

In order to facilitate visual examination of subcortical results, we are displaying additional subcortical maps for all brain maps in the main manuscript. (A) Male (top) and female (bottom) estimators of baseline connectivity and adolescent rate of change (ref. Fig. 1B). (B) Maturational Index for males (top) and females (bottom) separately (ref. Fig 2B). (C) Cortico-cortical and cortico-subcortical sex differences in baseline connectivity (ref. Fig 2C). (D) Sex difference in maturational index (MI; ref. Fig 2C). (E) Female more disruptive ROIs (ref. Fig. 2D). (F) MDD case-control map (*t*-statistic; ref. Fig. 3D).

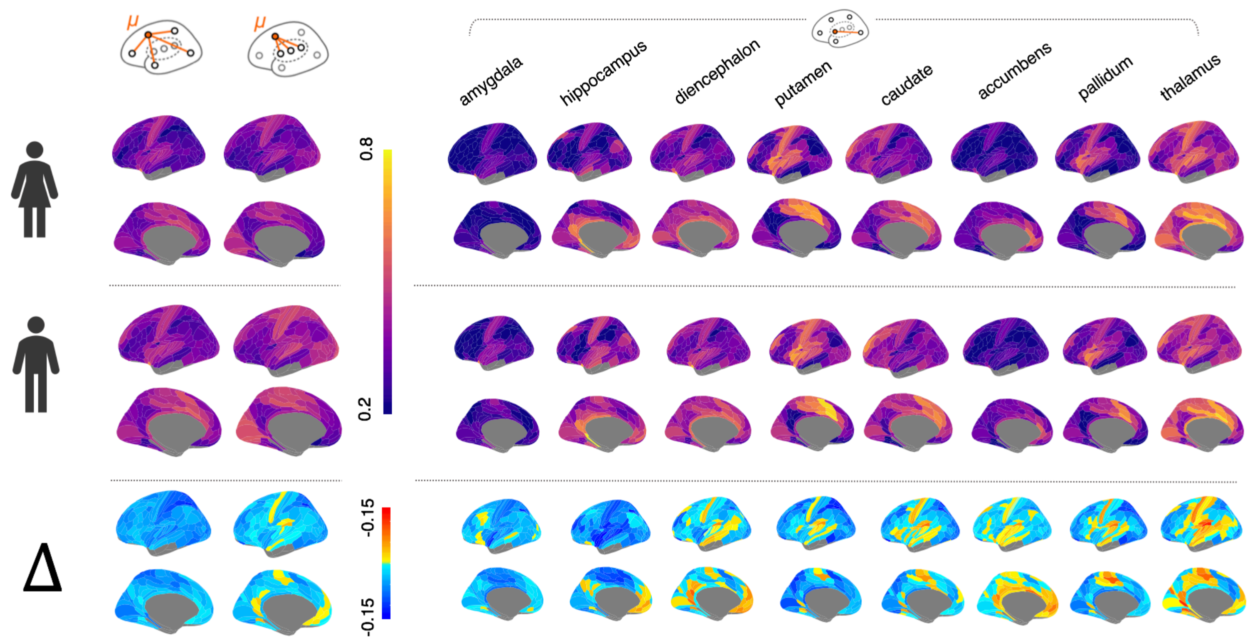

Fig. S7. All baseline connectivity FC_{14} plots:

Separate linear mixed effects models were fit for both sexes to model regional functional connectivity development as predicted by age and site for cortical-cortical, cortical-subcortical and subcortical-cortical connections. Predicted adolescent baseline connectivity at baseline, FC_{14}, was estimated using these models for males and females and a difference map was constructed ($\Delta FC_{14} = FC_{14_{female}} - FC_{14_{male}}$).

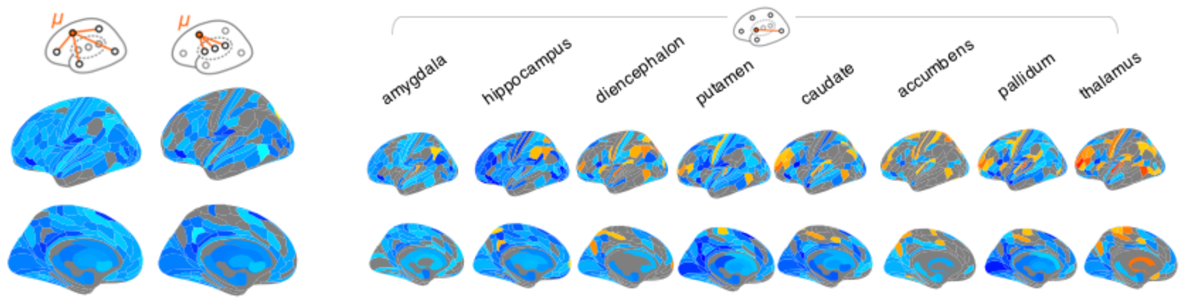

Fig. S8. Significance of sex difference in baseline connectivity FC_{14}:

Here, we thresholded the map of sex differences in FC_{14} from Fig. S7 (bottom) for significance after FDR-correction at $P < 0.05$. We are showing all plots with more than two significant regional nodes.

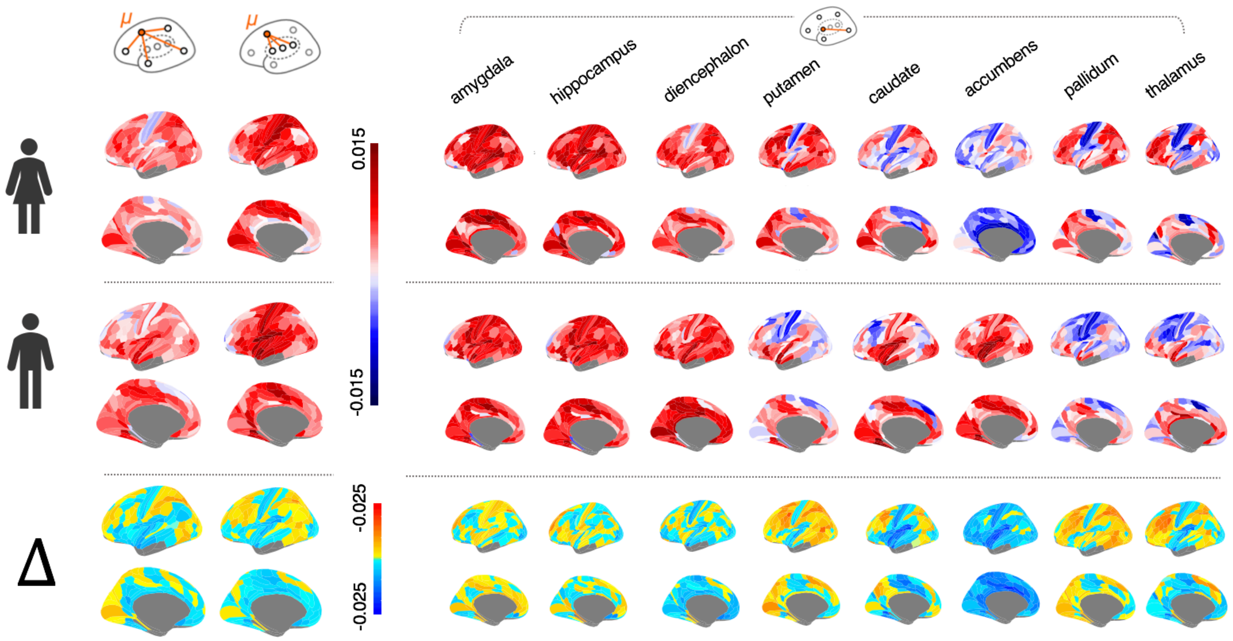

Fig. S9. All adolescent rate of change FC_{14-26} plots:

The adolescent rate of change, FC_{14-26}, was estimated as the β_{age} coefficient from the linear mixed effects model. We constructed a map of sex differences in FC_{14-26} ($\Delta FC_{14-26} = FC_{14-26_{female}} - FC_{14_{-26male}}$).

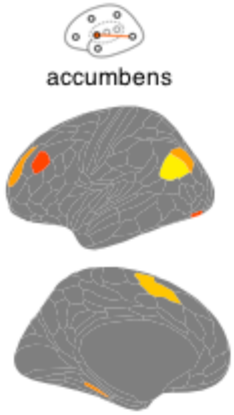

Fig. S10. Significance of sex difference in adolescent rate of change ΔFC_{14-26}:

Here, we thresholded the map of sex differences in FC_{14-26} from Fig. S7 (bottom) for significance after FDR-correction at $P < 0.05$. Only the accumbens shows significant sex differences after FDR-correction.

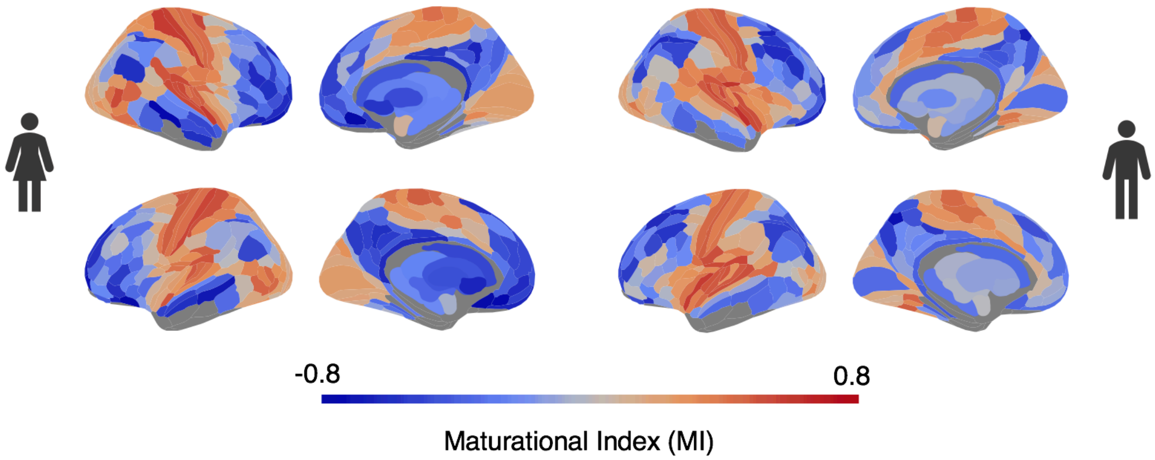

Fig. S11 All maturational index plots:

Maturational index for males and females separately, with both hemispheres of the brain displayed.

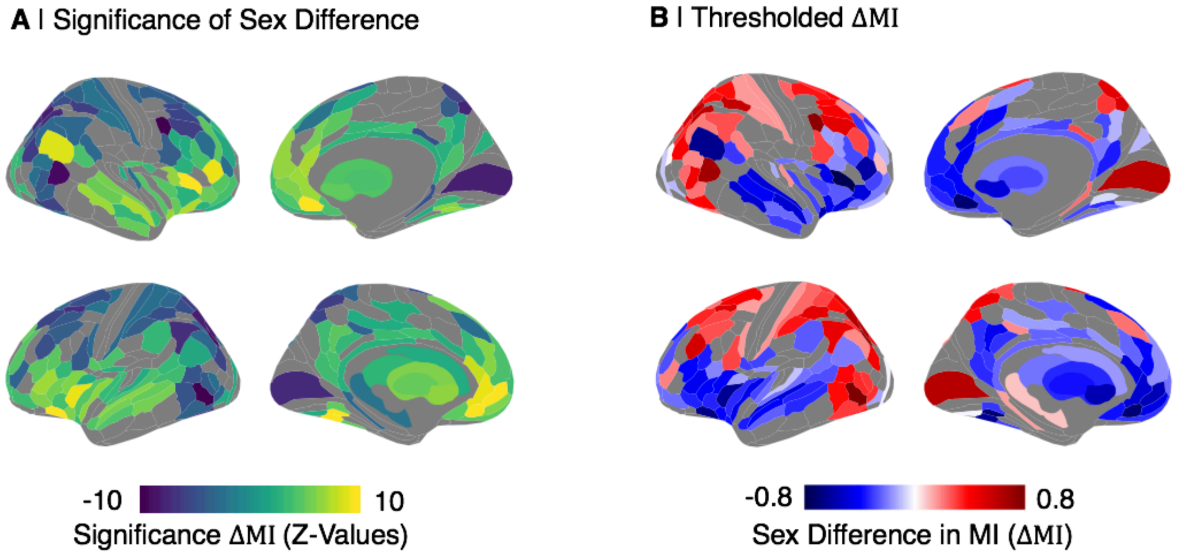

Fig. S12. Significance of Sex Difference in MI:

We estimated the significance of the sex difference in MI in a parametric approach (77), by recalculating MI for each sex as a linear regression of edge-wise FC_{14} on FC_{14-26} and testing for the equivalence of the slopes, using their standard errors (SE). (A) 230 regional nodes displayed significantly sex divergent behaviour ($P(\Delta\text{MI} = 0) < 0.05$). (B) ΔMI thresholded by significance ($P(\Delta\text{MI} = 0) < 0.05$).

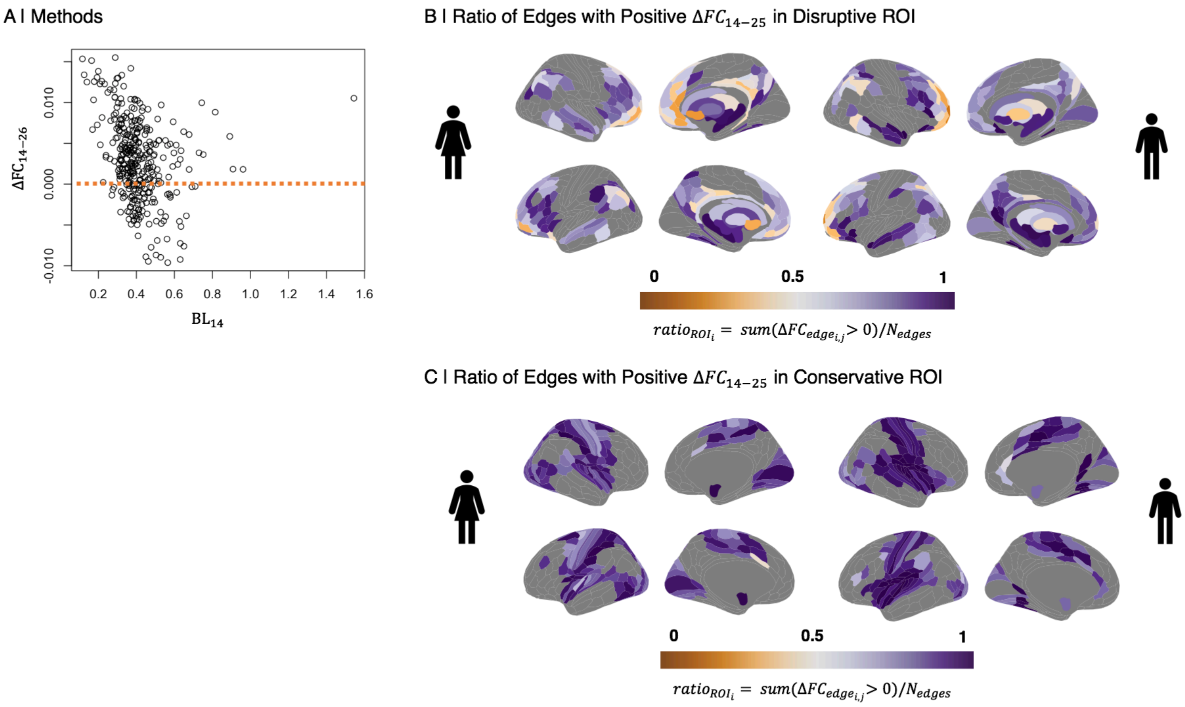

Fig. S13. Trends in disruptive and conservative development of connectivity:

Disruptive development in a region can mean one of two trends: Either a region is strong at baseline and loses strength over the course of adolescence ("strong getting weaker"), or it is weak at baseline and gains strength ("weak getting stronger"). Conservative development, in turn, can mean that either a region is strong at baseline and gains strength over the course of adolescence ("strong getting stronger"), or it is weak at baseline and loses strength ("weak getting weaker"). (A) Here, we estimate these trends for regions of disruptive and conservative change in each sex by calculating the ratio of edges with a positive adolescent rate of change connected to a node ($ratio_{ROI_i} = sum(\Delta FC_{edge_{ij}} > 0)/N_{edges}$). We then thresholded this ratio map for disruptive and conservative nodes in each sex. (B) In disruptive regions, if this $ratio_{ROI_i} > 0.5$, a region is 'strong getting weaker', if $ratio_{ROI_i} < 0.5$, it is "weak getting stronger". We find that disruptive regions are predominantly characterized by 'weak getting stronger' changes (78.5% of regions in females and 81.3% in males). (C) In conservative regions, if this $ratio_{ROI_i} > 0.5$, a region is 'strong getting strong', if $ratio_{ROI_i} < 0.5$, it is 'weak getting weaker'. We find that all regions in both sexes display 'strong getting stronger' trends only.

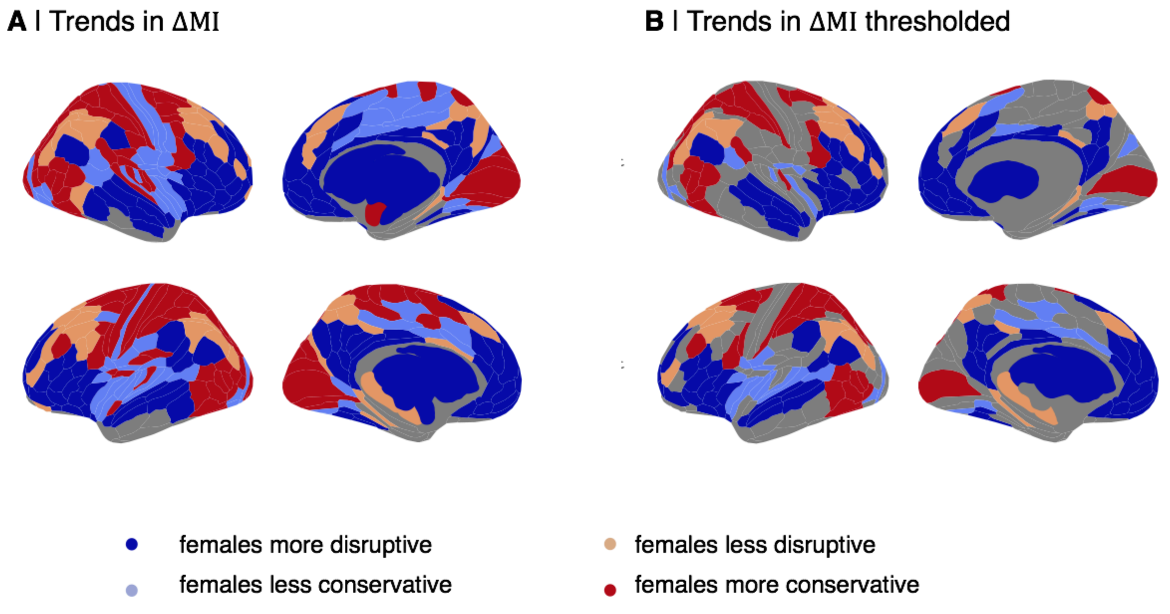

Fig. S14. Trends in negative ΔMI:

A negative ΔMI in a region can mean one of two trends: females show (1) more disruptive, or (2) less conservative development than males. Similarly, a positive ΔMI in a region can mean one of two trends: females show (1) more conservative, or (2) less disruptive development than males. Here, we disentangle these trends for all regions. (A) Trends in ΔMI, unthresholded. (B) Trends in ΔMI, thresholded by significance of sex difference in ΔMI ($P(\Delta \mathrm{MI} = 0) < 0.05$).

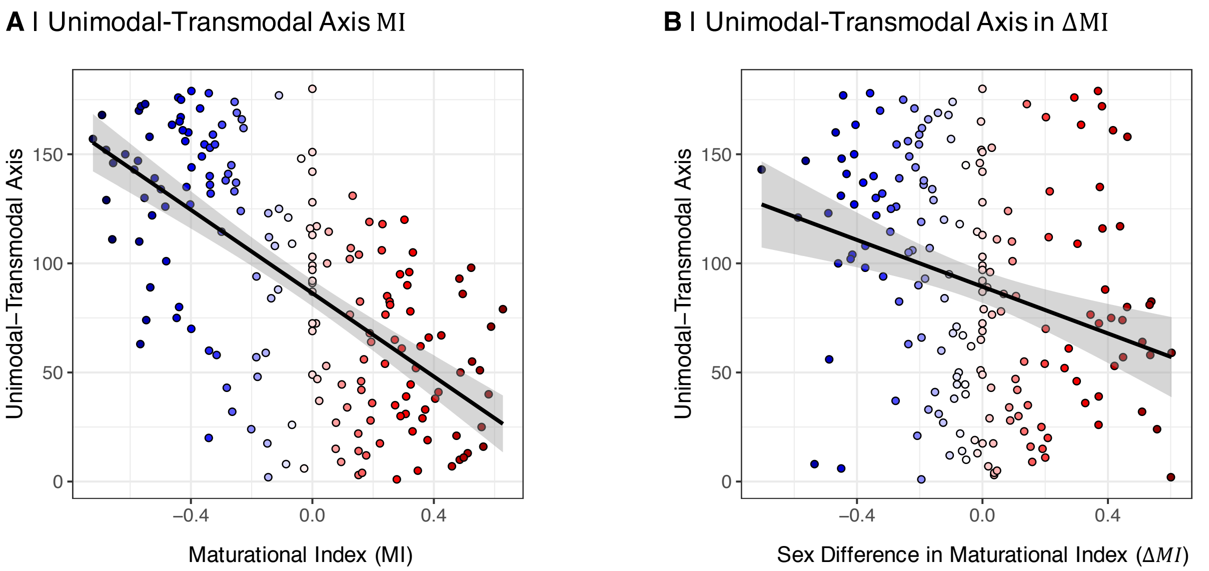

Fig. S15. Unimodal-Transmodal Axis was correlated with MI and ΔMI:

Co-location of the unimodal-transmodal (U-T) axis defined by Sydnor et al (2021) (*46*) with maps of MI and ΔMI. (A) Scatterplot of U-T axis position (y-axis) versus MI for each cortical region, with points colour-coded according to the sign of MI. The MI map was significantly co-located with the U-T axis map (MI; r =- 0.63, $P < 0.01$, $P_{spin} < 0.01$). Transmodal cortical regions, with high scores on the U-T axis, tended to have more negative MI, indicating disruptive development. (B) Scatterplot of U-T axis position (y-axis) versus ΔMI for each cortical region, with points colour-coded according to the sign of ΔMI. The ΔMI map was also negatively correlated with the U-T map but the co-location was not robust to significance testing that controlled for spatial auto-correlation (ΔMI; r = -0.28, $P < 0.01$, P_{spin}= 0.08). Transmodal cortical regions, with high scores on the U-T axis, tended to have negative ΔMI, indicating more disruptive development in females.

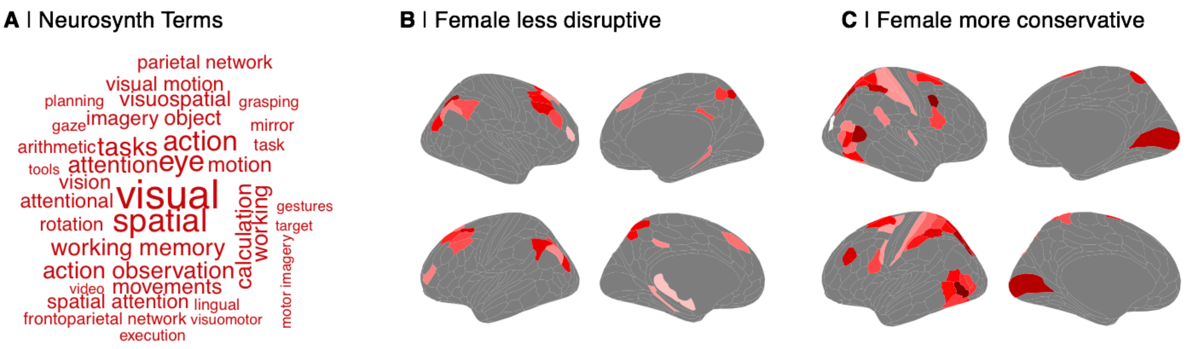

Fig. S16. Positive ΔMI:

As in our main analysis of regions displaying a negative ΔMI, here we analysed regions with female > male conservative development indicated by positive ΔMI. (A) Neurosynth terms associated with regions positive of positive ΔMI. We uploaded the map of positive ΔMI regions to Neurosynth and found they are located in cortical areas that were activated by motor and sensory tasks. We further analysed (B) regions with a significant sex difference in ΔMI ($P(\Delta\text{MI} = 0) < 0.05$) which displayed female less disruptive development and (C) regions with a significant sex difference in ΔMI ($P(\Delta\text{MI} = 0) < 0.05$) which displayed female more conservative development. We find those regions are primarily located in association cortical areas.

Enrichment Analysis

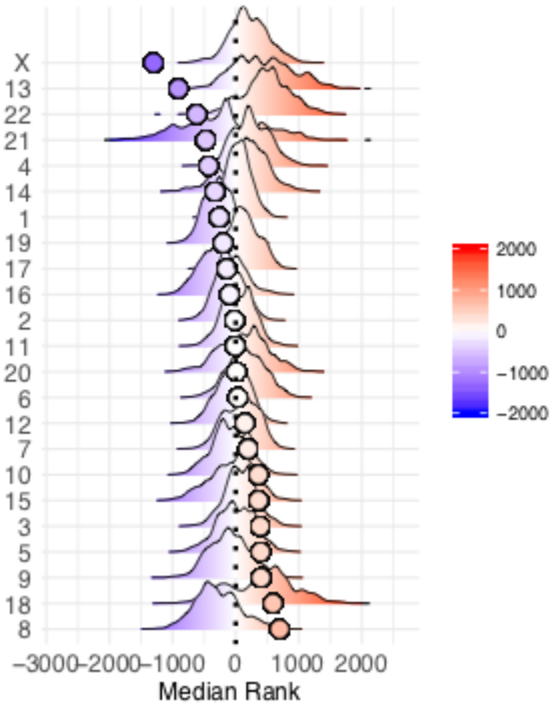

Fig. S17. Chromosomal null models:

We assessed chromosomal enrichment of the ranked genes weighted on PLS1 to test our hypothesis that sex chromosomal gene expression is related to the sexual differences in adolescent brain development. We used a median gene rank approach to analyse an enrichment of PLS1 for genes located on specific chromosomes. Here, we show the permutation distributions of the median rank for 10,000 random gene sets for each chromosome. We overlay the true median gene rank as a dot on each distribution.

A | Cell Type Enrichment

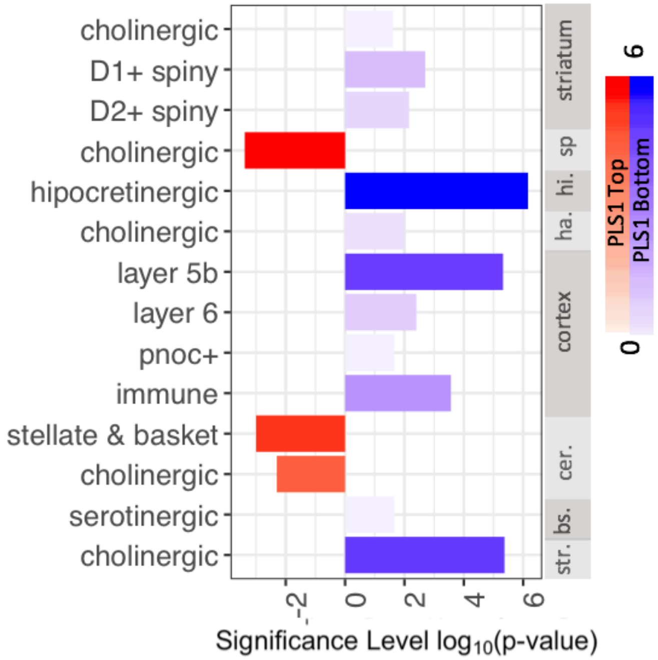

Fig. S18. Cell type enrichment:

We uploaded the list of significantly positive and negative ($P_{FDR} < 0.05$) genes, respectively, to the CSEA tool's *specific expression analysis across cell types* function and found ΔMI was associated with two separate transcriptomic signatures of gene expression. The bottom ($Z < -2.58$) of the ranked list of genes weighted on PLS1 was enriched for cholinergic and serotonergic cells across brain structures, as well as immune cells in the cortex, while the top ($Z > 2.58$) of the ranked list of weighted genes on PLS1 was more sparsely associated with cholinergic and stellate and basket cells in the cerebellum and cholinergic cells in the spinal cord.

Co-Location with Depression

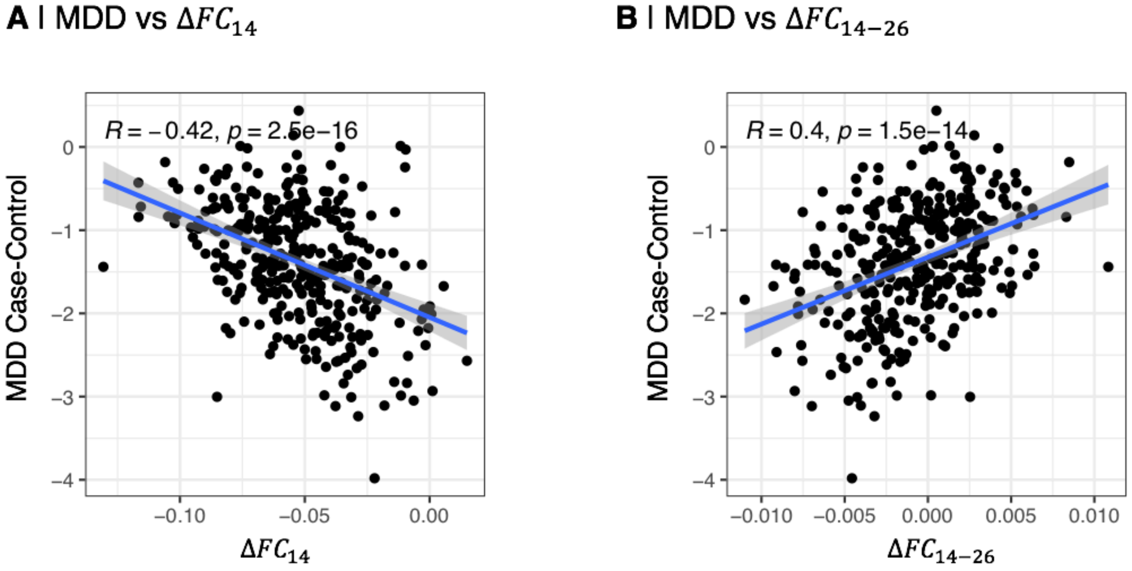

Fig. S19. Specificity of Anatomical Co-Location with Depression

Correlation between sex differences in the parameters of adolescent brain development and the MDD case-control map. We find there is a correlation between MDD case-control differences and (A) the sex differences in baseline connectivity (ΔFC_{14}; $r = 0.42, P < 0.001, P_{spin} < 0.001$) and (B) the sex differences in the adolescent rate of change (ΔFC_{14-26}; $r = 0.4, P < 0.001, P_{spin} < 0.001$). Maturational Index (MI) is estimated as the correlation between a region's edgewise FC_{14} and FC_{14-26}. Thus, Maturational Index, $FC_{14}$and FC_{14-26}, and by extension the sex differences between them, are by definition closely related. Therefore, it is not surprising that there is a correlation between the MDD case-control map and both the sex differences in baseline connectivity and the sex differences in adolescent rate of change.

Sensitivity Analyses

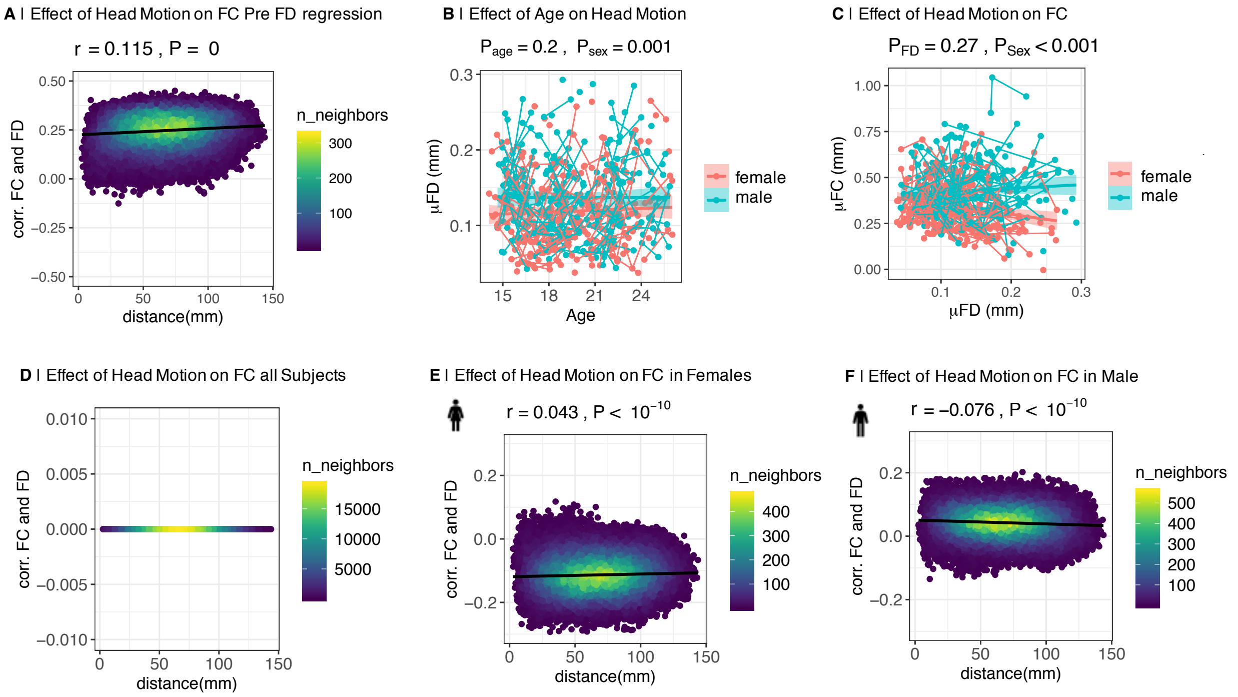

Fig. S20. Effect of head motion (FD) on functional connectivity (FC) in the FD regression by sex sample:

To remove the sex difference in the dependence of FC on motion, mean FD was regressed from each edge for males and females separately; the residuals constitute participant-specific FD-corrected FC, with intercepts retained to maintain the relative importance of edges across the group as well as the interpretability of FC values. (A) Following this correction, subjects' average head motion, quantified as mean frame-wise displacement (FD), did not change with age ($P_{age} = 0.21, t(220) = 1.25$). However, there was a weak, but significant effect of sex on FD ($P_{sex} < 0.01, t(296) = 3.266$). (B) Mean participant motion was not related to mean FC across participants ($P_{FD} = 0.93, t(221) = 0.09$) and there was no effect of sex on the relationship ($P_{Sex} = 0.93, t(296) = -0.05$). (C) The correlation between FC at each edge and participant motion shows a weak but significant relationship with the Euclidean distance spanned by edges ($r = 0.05, P < 0.001$). The average edge-wise correlation between FC and motion is very close to zero (intercept = -0.002). By definition, the correlation between FC and motion almost completely vanished at the level of individual edges for males and females separately (D) and (E).

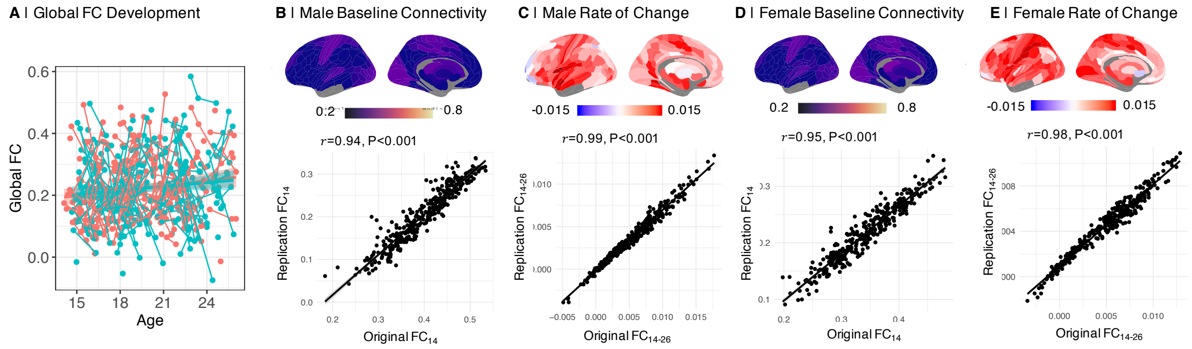

Fig. S21. Replication of key elements of Fig. 1 in sample where FD was regressed per sex group:

Sex and age effects on functional connectivity (FC) were modeled using linear mixed effects models on different spatial scales. (A) Global FC increased with age ($t(219) = 2.94, P_{Age} < 0.05$). There was no significant effect of sex on global connectivity ($P_{Sex} = 0.73$). (B)-(E) baseline connectivity at age 14 (FC_{14}) and adolescent rate of change (FC_{14-26}) in males and females were qualitatively and quantitatively highly consistent with our main results.

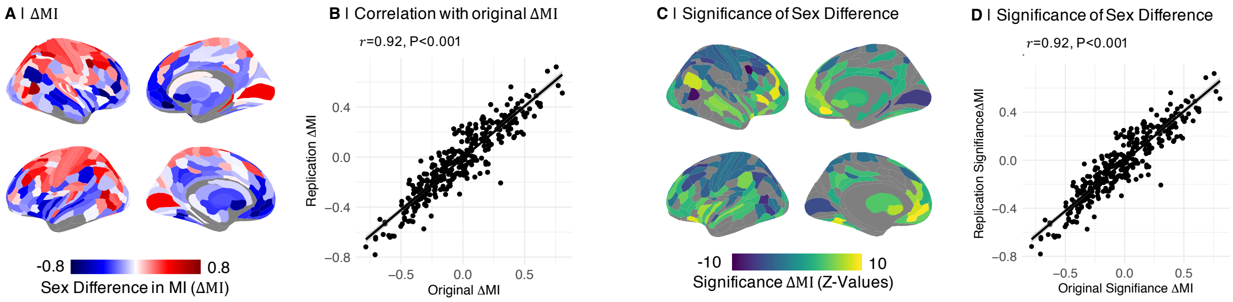

Fig. S22. Replication of key elements of Fig. 2 in sample where FD was regressed per sex group:

(A) Sex difference in maturational index where FD was regressed per sex group: (ΔMI). (B) The original and replication ΔMI map were significantly correlated (r = 0.92, P < 2.2e-16) (C) The sex difference in MI was significant in 219 regional nodes (α = 0.01; -2.57 < Z < 2.57). (D) The significance map of sex differences in maturational index was significantly correlated with the original map (r = 0.92, P < 2.2e-16).

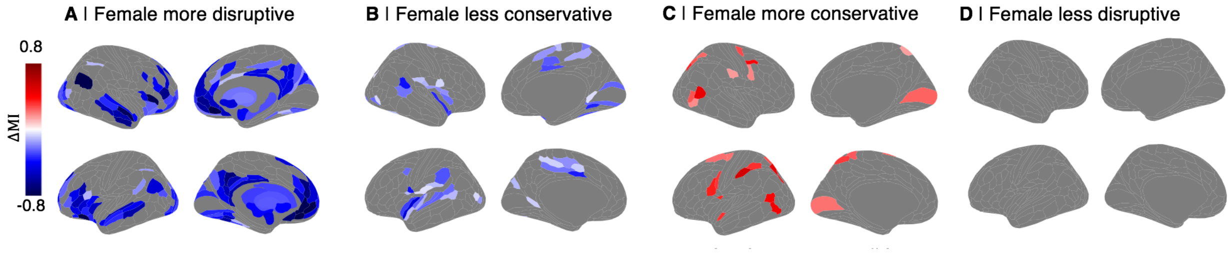

Fig. S23. Trends in the sex difference in maturational development (ΔMI) in the sample where FD was regressed per sex group:

Trends in ΔMI, thresholded for regions with significant sex differences ($P_{FDR} < 0.05$).

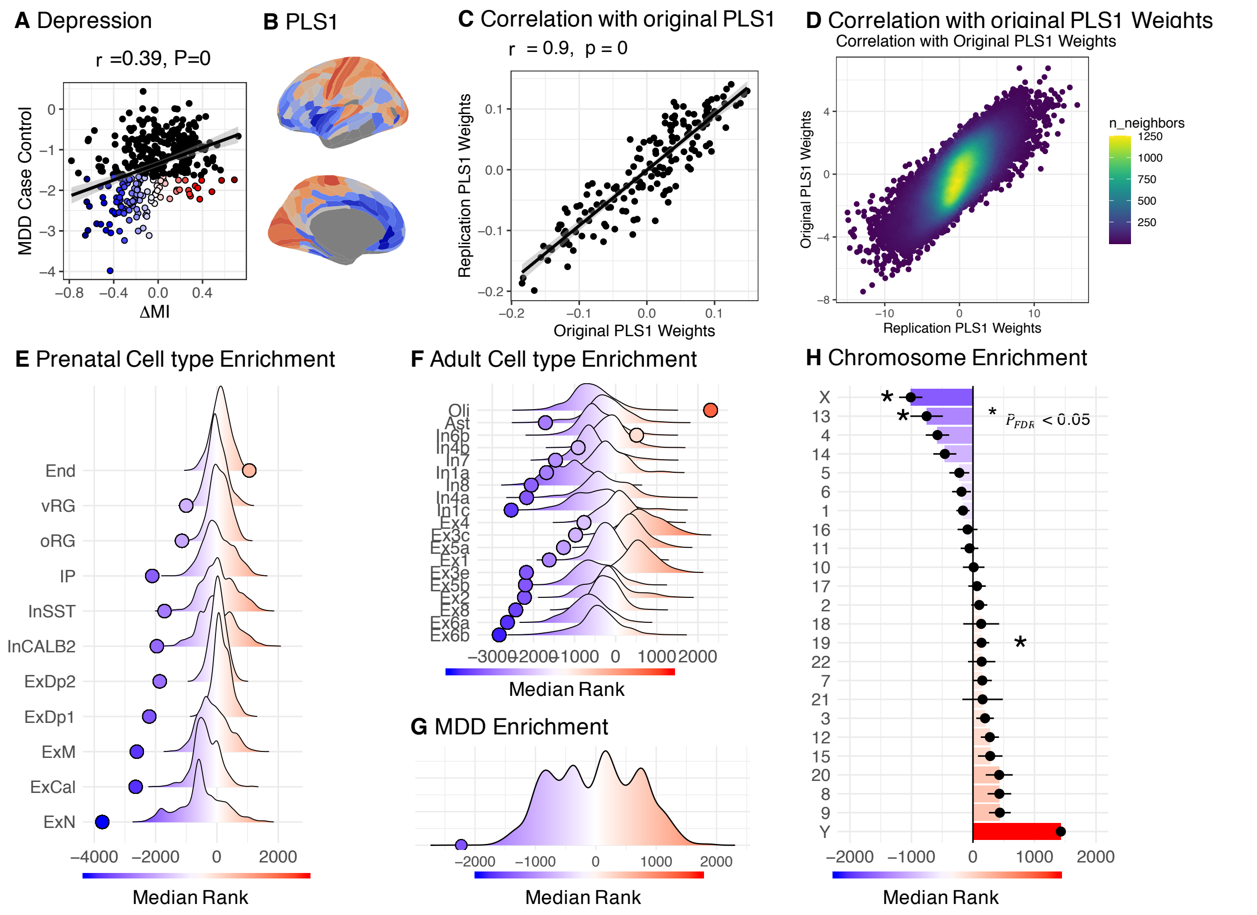

Fig. S24. Replication of key elements of Figs. 3 and 4 in the sample where FD was regressed per sex group:

(A) Correlation of ΔMI estimated from the sample where FD was regressed by sex with an MDD case-control map ($r = 0.39, P < 0.01, P_{spin} < 0.001$). Each point represents one of 346 cortical or subcortical regions; ROIs that show a significant MDD case-control difference, $t \neq 0$, and a significant sex difference in $\Delta MI, t \neq 0$ are highlighted. We used partial least squares (PLS) regression to map the Allen Human Brain Atlas (AHBA) gene expression data (*49*) onto the ΔMI map. (B) PLS1 component. (C) The replication PLS1 was significantly correlated ($r = 0.9, P < 0.01$) with the original PLS1. (D) Correlation of ICV-controlled PLS1 weights with the original PLS1 weights. (E) Enrichment analysis for prenatal cell type-specific genes. Negatively weighted genes (blue) were significantly enriched for genes expressed by prenatal radial glia (vRG, oRG), somatostatin interneurons (InSST) and excitatory neurons. (F) Enrichment analysis for adult cell type-specific genes. Negatively weighted genes were significantly enriched for genes expressed by adult astrocytes, and various inhibitory and excitatory neurons. Positively weighted genes were enriched for oligodendrocytes. (G) Enrichment analysis for MDD-related genes. Negatively weighted genes were significantly enriched for genes associated with major depressive disorder (*57*). (H) Enrichment analysis

for chromosomal genes. Plot of the median rank of genes from each chromosome on PLS1, with standard deviations.

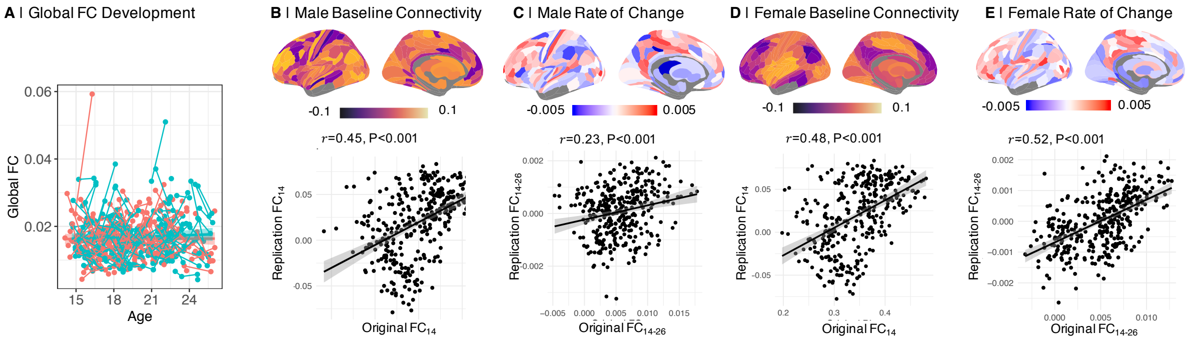

Fig. S25. Replication of key elements of Fig. 1 in the GSR sample:

Sex and age effects on functional connectivity (FC) were modeled using linear mixed effects models on different spatial scales. (A) We did not find significant effects of sex ($t(296) = 0.82, P = 0.41$) or age ($t(219) = 0.3, P = 0.77$) on global FC in the GSR sample. (B)-(E) The baseline connectivity at age 14 (FC_{14}) and adolescent rate of change (FC_{14-26}) in males and females in the GSR sample were quantitatively and qualitatively similar to the original maps.

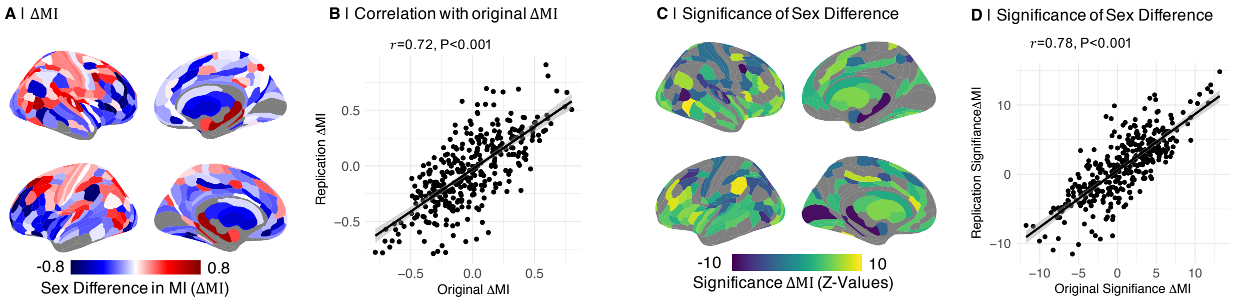

Fig. S26. Replication of key elements of Fig. 2 in the GSR sample:

(A) Sex difference in maturational index in the GSR sample (ΔMI). (B) The original and replication ΔMI map were significantly correlated (r = 0.72, P < *0.001*) (C) The sex difference in MI was significant in 229 regional nodes (α = 0.01; -2.57 < Z < 2.57). (D) The significance map of sex differences in maturational index was significantly correlated with the original map (r = 0.78, P < *0.001*).

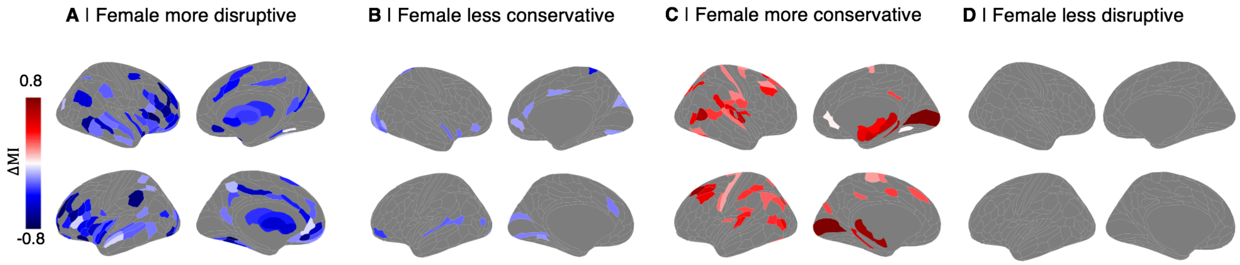

Fig. S27. Trends in the sex difference in maturational development (ΔMI) in the GSR sample:

Trends in ΔMI, thresholded for regions with significant sex differences ($P_{FDR} < 0.05$).

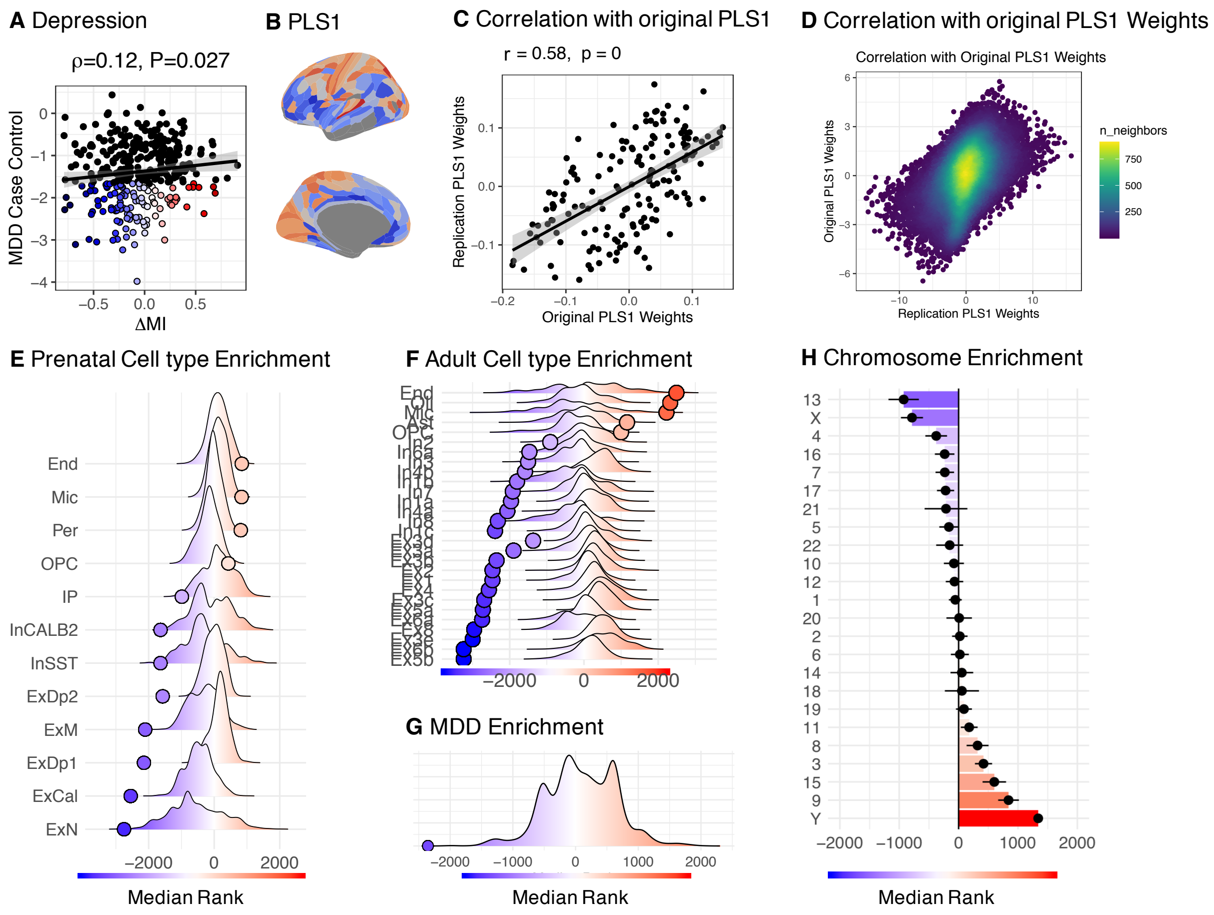

Fig. S28. Replication of key elements of Figs. 3 and 4 in the GSR sample:

(A) Correlation of GSR-processed ΔMI with an MDD case-control map ($r = 0.12, P < 0.05, P_{spin} = 0.06$). Each point represents one of 346 cortical or subcortical regions; ROIs that show a significant MDD case-control difference, $t \neq 0$, and a significant sex difference in ΔMI, $t \neq 0$ are highlighted. We used partial least squares (PLS) regression to map the Allen Human Brain Atlas (AHBA) gene expression data (*49*) onto the ΔMI map. (B) PLS1 component. (C) The GSR-processed PLS1 was significantly correlated ($r = 0.58, P < 0.001$) with the original PLS1. (D) Correlation of GSR processed PLS1 weights with the original PLS1 weights. (E) Enrichment analysis for prenatal cell type-specific genes. Negatively weighted genes (blue) were significantly enriched for genes expressed by prenatal somatostatin interneurons (InSST) and excitatory neurons and positively weighted genes were enriched for radial glia (vRG, oRG). (F) Enrichment analysis for adult cell type-specific genes. Negatively weighted genes were significantly enriched for genes expressed by adult inhibitory and excitatory neurons. Positively weighted genes were enriched for oligodendrocytes, endothelial cells and microglia. (G) Enrichment analysis for MDD-related genes. Negatively weighted genes were significantly enriched for genes associated with major depressive disorder by an independent genome wide association study (*57*). (H) Enrichment analysis for chromosomal genes. Plot of the median rank of genes from each chromosome on PLS1, with standard deviations.

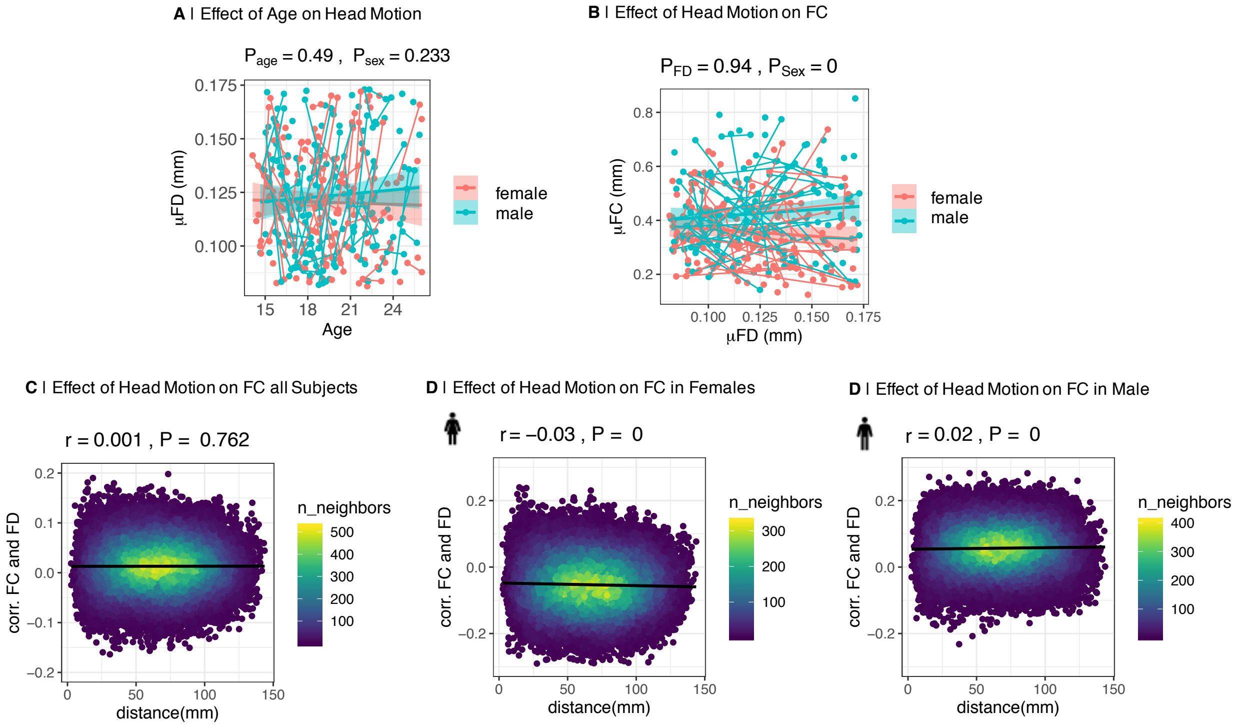

Fig. S29. Effect of head motion (FD) on functional connectivity (FC) in the motion-matched sample:

The motion-matched sample is a subsample of the full data set, in which we removed the dependence of FC on motion in our sample by regressing FD from each edge; the residuals constitute participant-specific FD-corrected FC, with intercepts retained to maintain the relative importance of edges across the group as well as the interpretability of FC values. (A) In this subsample, average head motion, quantified as mean frame-wise displacement (FD), did not change with age ($P_{age} = 0.49$). And there was no effect of sex on FD ($P_{sex} = 0.23$). (B) The effect of participants' motion (across participants) on global FC was not significant ($P_{FD} = 0.94$). (C) There was no effect of distance on the correlation between FC and motion ($r = 0.001, P = 0.76$), and the average edge-wise correlation between FC and motion was almost zero (intercept = 0.01). (D) However, since our motion correction was performed across all subjects in the full sample, we still observed weak, but significant effects of distance on the correlation of FC and FD for females ($r = -0.03, P < 0.001$) (D) and males ($r = 0.02, P < 0.001$) (E) separately, and the average edge-wise correlation between FC and motion was non-zero ($intercept_{females} = -0.02, intercept_{males} = 0.05$).

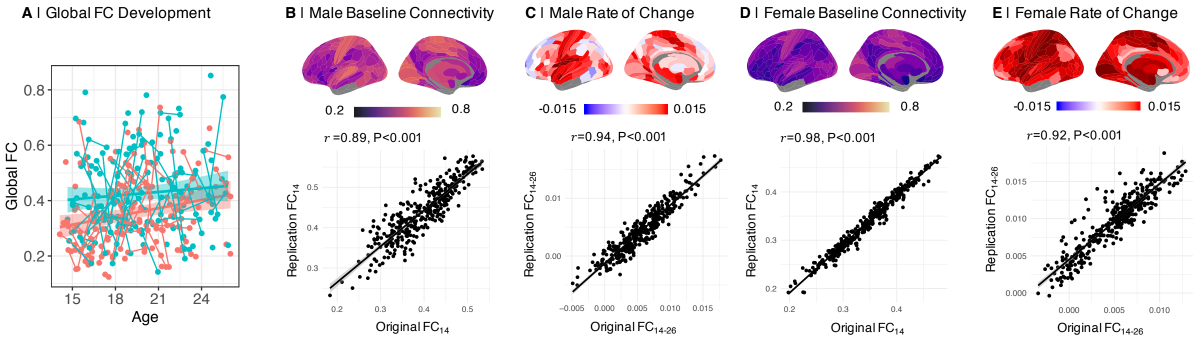

Fig. S30. Replication of key elements of Fig. 1 in the motion-matched sample:

Sex and age effects on functional connectivity (FC) were modeled using linear mixed effects models on different spatial scales. (A) Global FC increased with age ($t(94) = 2.48, P < 0.05$) and was higher in males ($t(215) = 3.84, P < 0.001$). (B)-(E) baseline connectivity at age 14 (FC_{14}) and adolescent rate of change (FC_{14-26}) in males and females were qualitatively and quantitatively highly consistent with our main results.

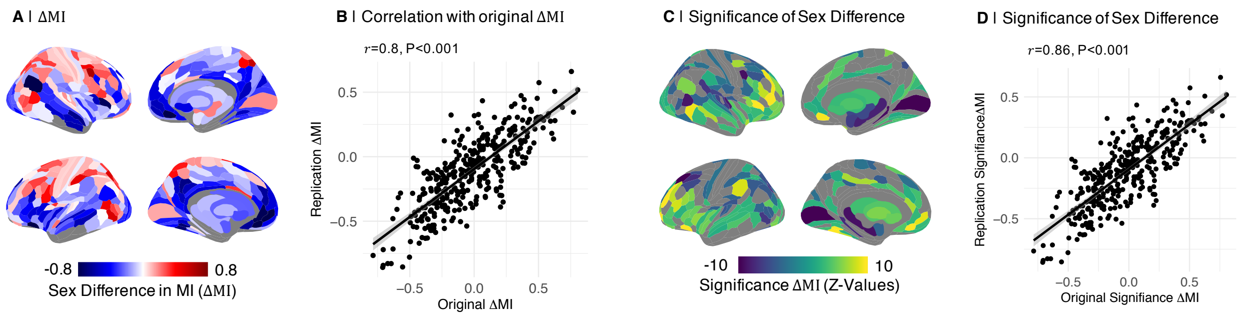

Fig. S31. Replication of key elements of Fig. 2 in the motion-matched sample:

(A) Sex difference in maturational index in the motion-matched sample (ΔMI). (B) The original and replication ΔMI map were significantly correlated ($r = 0.8, P < 2.2\text{e-}16$) (C) The sex difference in MI was significant in 229 regional nodes ($\alpha = 0.01$; $-2.57 < Z < 2.57$). (D) The significance map of sex differences in maturational index was significantly correlated with the original map ($r = 0.86, P < 2.2\text{e-}16$).

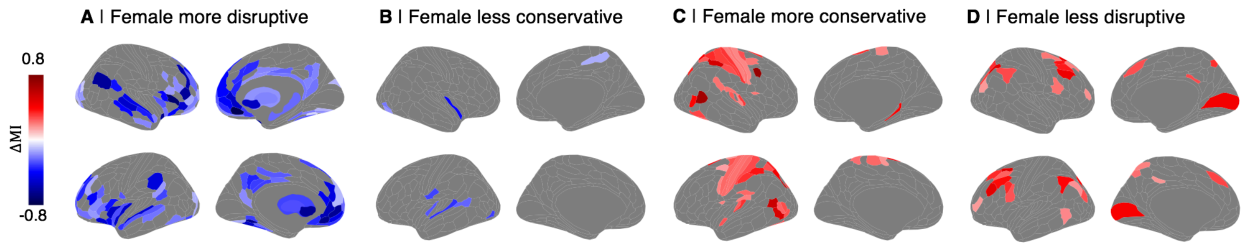

Fig. S32. Trends in the sex difference in maturational development (ΔMI) in the motion-matched sample:

Trends in ΔMI, thresholded for regions with significant sex differences ($P_{FDR} < 0.05$).

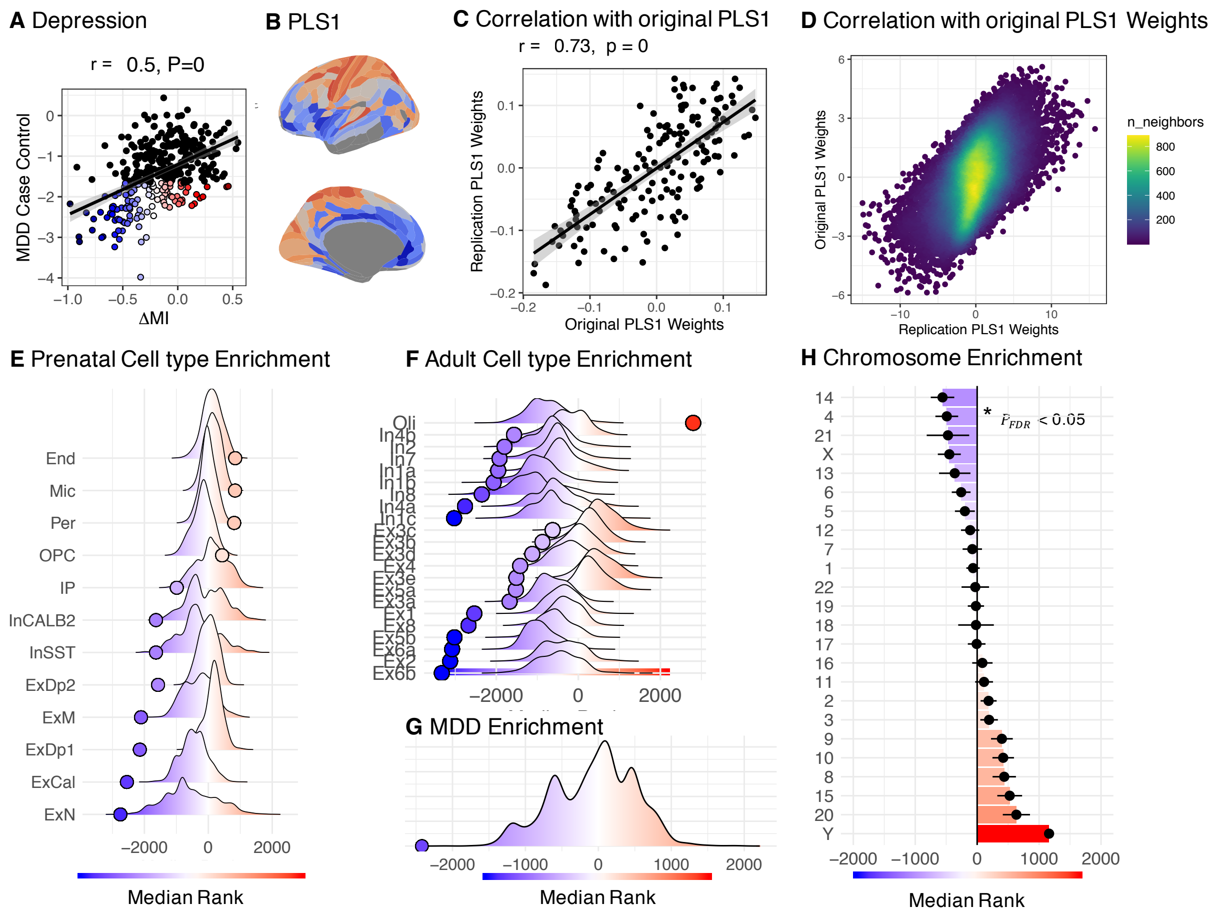

Fig. S33. Replication of key elements of Figs. 3 and 4 in the motion-matched sample:

(A) Correlation of motion-matched ΔMI with an MDD case-control map ($r = 0.5, P < 0.001, P_{spin} < 0.001$). Each point represents one of 346 cortical or subcortical regions; ROIs that show a significant MDD case-control difference, $t \neq 0$, and a significant sex difference in ΔMI, $t \neq 0$ are highlighted. We used partial least squares (PLS) regression to map the Allen Human Brain Atlas (AHBA) gene expression data (*49*) onto the ΔMI map. (B) PLS1 component. (C) The motion-matched PLS1 was significantly correlated ($r = 0.73, P < 0.001$) with the original PLS1. (D) Correlation of motion-matched PLS1 weights with the original PLS1 weights. (E) Enrichment analysis for prenatal cell type-specific genes. Negatively weighted genes (blue) were significantly enriched for genes expressed by prenatal somatostatin interneurons (InSST) and excitatory neurons and positively weighted genes were enriched for microglia and endothelial cells. (F) Enrichment analysis for adult cell type-specific genes. Negatively weighted genes were significantly enriched for genes expressed by adult inhibitory and excitatory neurons. (G) Enrichment analysis for MDD-related genes. Negatively weighted genes were significantly enriched for genes associated with major depressive disorder by an independent genome wide association study (*57*). (H) Enrichment analysis for chromosomal genes. Plot of the median rank of genes from each chromosome on PLS1, with standard deviations.

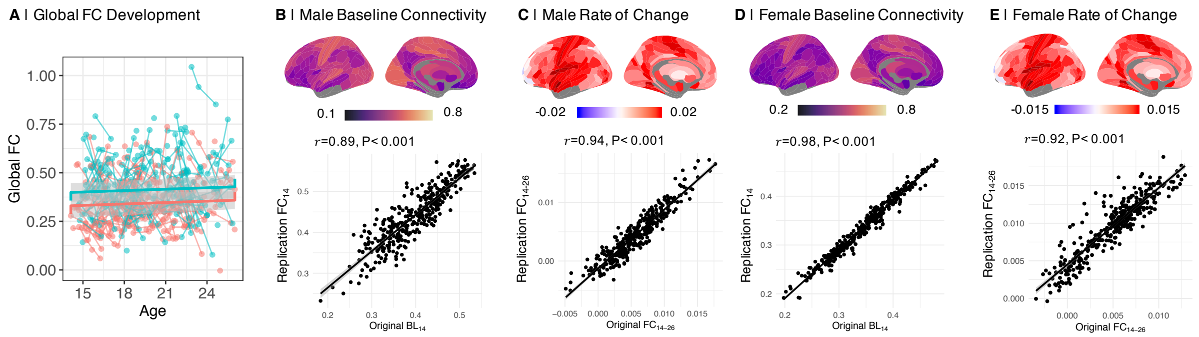

Fig. S34. Replication of key elements of Fig. 1 in edge-wise ICV-corrected sensitivity analysis:

Sex and age effects on functional connectivity (FC) were modeled using linear mixed effects models on different spatial scales. (A) Global FC increased with age ($t(217) = 2.43, P < 0.05$) and was higher in males ($t(296) = 3.96, P < 0.01$). (B)-(E) baseline connectivity at age 14 (FC_{14}) and adolescent rate of change (FC_{14-26}) in males and females were qualitatively and quantitatively highly consistent with the results of principal analysis (uncorrected for ICV).

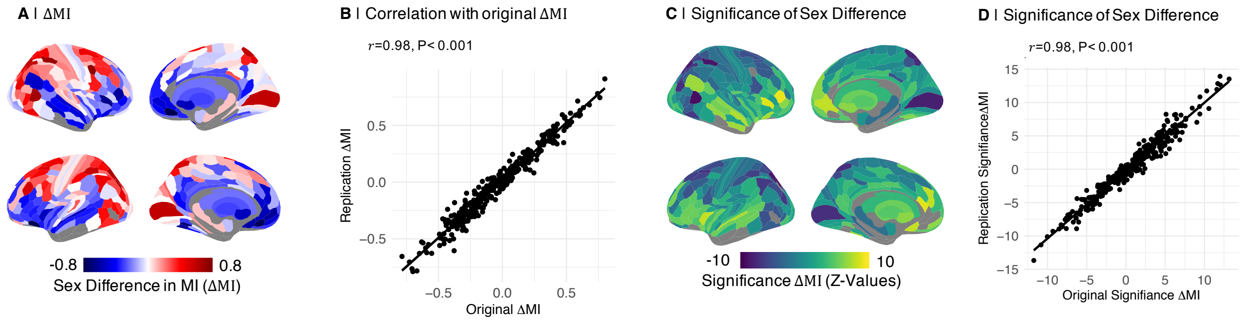

Fig. S35. Replication of key elements of Fig. 2 in edge-wise ICV-corrected sensitivity analysis:

(A) Sex difference in maturational index controlled for intracranial cortical volume (ICV): (ΔMI). (B) The original and replication ΔMI map were significantly correlated (r = 0.98, P < *0.001*) (C) The sex difference in MI was significant in 202 regional nodes (α = 0.01; -2.57 < Z < 2.57). (D) The significance map of sex differences in maturational index was significantly correlated with the original map (r = 0.98, P < *0.001*).

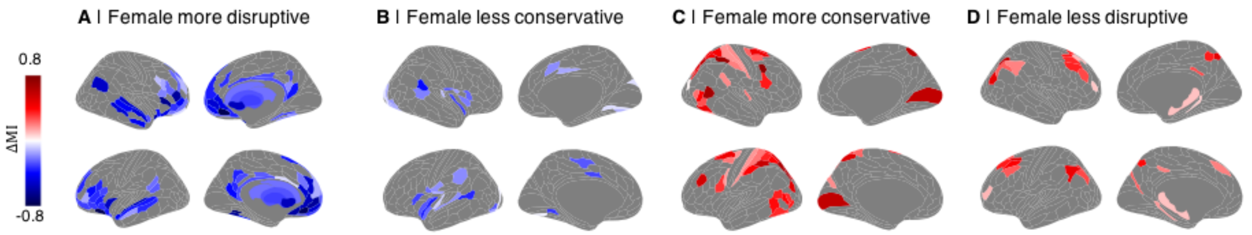

Fig. S36. Trends in the sex difference in maturational development (ΔMI) in the ICV-corrected sample:

Trends in ΔMI, thresholded for regions with significant sex differences ($P_{FDR} < 0.05$).

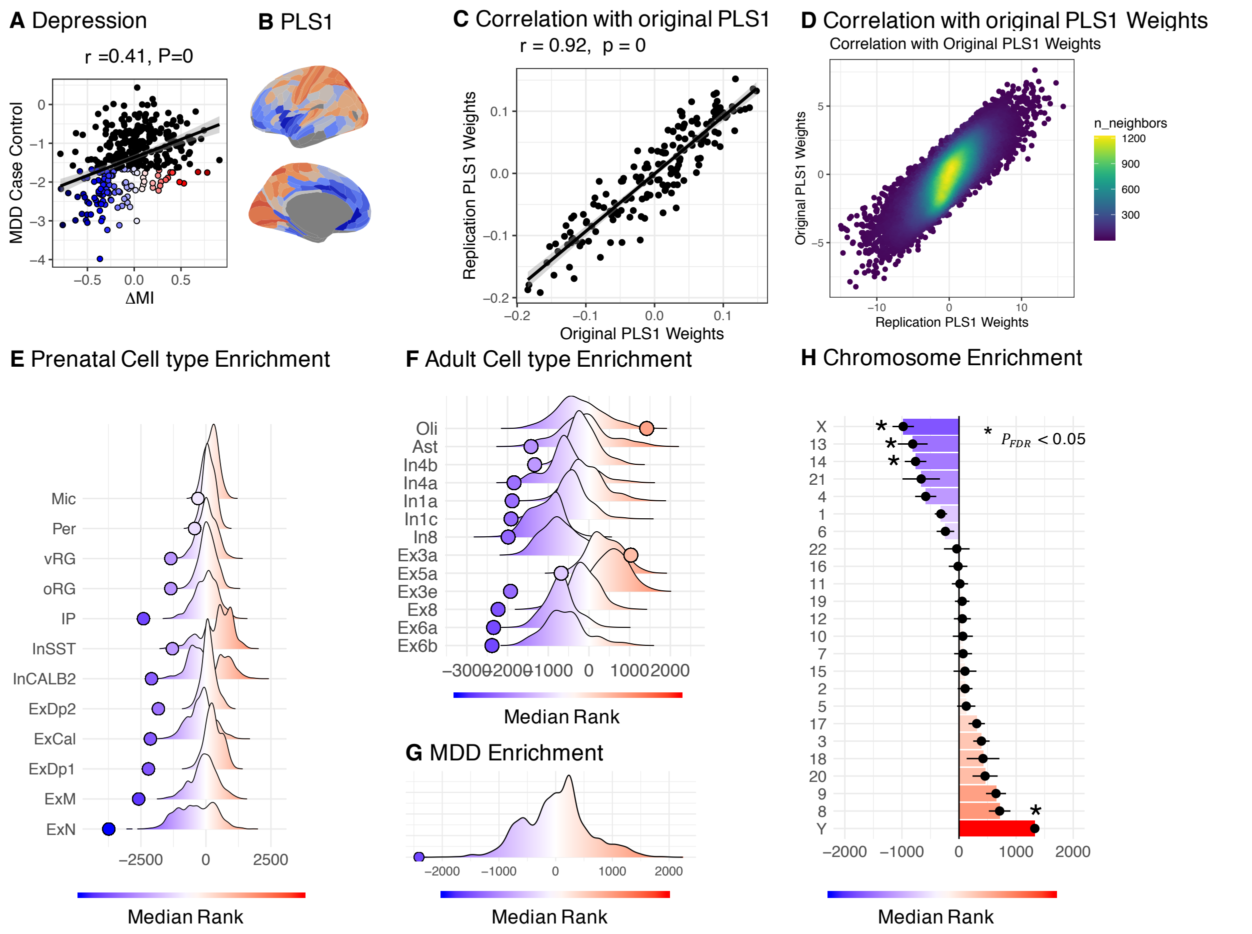

Fig. S37. Replication of key elements of Figs. 3 and 4 in edge-wise ICV-corrected sensitivity analysis:

(A) Correlation of edge-wise ICV-corrected ΔMI with a MDD case-control map ($r = 0.41, P < 0.01, P_{spin} < 0.001$). We used partial least squares (PLS) regression to map the Allen Human Brain Atlas (AHBA) gene expression data (*49*) onto the ΔMI map. (B) PLS1 component. (C) Correlation of ICV-controlled PLS1 with the original PLS1. (D) Correlation of ICV-controlled PLS1 weights with the original PLS1 weights. (E) Enrichment analysis for prenatal cell type-specific genes. Negatively weighted genes (blue) were significantly enriched for genes expressed by prenatal radial glia (vRG, oRG), microglia (Mic), somatostatin interneurons (InSST) and excitatory neurons. (F) Enrichment analysis for adult cell type-specific genes. Negatively weighted genes were significantly enriched for genes expressed by adult astrocytes, and various inhibitory and excitatory neurons. Positively weighted genes were enriched for oligodendrocytes. (G) Enrichment analysis for MDD-related genes. Negatively weighted genes were significantly enriched for genes associated with major depressive disorder by an independent genome wide association study (*57*). (H) Enrichment analysis for chromosomal genes. Plot of the median rank of genes from each chromosome on PLS1, with standard deviations.

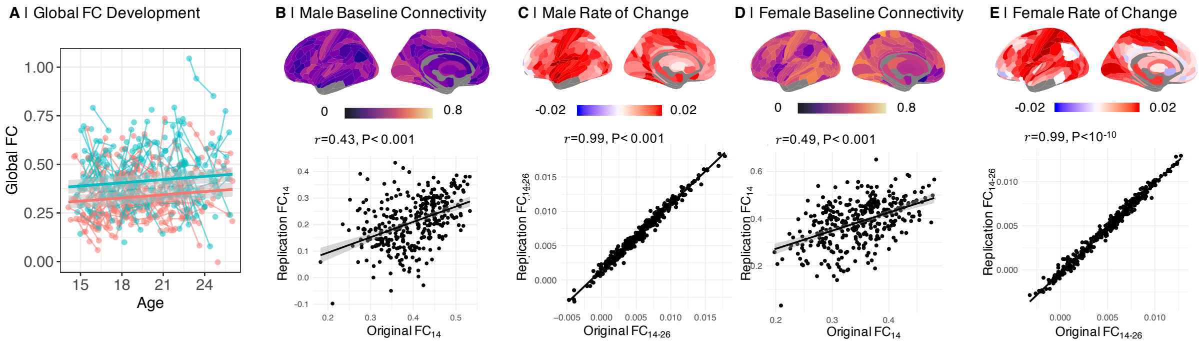

Fig. S38. Replication of key elements of Fig. 1 in global FC-corrected sensitivity analysis:

Sex and age effects on functional connectivity (FC) were modeled using linear mixed effects models on different spatial scales. (A) Global FC increased with age ($t(217) = 2.43, P < 0.05$) and was higher in males ($t(296) = 3.96, P < 0.01$). (B)-(E) baseline connectivity at age 14 (FC_{14}) and adolescent rate of change (FC_{14-26}) in males and females were qualitatively and quantitatively highly consistent with our main results.

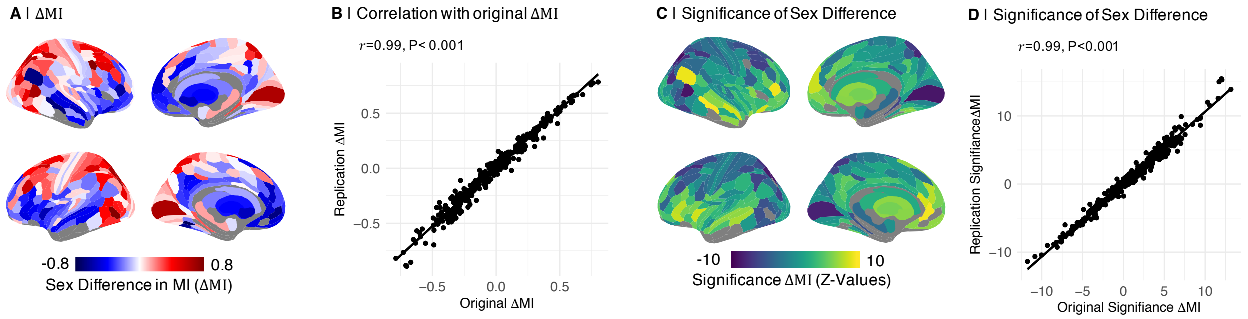

Fig. S39. Replication of key elements of Fig. 2 in global FC-corrected sensitivity analysis:

(A) Sex difference in maturational index controlled for global functional connectivity (FC): (ΔMI). (B) The original and replication ΔMI map were significantly correlated (r = 0.99, $P < 0.001$) (C) The sex difference in MI was significant in 202 regional nodes (α = 0.01; -2.57 < Z < 2.57). (D) The significance map of sex differences in maturational index was significantly correlated with the original map (r = 0.99, $P < 0.001$).

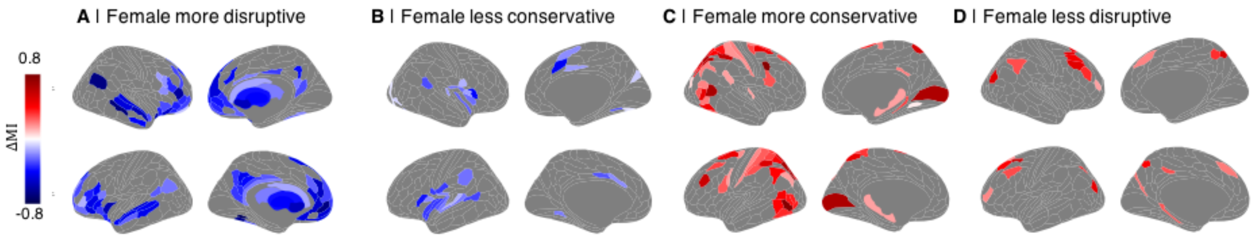

Fig. S40. Trends in the sex difference in maturational development (ΔMI) in global FC-corrected sensitivity analysis:

Trends in ΔMI, thresholded for regions with significant sex differences ($P_{FDR} < 0.05$).

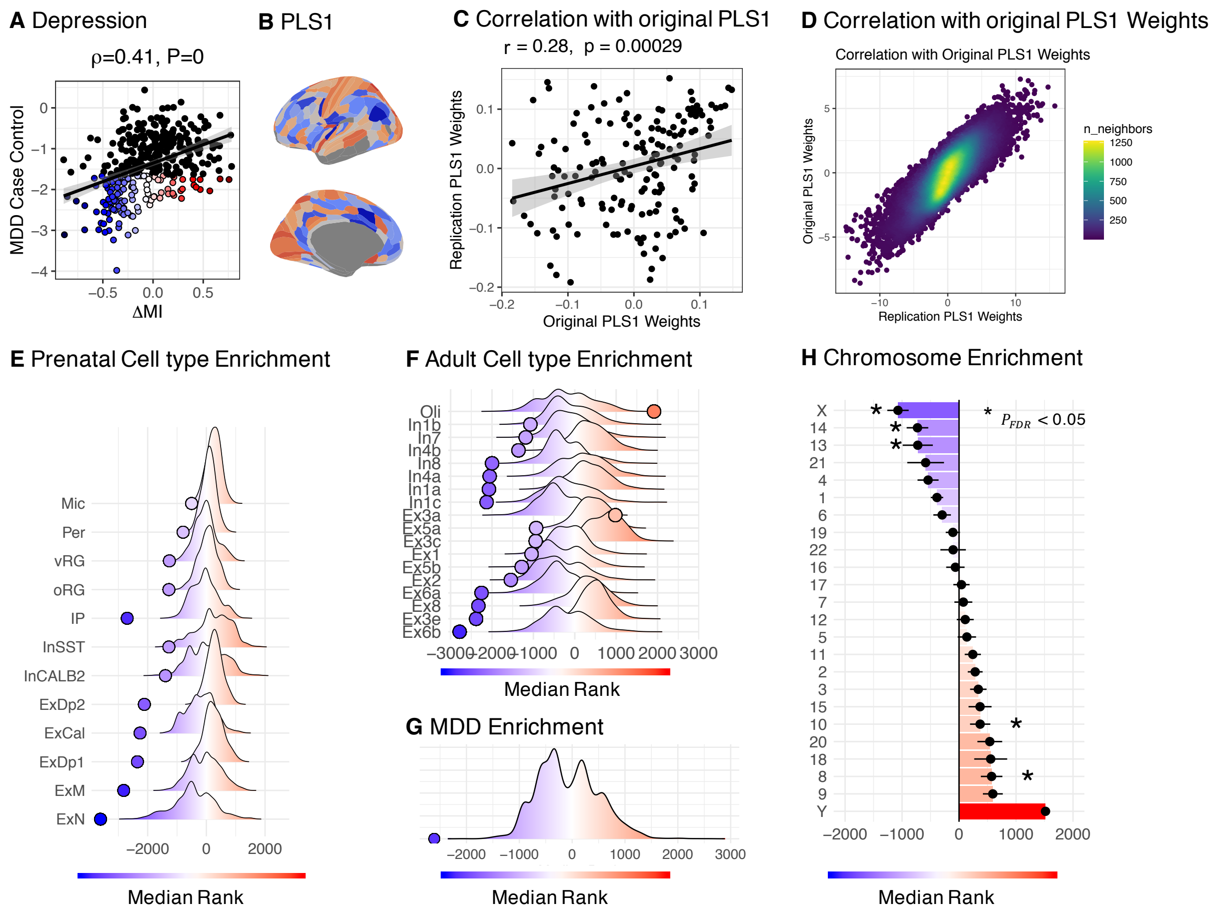

Fig. S41. Replication of key elements of Figs. 3 and 4 in global FC-corrected sensitivity analysis:

(A) Correlation of global-FC-controlled ΔMI with an MDD-case-control map ($r = 0.39, P < 0.01, P_{spin} < 0.001$). We used partial least squares (PLS) regression to map the Allen Human Brain Atlas (AHBA) gene expression data (*49*) onto the ΔMI map. (B) PLS1 component. (C) Correlation of global-FC-controlled PLS1 with the original PLS1. (D) Correlation of global-FC-controlled PLS1 weights with the original PLS1 weights. (E) Enrichment analysis for prenatal cell type-specific genes. Negatively weighted genes (blue) were significantly enriched for genes expressed by prenatal radial glia (vRG, oRG), microglia (Mic), somatostatin interneurons (InSST) and excitatory neurons. (F) Enrichment analysis for adult cell type-specific genes. Negatively weighted genes were significantly enriched for genes expressed by adult inhibitory and excitatory neurons. Positively weighted genes were enriched for oligodendrocytes. (G) Enrichment analysis for MDD-related genes. Negatively weighted genes were significantly enriched for genes associated with major depressive disorder by an independent genome wide association study (*57*). (H) Enrichment analysis

for chromosomal genes. Plot of the median rank of genes from each chromosome on PLS1, with standard deviations.

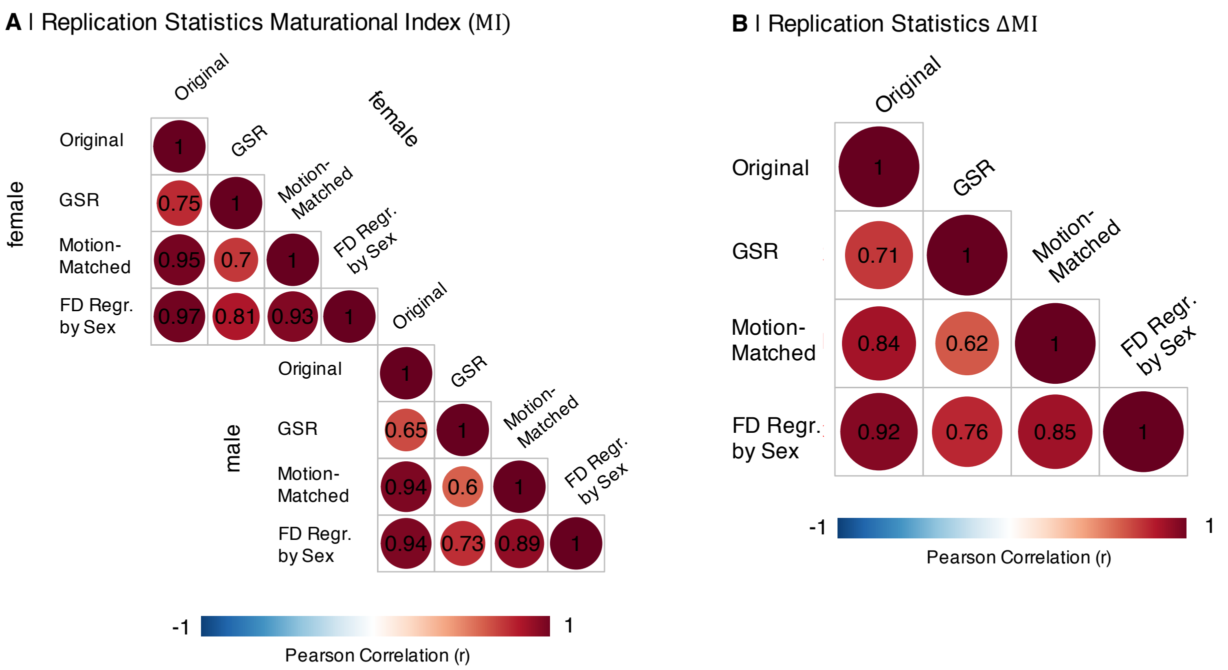

Fig. S42. Sex Difference in Maturational Index in Motion Sensitivity Analyses:

Correlation of maps estimated using the original dataset and the three sensitivity analyses to correct for motion. (A) Correlation of individual male and female maturational index (MI) maps. (B) Correlation of sex difference in maturational index (ΔMI) maps across all regions calculated from original and replication samples in males.

Diagnostic Specificity

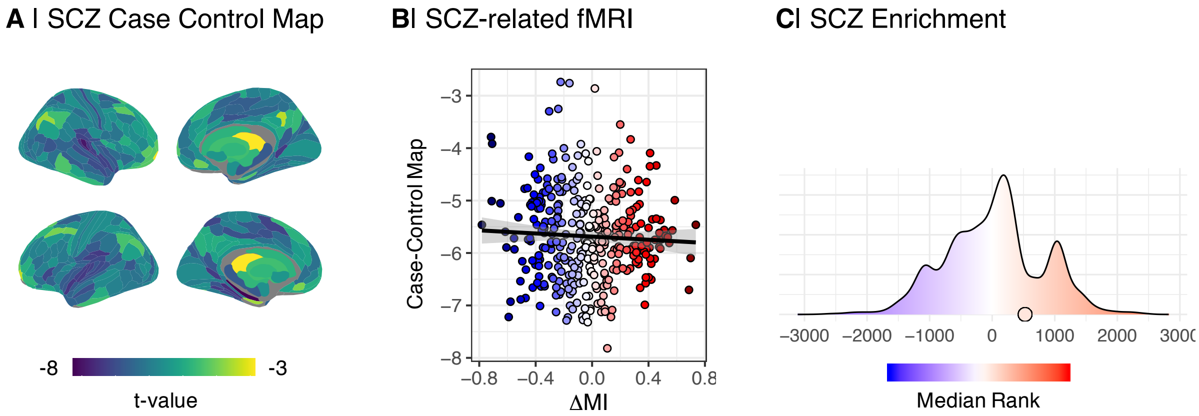

Fig. S43. Specificity analysis of the relationships between ΔMI, functional dysconnectivity in schizophrenia, and brain expression of risk genes for schizophrenia.

We tested the specificity of our results to major depression by repeating key analyses using comparable data from independent studies of schizophrenia. (A) We constructed a map of case-control differences in functional connectivity, using rsfMRI data from healthy controls (N=81) and schizophrenia cases (N=67) from a prior study (*41*, *42*). We estimated the case-control effect on regional functional connectivity strength using linear mixed effects models, with a random effect for subject ID and fixed effects of group, age, sex, and mean framewise displacement. The *t*-statistics for case-control difference in regional weighted degree are mapped on the cortical surface; $t < 0$ indicates areas of reduced degree in schizophrenia. (B) Scatterplot of schizophrenia case-control t-statistics (y-axis) versus ΔMI (x-axis). Each point represents one of 346 cortical or subcortical regions. There was no significant correlation ($r = 0.05, P = 0.35, P_{spin} = 0.47$) between the spatial map of schizophrenia case-control differences in weighted degree, t, and the spatial map of ΔMI. (C) Analysis for enrichment of 130 schizophrenia-related genes in the list of genes transcriptionally co-located with the ΔMI map and strongly weighted on the first PLS component (PLS1). The histogram shows the distribution of the median rank of 183 genes randomly sampled under the null hypothesis and the observed median rank of the 183 genes associated with schizophrenia (*43*) is indicated by an open circle on the x-axis. There was no evidence that genes transcriptionally co-located with ΔMI were significantly enriched for these schizophrenia-related genes.

Contextualization

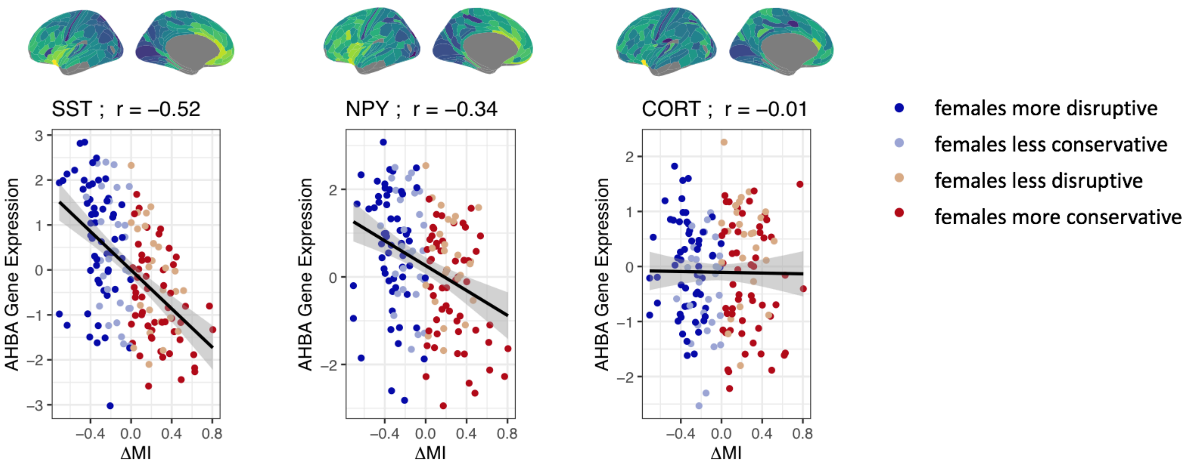

Fig. S44. Illustrative correlations between ΔMI and three genes (*SST*, *NPY* and *CORT*):

SST, NPY and *CORT* are three genes that were previously highlighted by an independent study of gene expression and MRI phenotypes of MDD (*70*). We noted that two of these genes (*SST* and *NPY*) were also included in the list of genes with significantly non-zero weights on PLS1 ($|Z| > 2.58$). The *Z*-scored PLS1 weights for each gene were as follows: *SST* ($Z_{FDR} = -10.05$), NPY ($Z_{FDR} = -4.63$), and *CORT* ($Z_{FDR} = -0.25$). To highlight the partial convergence of significant results for *SST* and *NPY* between a prior study (*70*) and the current study, and to illustrate how PLS1 weights represent the variable strength of covariation between ΔMI and individual gene expression, these three scatterplots show expression of each gene at each region of the Allen Human Brain Atlas (AHBA; y-axis) versus ΔMI of each region (x-axis) with each region colour-coded according to its sexually divergent developmental profile. Regions with more disruptive development in females (ΔMI < 0; dark blue points) have increased expression of *SST* and *NPY*, compared to regions with more conservative development in females (ΔMI > 0; dark red points). Whereas expression of *CORT* is not correlated with regional variation in ΔMI. The small brain maps above each scatterplot represent the anatomical expression of each gene in the AHBA dataset.

Methodological Considerations

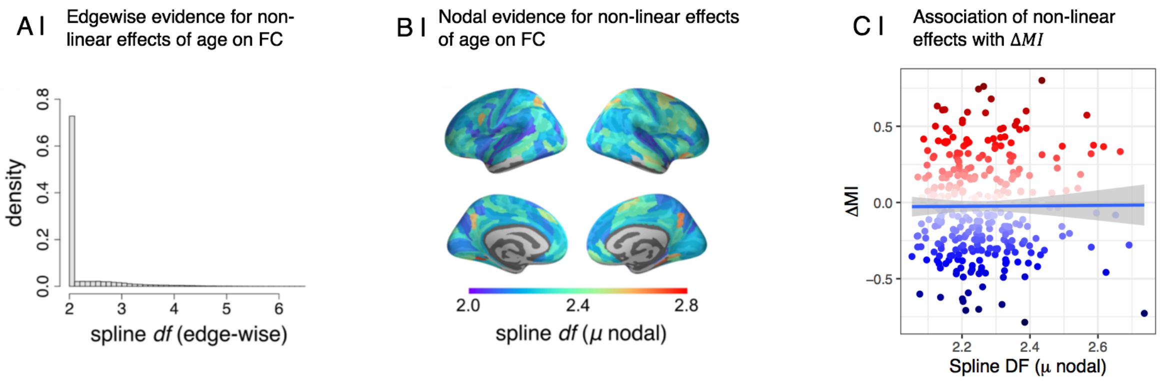

Fig. S45. Non-linear Effects of Age on FC:

In previous work on this sample (*6*), locally adaptive mixed effect smoothing splines were fitted to edgewise trajectories of functional connectivity development to inspect potential non-linear effects of age on FC. In these models, non-linear trajectories will be best fitted by spline functions with degrees of freedom greater than 2, whereas linear trajectories will be best fitted by spline functions with 2 df, analogous to the intercept and gradient parameters of a simple linear model. (A) Distribution of effective df of smoothing splines across edges: 71.7% trajectories had df 2.1, suggesting that most trajectories are linear. (B) Cortical distribution of average nodal df (averaged across all of a node's edges). (C) Lastly, we correlated the cortical map of sex differences in maturational index, (ΔMI), with the cortical map of average nodal df. We show that there is no association between evidence for non-linearities in functional connectivity development with age, as indicated by an effective df > 2 ($r = 0.006, P = 0.9$). Panels A and B are reproduced, with permission, from the Supplementary Information for (*6*).

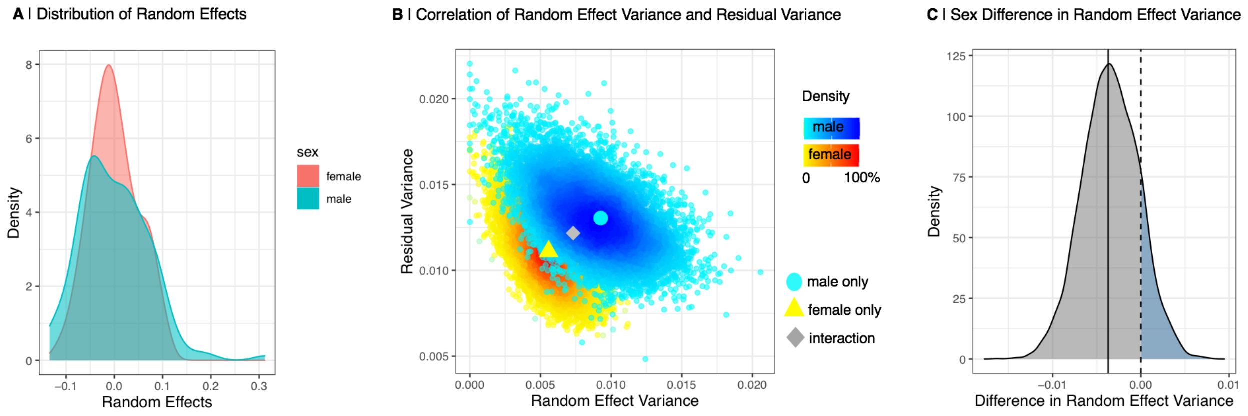

Fig. S46. Random effect variance:

We estimated the random effects of sex on global functional connectivity in a sex stratified approach. we employed a bootstrap approach, resampling subjects with replacement 10,000 times within each age bin, thus creating a distribution of random effect and residual variance. (A) Distribution of random effects estimated in a sex stratified approach. (B) Correlation of random effect variance and residual variance for males and females estimated in a bootstrap approach. The real male and female, as well as the random effect variance of an interaction model are marked. This plot shows a clear separation of male and female random effect variance. (C) We estimated whether the random effect variance of males and females was equal. In a permutation approach, we estimated whether the difference in random effect variance between males and females significantly differed from 0. We find a P-value of $P = 0.14$.

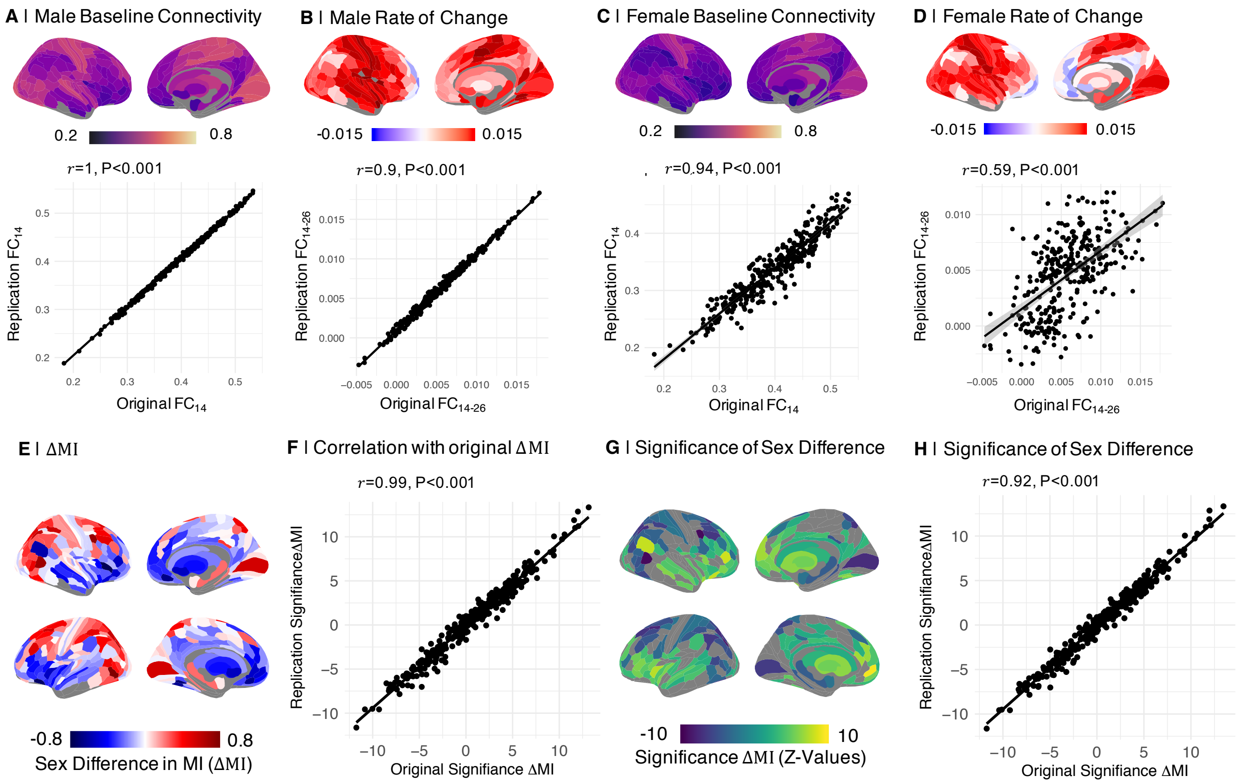

Fig. S47. Age x Sex Interaction Model:

We modelled functional connectivity maturational in a joint model for males and females and included an interaction term for the interaction of age and sex. (A) (B)-(E) From this model, we derived updated FC_{14} and FC_{14-26} for males and females. We find that those updated measures are highly correlated with our original measures from the sex stratified approach. (F)-(G) We further find that the sex difference in maturational index, MI, as well as the map of the effect size of this sex difference (Z-values), are highly correlated with our main analysis.

Tables

Data

Table S1 NSPN sample overview:

A total of N = 298 healthy young people participated in an accelerated longitudinal MRI study. The recruitment was balanced for sex in each of five age-defined strata. Subjects were scanned between 1 and 3 times with scans taking place at baseline, 6 and 18 months later. FD = framewise displacement, a measure of head movement in mm, was significantly greater in males compared to females on average over all ages, and in the youngest two age strata specifically ($P < 0.05$, uncorrected).

Sex	#Scans	#Scanned			At Baseline				# Subj./Agebin				
		1	2	3	μAge	σAge	μFD	σFD	1	2	3	4	5
female	259	54	86	11	19.8	2.9	0.11	0.05	34	39	24	32	22
male	261	41	98	8	19.2	3.8	0.13	0.05	32	33	24	35	23

Analysis of Effects on Parameters of Adolescent Brain Development

Table S2. Age Effects on FC per Sex

	female	male
uncorrected all	93	83
fdr-corrected all	0	12
uncorrected cortico-cortical	101	87
fdr-corrected cortico-cortical	0	12
uncorrected cortico-subcortical	10	13
fdr-corrected cortico-subccortical	0	0
uncorrected thalamus	14	4
fdr-corrected thalamus	5	26
uncorrected caudate	26	11
fdr-corrected caudate	10	1
uncorrected putamen	27	30
fdr-corrected putamen	61	71
uncorrected pallidum	20	61
fdr-corrected pallidum	15	35
uncorrected hippocampus	0	0
fdr-corrected hippocampus	0	0
uncorrected amygdala	0	0
fdr-corrected amygdala	0	0
uncorrected accumbens	0	0
fdr-corrected accumbens	0	0
uncorrected diencephalon	0	0
fdr-corrected diencephalon	0	0

Table S3. ROIs with significantly different ΔMI:

We tested for sex differences in maturational index (MI). Here we show all 230 regions displaying a significant sex difference in MI ($P(\Delta\text{MI} = 0) < 0.05$). We show the regions name in the HCP parcellation; it's ΔMI value, the p-value and Z-value from the parametric test of the sex difference in MI; the functional network they are located in ('Yeo-Network'); as well as which one of four trends they display: (1) 'female more conservative', (2) 'female more disruptive', (3) 'female less conservative', (4) 'female less disruptive' (cf. Supplementary Fig. S14).

ROI	**ΔMI**	Z	P	Trend	Yeo-Network
R FOP5	-0.79	10.57	0.00	female more disruptive	Ventral Attention
R s32	-0.71	11.70	0.00	female more disruptive	Default Mode
L s32	-0.71	12.05	0.00	female more disruptive	Default Mode
L VMV2	-0.71	11.59	0.00	female more disruptive	Visual
L 47s	-0.67	12.00	0.00	female more disruptive	Default Mode
R PGi	-0.64	9.04	0.00	female more disruptive	Default Mode
L MI	-0.63	9.42	0.00	female more disruptive	Ventral Attention
L VMV3	-0.63	13.16	0.00	female more disruptive	Visual
R IFSa	-0.60	9.39	0.00	female more disruptive	Frontoparietal
R Pir	-0.59	6.98	0.00	female more disruptive	Subcortex
L accumbens	-0.57	6.79	0.00	female more disruptive	Subcortex
L p32	-0.56	10.30	0.00	female more disruptive	Default Mode
L a24	-0.50	9.44	0.00	female more disruptive	Default Mode
R STSvp	-0.50	7.07	0.00	female more disruptive	Default Mode
R STSda	-0.49	7.62	0.00	female more disruptive	Default Mode
R accumbens	-0.49	5.13	0.00	female more disruptive	Subcortex
R A5	-0.48	5.98	0.00	female more disruptive	Somatomotor
L A5	-0.47	6.47	0.00	female more disruptive	Somatomotor
R 47l	-0.47	5.54	0.00	female more disruptive	Default Mode
L p24	-0.46	8.45	0.00	female more disruptive	Default Mode
L 10r	-0.46	6.29	0.00	female more disruptive	Default Mode
R 47s	-0.45	7.63	0.00	female more disruptive	Default Mode
R FOP4	-0.45	6.86	0.00	female more disruptive	Ventral Attention
R a32pr	-0.44	5.85	0.00	female more disruptive	Frontoparietal
L PoI1	-0.44	6.61	0.00	female less conservative	Ventral Attention
R AAIC	-0.44	5.37	0.00	female more disruptive	Default Mode

ROI	**ΔMI**	Z	P	Trend	Yeo-Network
L 45	-0.43	7.17	0.00	female more disruptive	Default Mode
L 31pd	-0.43	4.97	0.00	female more disruptive	Default Mode
R STSdp	-0.43	5.61	0.00	female more disruptive	Default Mode
R p24	-0.42	7.54	0.00	female more disruptive	Default Mode
L FOP4	-0.41	6.32	0.00	female more disruptive	Ventral Attention
L FOP5	-0.41	5.78	0.00	female more disruptive	Ventral Attention
L d32	-0.40	6.34	0.00	female more disruptive	Default Mode
R a24	-0.40	7.02	0.00	female more disruptive	Default Mode
L SFL	-0.40	5.93	0.00	female more disruptive	Frontoparietal
L IFSa	-0.40	5.10	0.00	female more disruptive	Frontoparietal
L STSda	-0.40	5.90	0.00	female more disruptive	Default Mode
L AAIC	-0.39	6.20	0.00	female more disruptive	Default Mode
R p32pr	-0.38	5.08	0.00	female more disruptive	Ventral Attention
L 8BL	-0.38	5.32	0.00	female more disruptive	Default Mode
R p32	-0.38	6.19	0.00	female more disruptive	Default Mode
L Pir	-0.38	3.81	0.00	female more disruptive	Subcortex
L 25	-0.38	5.11	0.00	female more disruptive	Limbic
L pallidum	-0.37	5.35	0.00	female more disruptive	Subcortex
R 9m	-0.36	6.81	0.00	female more disruptive	Default Mode
L 9p	-0.36	5.32	0.00	female more disruptive	Default Mode
L STGa	-0.36	4.59	0.00	female more disruptive	Default Mode
R 8BL	-0.36	3.76	0.00	female more disruptive	Default Mode
R POS1	-0.35	4.39	0.00	female more disruptive	Default Mode
L IFSp	-0.35	5.05	0.00	female more disruptive	Frontoparietal
L 47l	-0.35	6.28	0.00	female more disruptive	Default Mode
L AVI	-0.34	4.25	0.00	female more disruptive	Frontoparietal
L STSdp	-0.34	5.28	0.00	female more disruptive	Default Mode
R 10r	-0.34	4.50	0.00	female more disruptive	Default Mode
L STSvp	-0.34	5.94	0.00	female more disruptive	Default Mode
L putamen	-0.34	6.28	0.00	female more disruptive	Subcortex
L POS1	-0.33	3.94	0.00	female more disruptive	Default Mode
L PoI2	-0.33	4.84	0.00	female less conservative	Ventral Attention
R VMV2	-0.33	6.42	0.00	female less conservative	Visual
L 31pv	-0.33	3.30	0.00	female more disruptive	Default Mode
L 7m	-0.32	3.98	0.00	female more disruptive	Default Mode

ROI	**ΔMI**	Z	P	Trend	Yeo-Network
L 10v	-0.32	5.02	0.00	female more disruptive	Limbic
R 46	-0.32	4.93	0.00	female more disruptive	Frontoparietal
L v23ab	-0.31	4.14	0.00	female more disruptive	Default Mode
L VMV1	-0.31	5.16	0.00	female less conservative	Visual
R 25	-0.31	4.42	0.00	female more disruptive	Limbic
R 9p	-0.31	3.09	0.00	female more disruptive	Default Mode
R STV	-0.31	4.27	0.00	female less conservative	Default Mode
L 43	-0.30	4.87	0.00	female less conservative	Somatomotor
R TE1a	-0.30	5.33	0.00	female more disruptive	Default Mode
R PoI1	-0.30	4.72	0.00	female less conservative	Ventral Attention
L OP4	-0.30	4.45	0.00	female less conservative	Somatomotor
R p47r	-0.29	4.87	0.00	female more disruptive	Frontoparietal
L VVC	-0.29	5.25	0.00	female more disruptive	Visual
R 23d	-0.29	4.01	0.00	female more disruptive	Default Mode
R MI	-0.29	4.74	0.00	female more disruptive	Ventral Attention
R PHA2	-0.29	2.47	0.02	female more disruptive	Visual
L 52	-0.29	3.54	0.00	female less conservative	Somatomotor
R IFSp	-0.29	4.16	0.00	female more disruptive	Frontoparietal
R 31pd	-0.29	3.57	0.00	female more disruptive	Default Mode
R pallidum	-0.28	4.42	0.00	female more disruptive	Subcortex
L 9-46d	-0.28	4.79	0.00	female more disruptive	Frontoparietal
R 10d	-0.28	4.24	0.00	female more disruptive	Default Mode
L TPOJ1	-0.27	3.99	0.00	female less conservative	Ventral Attention
L PF	-0.26	3.52	0.00	female more disruptive	Ventral Attention
R AVI	-0.25	2.90	0.01	female more disruptive	Frontoparietal
L STSva	-0.25	4.51	0.00	female more disruptive	Default Mode
L p24pr	-0.25	4.04	0.00	female less conservative	Ventral Attention
R RSC	-0.25	2.60	0.02	female more disruptive	Default Mode
R 10v	-0.25	3.24	0.00	female more disruptive	Limbic
R VMV3	-0.24	7.13	0.00	female more disruptive	Visual
R STGa	-0.24	3.65	0.00	female more disruptive	Default Mode
L 23d	-0.24	3.65	0.00	female more disruptive	Default Mode
L a32pr	-0.24	3.25	0.00	female more disruptive	Frontoparietal
L RSC	-0.24	2.87	0.01	female more disruptive	Default Mode
L 24dd	-0.23	2.57	0.02	female less conservative	Somatomotor

ROI	**ΔMI**	Z	P	Trend	Yeo-Network
R 33pr	-0.23	3.22	0.00	female more disruptive	Ventral Attention
R STSva	-0.23	3.33	0.00	female more disruptive	Default Mode
R VVC	-0.23	4.14	0.00	female more disruptive	Visual
L STV	-0.23	3.54	0.00	female more disruptive	Default Mode
R OP2-3	-0.23	4.41	0.00	female less conservative	Somatomotor
L a47r	-0.23	3.93	0.00	female more disruptive	Frontoparietal
L a10p	-0.22	4.17	0.00	female more disruptive	Frontoparietal
L 10d	-0.22	3.86	0.00	female more disruptive	Default Mode
L MBelt	-0.22	3.54	0.00	female less conservative	Somatomotor
L A4	-0.22	4.45	0.00	female less conservative	Somatomotor
R putamen	-0.22	4.63	0.00	female more disruptive	Subcortex
L thalamus	-0.22	3.16	0.00	female more disruptive	Subcortex
R 47m	-0.21	2.78	0.01	female more disruptive	Default Mode
R a47r	-0.21	4.18	0.00	female more disruptive	Frontoparietal
L PGi	-0.21	2.17	0.05	female more disruptive	Default Mode
R v23ab	-0.21	2.79	0.01	female more disruptive	Default Mode
R FOP2	-0.19	3.39	0.00	female less conservative	Somatomotor
L 44	-0.19	3.25	0.00	female more disruptive	Frontoparietal
L p10p	-0.18	3.87	0.00	female more disruptive	Frontoparietal
R 44	-0.18	2.82	0.01	female more disruptive	Frontoparietal
R p24pr	-0.18	3.27	0.00	female less conservative	Ventral Attention
L OP2-3	-0.17	2.90	0.01	female less conservative	Somatomotor
L 9m	-0.17	4.29	0.00	female more disruptive	Default Mode
L a24pr	-0.17	2.66	0.01	female less conservative	Ventral Attention
R V8	-0.16	4.44	0.00	female less conservative	Visual
R SCEF	-0.16	2.42	0.02	female less conservative	Ventral Attention
R IFJa	-0.16	2.34	0.03	female more disruptive	Frontoparietal
L caudate	-0.15	2.31	0.03	female more disruptive	Subcortex
L 23c	-0.15	2.29	0.03	female less conservative	Ventral Attention
R IFJp	-0.15	2.54	0.02	female more disruptive	Dorsal Attention
L LO2	-0.14	2.30	0.03	female less conservative	Visual
L FOP3	-0.14	2.38	0.03	female less conservative	Ventral Attention
R A1	-0.13	2.43	0.02	female less conservative	Somatomotor
R 9-46d	-0.12	2.15	0.05	female more disruptive	Frontoparietal
R 7m	-0.12	2.24	0.04	female more disruptive	Default Mode

ROI	**ΔMI**	Z	P	Trend	Yeo-Network
R V6	-0.11	2.73	0.01	female less conservative	Visual
R LO2	-0.10	3.42	0.00	female less conservative	Visual
L PBelt	-0.09	2.21	0.04	female less conservative	Somatomotor
R 11l	-0.09	2.70	0.01	female more disruptive	Frontoparietal
R V4	-0.08	3.56	0.00	female less conservative	Visual
R VMV1	-0.06	3.41	0.00	female less conservative	Visual
L V4	-0.01	2.50	0.02	female less conservative	Visual
R V3CD	0.02	3.42	0.00	female more conservative	Visual
L hippocampus	0.09	-2.20	0.04	female less disruptive	Subcortex
R a9-46v	0.10	-2.46	0.02	female less disruptive	Frontoparietal
L FEF	0.15	-2.14	0.05	female more conservative	Dorsal Attention
L PreS	0.15	-3.29	0.00	female less disruptive	Visual
R 2	0.15	-2.35	0.03	female more conservative	Somatomotor
L 6v	0.16	-3.16	0.00	female more conservative	Somatomotor
L 1	0.16	-3.09	0.00	female more conservative	Somatomotor
R 1	0.16	-3.04	0.00	female more conservative	Somatomotor
R PFt	0.16	-2.30	0.03	female more conservative	Dorsal Attention
L 6d	0.17	-2.54	0.02	female more conservative	Somatomotor
R 52	0.17	-2.84	0.01	female more conservative	Somatomotor
L 31a	0.18	-3.39	0.00	female less disruptive	Frontoparietal
L PGs	0.18	-3.36	0.00	female less disruptive	Default Mode
R 8Ad	0.18	-3.62	0.00	female less disruptive	Default Mode
R TPOJ3	0.19	-2.21	0.04	female more conservative	Dorsal Attention
R 8BM	0.19	-2.23	0.04	female less disruptive	Frontoparietal
R PSL	0.19	-3.14	0.00	female more conservative	Default Mode
L a9-46v	0.19	-3.58	0.00	female less disruptive	Frontoparietal
L V6A	0.20	-2.60	0.02	female more conservative	Visual
R PGs	0.21	-3.24	0.00	female less disruptive	Default Mode
L s6-8	0.21	-3.06	0.00	female less disruptive	Frontoparietal
R FFC	0.21	-2.95	0.01	female more conservative	Visual
R FST	0.22	-3.82	0.00	female more conservative	Dorsal Attention
L 8BM	0.22	-2.66	0.01	female less disruptive	Frontoparietal
R PreS	0.23	-5.01	0.00	female less disruptive	Visual
R MT	0.23	-2.60	0.02	female more conservative	Visual
L 2	0.23	-2.48	0.02	female more conservative	Somatomotor

ROI	**ΔMI**	Z	P	Trend	Yeo-Network
R 6d	0.24	-3.44	0.00	female more conservative	Somatomotor
L 8C	0.27	-4.09	0.00	female less disruptive	Frontoparietal
R PFm	0.27	-3.22	0.00	female less disruptive	Frontoparietal
L 5L	0.28	-3.46	0.00	female more conservative	Somatomotor
R d23ab	0.28	-4.89	0.00	female less disruptive	Default Mode
R p9-46v	0.29	-2.83	0.01	female less disruptive	Frontoparietal
L 6r	0.29	-4.24	0.00	female more conservative	Ventral Attention
L 8Av	0.29	-4.25	0.00	female less disruptive	Frontoparietal
L 7PC	0.29	-3.00	0.00	female more conservative	Dorsal Attention
R 6r	0.30	-4.07	0.00	female more conservative	Ventral Attention
L PH	0.31	-3.70	0.00	female more conservative	Dorsal Attention
L 7AL	0.31	-2.80	0.01	female more conservative	Dorsal Attention
L IP1	0.32	-3.47	0.00	female less disruptive	Frontoparietal
R PCV	0.32	-4.11	0.00	female less disruptive	Default Mode
L MT	0.32	-2.67	0.01	female more conservative	Visual
L V4t	0.32	-3.46	0.00	female more conservative	Visual
R 6ma	0.34	-4.23	0.00	female more conservative	Ventral Attention
L MST	0.34	-4.28	0.00	female more conservative	Visual
R IP0	0.34	-4.71	0.00	female more conservative	Dorsal Attention
L 8Ad	0.34	-5.79	0.00	female less disruptive	Default Mode
R VIP	0.36	-4.71	0.00	female more conservative	Dorsal Attention
R V7	0.36	-3.02	0.00	female more conservative	Visual
L PFt	0.36	-4.11	0.00	female more conservative	Dorsal Attention
R 6a	0.37	-3.96	0.00	female more conservative	Dorsal Attention
L PHT	0.38	-4.75	0.00	female more conservative	Dorsal Attention
R LIPv	0.38	-5.10	0.00	female more conservative	Dorsal Attention
L LIPv	0.38	-4.90	0.00	female more conservative	Dorsal Attention
R 7PC	0.38	-3.75	0.00	female more conservative	Dorsal Attention
R s6-8	0.39	-4.74	0.00	female less disruptive	Frontoparietal
L 7Pm	0.39	-5.65	0.00	female less disruptive	Frontoparietal
L TPOJ2	0.39	-4.71	0.00	female more conservative	Dorsal Attention
R PH	0.39	-5.95	0.00	female more conservative	Dorsal Attention
R i6-8	0.40	-5.28	0.00	female less disruptive	Frontoparietal
L FFC	0.40	-6.81	0.00	female more conservative	Visual
R PGp	0.41	-6.37	0.00	female less disruptive	Dorsal Attention

ROI	**ΔMI**	Z	P	Trend	Yeo-Network
R 8Av	0.41	-5.67	0.00	female less disruptive	Frontoparietal
L 6a	0.42	-4.92	0.00	female more conservative	Dorsal Attention
L V7	0.42	-3.74	0.00	female more conservative	Visual
R MST	0.43	-4.44	0.00	female more conservative	Visual
L PGp	0.43	-5.79	0.00	female less disruptive	Dorsal Attention
L 7Am	0.44	-6.34	0.00	female less disruptive	Dorsal Attention
L 6ma	0.44	-5.45	0.00	female more conservative	Ventral Attention
L PFm	0.46	-7.15	0.00	female less disruptive	Frontoparietal
R 7Am	0.47	-6.02	0.00	female more conservative	Dorsal Attention
R 8C	0.48	-6.24	0.00	female less disruptive	Frontoparietal
L AIP	0.48	-4.87	0.00	female more conservative	Dorsal Attention
L 7PL	0.49	-6.72	0.00	female more conservative	Dorsal Attention
L VIP	0.49	-6.28	0.00	female more conservative	Dorsal Attention
R 7Pm	0.50	-7.75	0.00	female less disruptive	Frontoparietal
L i6-8	0.52	-7.12	0.00	female less disruptive	Frontoparietal
R IPS1	0.52	-7.42	0.00	female more conservative	Dorsal Attention
L p9-46v	0.54	-5.07	0.00	female more conservative	Frontoparietal
R 7PL	0.54	-7.60	0.00	female more conservative	Dorsal Attention
R AIP	0.57	-6.30	0.00	female more conservative	Dorsal Attention
R MIP	0.58	-8.11	0.00	female more conservative	Dorsal Attention
R IP1	0.59	-7.40	0.00	female less disruptive	Frontoparietal
R V1	0.60	-8.27	0.00	female more conservative	Visual
L V1	0.62	-7.60	0.00	female more conservative	Visual
L MIP	0.63	-8.23	0.00	female more conservative	Dorsal Attention
L IPS1	0.63	-8.53	0.00	female more conservative	Dorsal Attention
R TPOJ2	0.68	-9.32	0.00	female more conservative	Dorsal Attention
R PEF	0.76	-10.03	0.00	female more conservative	Dorsal Attention
L IP0	0.77	-10.83	0.00	female more conservative	Dorsal Attention
L FST	0.80	-11.75	0.00	female more conservative	Dorsal Attention

Enrichment Analysis

Table S4. Chromosomal Enrichment Null Model Statistics:

We assessed chromosomal enrichment of the genes on PLS1 to test our hypothesis that sex chromosomal gene expression is related to the sexual differences in adolescent brain development. We used a median gene rank approach to analyse an enrichment of PLS1 for genes located on specific chromosomes. We did so by matching for gene length. Specifically, we resampled genes from the Allen Human Brain Atlas (AHBA) until we found a subset of genes that were not significantly different in length ($P < 0.05$) from the empirical gene list. Here, we show the results from the gene length matching algorithm, including the P-, s- and Z-value for the test of difference in gene length between the empirical gene list and the subset of resampled genes, the number of genes N Genes, to resample from, as well as which approach the algorithm converged on (see Supplementary Text "Matching for Gene Length" for details.)

Chromosome	P	s	Z	N Genes	Approach
1	0.23	0.00	-0.89	5433	standard
10	0.71	0.00	0.51	2513	standard
11	0.14	0.00	-0.85	3517	standard
12	0.55	0.00	0.11	3201	standard
13	0.55	0.00	0.10	1165	standard
14	0.42	0.00	-0.08	2022	standard
15	0.67	0.00	0.17	1971	standard
16	0.17	0.00	-1.10	2761	standard
17	0.05	0.00	-1.45	3383	standard
18	0.65	0.00	0.35	970	standard
19	0.04	0.00	-1.56	4043	standard
2	0.77	0.00	0.59	3960	standard
20	0.53	0.00	0.18	1884	standard
21	0.07	0.00	-1.35	643	standard
22	0.51	0.00	0.20	1552	standard
3	0.93	0.00	1.41	3362	standard
4	0.72	0.00	0.42	2297	standard
5	0.83	0.00	0.78	2787	standard
6	0.88	0.00	1.19	2691	standard
7	0.65	0.00	0.50	2848	standard
8	0.58	0.00	0.10	2202	standard

Chromosome	P	s	Z	N Genes	Approach
9	0.39	0.00	-0.36	2466	standard
X	0.45	0.00	-0.23	2395	standard
Y	0.66	0.00	-0.15	55	standard

Table S5. Prenatal Enrichment Null Model Statistics:

We assessed prenatal cell specific enrichment of the genes on PLS1 (*56*). We used a median gene rank approach to analyse an enrichment of PLS1 for genes associated with specific cell clusters. We did so by matching for gene length. Specifically, we resampled genes from the Allen Human Brain Atlas (AHBA) until we found a subset of genes that were not significantly different in length ($P < 0.05$) from the empirical gene list. Here, we show the results from the gene length matching algorithm, including the P-, s- and Z-value for the test of difference in gene length between the empirical gene list and the subset of resampled genes, the number of genes N Genes, to resample from, as well as which approach the algorithm converged on (see Supplementary Text “Matching for Gene Length” for details.)

Cluster	P	s	Z	N	Approach
ExN	0.55	0.00	0.08	364	standard
PgG2M	0.39	0.00	-0.54	1786	standard
OPC	0.91	0.00	1.47	2125	standard
End	0.81	0.00	0.84	2393	standard
ExDp2	0.96	0.00	1.87	1207	2 nearest neighbours
Per	0.48	0.00	-0.12	3388	standard
Mic	0.57	0.00	0.34	2982	standard
ExM	0.77	0.00	0.52	1006	standard
IP	0.41	0.00	-0.03	1100	standard
ExDp1	0.94	0.00	1.32	865	2 nearest neighbours
ExCal	0.89	0.00	1.28	1001	standard
InSST	0.57	0.00	0.25	748	standard
InCALB2	0.60	0.00	0.42	683	standard
oRG	0.78	0.00	0.90	2482	standard
PgS	0.42	0.00	-0.10	2200	standard
vRG	0.48	0.00	0.01	1925	standard

Table S6. Adult Cell Enrichment Null Model Statistics:

We assessed adult cell specific enrichment of the genes on PLS1 (*55*). We used a median gene rank approach to analyse an enrichment of PLS1 for genes associated with specific cell clusters. We did so by matching for gene length. Specifically, we resampled genes from the Allen Human Brain Atlas (AHBA) until we found a subset of genes that were not significantly different in length ($P < 0.05$) from the empirical gene list. Here, we show the results from the gene length matching algorithm, including the P-, s- and Z-value for the test of difference in gene length between the empirical gene list and the subset of resampled genes, the number of genes N Genes, to resample from, as well as which approach the algorithm converged on (see Supplementary Text “Matching for Gene Length” for details.)

Cluster	P	s	Z	N	Approach
Ex1	0.89	0.00	1.14	497	2 nearest neighbours
Ex2	0.95	0.00	1.53	320	2 nearest neighbours
Ex3a	0.59	0.00	0.38	738	without replacement
Ex3b	0.81	0.00	1.04	1851	standard
Ex3c	0.50	0.00	0.12	1113	standard
Ex3d	0.94	0.00	1.44	509	2 nearest neighbours
Ex3e	0.42	0.00	-0.34	1636	standard
Ex4	0.97	0.00	1.94	1134	standard
Ex5a	0.92	0.00	1.40	1500	standard
Ex5b	0.50	0.00	-0.08	645	without replacement
Ex6a	0.97	0.00	1.68	366	2 nearest neighbours
Ex6b	0.93	0.00	1.87	330	2 nearest neighbours
Ex8	0.94	0.00	1.28	499	2 nearest neighbours
In1a	0.92	0.00	1.43	426	2 nearest neighbours
In1b	0.97	0.00	1.95	370	2 nearest neighbours
In1c	0.93	0.00	2.04	299	2 nearest neighbours
In2	0.91	0.00	1.52	388	2 nearest neighbours
In3	0.82	0.00	1.30	306	2 nearest neighbours
In4a	0.91	0.00	1.13	349	2 nearest neighbours
In4b	0.95	0.00	2.24	476	2 nearest neighbours
In6a	0.97	0.00	1.62	332	2 nearest neighbours
In6b	0.49	0.00	0.02	630	without replacement
In7	0.91	0.00	1.28	372	2 nearest neighbours
In8	0.90	0.00	1.42	326	2 nearest neighbours
End	0.56	0.00	-0.13	245	standard

Cluster	P	s	Z	N	Approach
Ast	0.95	0.00	1.77	543	standard
Oli	0.84	0.00	0.74	704	standard
OPC	0.87	0.00	0.76	200	2 nearest neighbours
Mic	0.55	0.00	-0.03	300	standard

Table S7. MDD Enrichment Null Model Statistics:

We used a median gene rank approach to analyse an enrichment of PLS1 for genes associated with MDD risk genes (*57*). We did so by matching for gene length. Specifically, we resampled genes from the Allen Human Brain Atlas (AHBA) until we found a subset of genes that were not significantly different in length ($P < 0.05$) from the empirical gene list. Here, we show the results from the gene length matching algorithm, including the *P*-, *s*- and *Z*-value for the test of difference in gene length between the empirical gene list and the subset of resampled genes, the number of genes N Genes, to resample from, as well as which approach the algorithm converged on (see Supplementary Text "Matching for Gene Length" for details.)

P	s	Z	N	Approach
0.75	0.00	0.36	287	standard

Co-Location with Depression

Table S8. BioDep Sample Overview:

A total of N=96 subjects (50 MDD patients) subjects, balanced for age and sex were scanned at three MRI imaging centres.

Group	Sex *female*	μ Age	σ Age	μ FD	σ FD	Centre		
						Cambridge	Kings	Oxford
Control	27	35.5	7.5	0.08	0.05	36	6	4
MDD	29	36.8	7.1	0.01	0.05	37	8	5

Sensitivity Analyses

Table S9. NSPN Motion-Matched Sample Overview:

We found a subset of subjects such that there was no significant sex difference in framewise displacement (FD; $P > 0.05$). We resampled subjects within age-related strata and kept all scans from each single subject together, such that the original structure of the dataset was preserved.

Sex	#Scans	#Scanned			At Baseline			
		1	2	3	μ Age	σ Age	μ FD	σ FD
female	156	67	43	1	18.9	3.0	0.12	0.02
male	158	57	46	3	18.9	2.8	0.12	0.03

REFERENCES AND NOTES

1. E. R. Sowell, P. M. Thompson, A. W. Toga, Mapping changes in the human cortex throughout the span of life. *Neurosci.* **10**, 372–392 (2004).

2. J. N. Giedd, Structural magnetic resonance imaging of the adolescent brain. *Ann. N. Y. Acad. Sci.* **1021**, 77–85 (2004).

3. A. Raznahan, J. P. Lerch, N. Lee, D. Greenstein, G. L. Wallace, M. Stockman, L. Clasen, P. W. Shaw, J. N. Giedd, Patterns of coordinated anatomical change in human cortical development: A longitudinal neuroimaging study of maturational coupling. *Neuron* **72**, 873–884 (2011).

4. F. Váša, J. Seidlitz, R. Romero-Garcia, K. J. Whitaker, G. Rosenthal, P. E. Vértes, M. Shinn, A. Alexander-Bloch, P. Fonagy, R. J. Dolan, P. B. Jones, I. M. Goodyer, O. Sporns, E. T. Bullmore, Adolescent tuning of association cortex in human structural brain networks. *Cereb. Cortex* **28**, 281–294 (2018).

5. P. E. Vértes, T. Rittman, K. J. Whitaker, R. R.-García, F. Váša, M. G. Kitzbichler, K. Wagstyl, P. Fonagy, R. J. Dolan, P. B. Jones, I. M. Goodyer, E. T. Bullmore, Gene transcription profiles associated with inter-modular hubs and connection distance in human functional magnetic resonance imaging networks. *Philos. Trans. R. Soc. B Biol. Sci.* **371**, 20150362 (2016).

6. F. Váša, R. Romero-Garcia, M. G. Kitzbichler, J. Seidlitz, K. J. Whitaker, M. M. Vaghi, P. Kundu, A. X. Patel, P. Fonagy, R. J. Dolan, P. B. Jones, I. M. Goodyer, P. E. Vértes, E. T. Bullmore, Conservative and disruptive modes of adolescent change in human brain functional connectivity. *Proc. Natl. Acad. Sci. U.S.A.* **117**, 3248–3253 (2020).

7. D. A. Sturman, B. Moghaddam, The neurobiology of adolescence: Changes in brain architecture, functional dynamics, and behavioral tendencies. *Neurosci. Biobehav. Rev.* **35**, 1704–1712 (2011).

8. C. Faravelli, M. A. Scarpato, G. Castellini, C. L. Sauro, Gender differences in depression and anxiety: The role of age. *Psychiatry Res.* **210**, 1301–1303 (2013).

9. J. M. Cyranowski, E. Frank, E. Young, K. M. Shear, Adolescent onset of the gender difference in lifetime rates of major depression. *Arch. Gen. Psychiatry* **57**, 21 (2000).

10. B. L. Hankin, L. Y. Abramson, T. E. Moffitt, P. A. Silva, R. McGee, K. E. Angell, Development of depression from preadolescence to young adulthood: Emerging gender differences in a 10-year longitudinal study. *J. Abnorm. Psychol.* **107**, 128–140 (1998).

11. T. Paus, M. Keshavan, J. N. Giedd, Why do many psychiatric disorders emerge during adolescence? *Nat. Rev. Neurosci.* **9**, 947–957 (2008).

12. B. Biswal, F. Z. Yetkin, V. M. Haughton, J. S. Hyde, Functional connectivity in the motor cortex of resting human brain using echo-planar mri. *Magn. Reson. Med.* **34**, 537–541 (1995).

13. A. Fornito, A. Zalesky, E. T. Bullmore, *Fundamentals of Brain Network Analysis*, A. Fornito, A. Zalesky, E. T. Bullmore, Eds. (Academic Press, 2016).

14. K. J. Whitaker, P. E. Vértes, R. Romero-Garcia, F. Váša, M. Moutoussis, G. Prabhu, N. Weiskopf, M. F. Callaghan, K. Wagstyl, T. Rittman, R. Tait, C. Ooi, J. Suckling, B. Inkster, P. Fonagy, R. J. Dolan, P. B. Jones, I. M. Goodyer; The N. NSPN Consortium, E. T. Bullmore, Adolescence is associated with genomically patterned consolidation of the hubs of the human brain connectome. *Proc. Natl. Acad. Sci. U.S.A.* **113**, 9105–9110 (2016).

15. K. L. Mills, A.-L. Goddings, L. S. Clasen, J. N. Giedd, S.-J. Blakemore, The developmental mismatch in structural brain maturation during adolescence. *Dev. Neurosci.* **36**, 147–160 (2014).

16. D. A. Fair, A. L. Cohen, J. D. Power, N. U. F. Dosenbach, J. A. Church, F. M. Miezin, B. L. Schlaggar, S. E. Petersen, Functional brain networks develop from a “local to distributed” organization. *PLoS Comput. Biol.* **5**, e1000381 (2009).

17. D. A. Fair, N. U. F. Dosenbach, J. A. Church, A. L. Cohen, S. Brahmbhatt, F. M. Miezin, D. M. Barch, M. E. Raichle, S. E. Petersen, B. L. Schlaggar, Development of distinct control networks through segregation and integration. *Proc. Natl. Acad. Sci. U.S.A.* **104**, 13507–13512 (2007).

18. N. U. F. Dosenbach, B. Nardos, A. L. Cohen, D. A. Fair, J. D. Power, J. A. Church, S. M. Nelson, G. S. Wig, A. C. Vogel, C. N. L.-Schlaggar, K. A. Barnes, J. W. Dubis, E. Feczko, R. S. Coalson, J. R. Pruett, D. M. Barch, S. E. Petersen, B. L. Schlaggar, Prediction of individual brain maturity using fMRI. *Science* **329**, 1358–1361 (2010).

19. J. D. Power, K. A. Barnes, A. Z. Snyder, B. L. Schlaggar, S. E. Petersen, Spurious but systematic correlations in functional connectivity MRI networks arise from subject motion. *Neuroimage* **59**, 2142–54 (2012).

20. T. D. Satterthwaite, D. H. Wolf, J. Loughead, K. Ruparel, M. A. Elliott, H. Hakonarson, R. C. Gur, R. E. Gur, Impact of in-scanner head motion on multiple measures of functional connectivity: Relevance for studies of neurodevelopment in youth. *Neuroimage* **60**, 623–632 (2012).

21. T. Satterthwaite, D. H. Wolf, K. Ruparel, G. Erus, M. A. Elliott, S. B. Eickhoff, E. D. Gennatas, C. Jackson, K. Prabhakaran, A. Smith, H. Hakonarson, R. Verma, C. Davatzikos, R. E. Gur, R. C. Gur, Heterogeneous impact of motion on fundamental patterns of developmental changes in functional connectivity during youth. *Neuroimage* **83**, 45–57 (2013).

22. J. D. Power, M. Plitt, S. J. G. P. Kundu, V. Voon, P. A. B. A. Martin, Ridding fMRI data of motion-related influences: Removal of signals with distinct spatial and physical bases in multiecho data. *Proc. Natl. Acad. Sci. U.S.A.* **115**, E2105–E2114 (2018).

23. K. Murphy, R. M. Birn, D. A. Handwerker, T. B. Jones, P. A. Bandettini, The impact of global signal regression on resting state correlations: Are anti-correlated networks introduced? *Neuroimage* **44**, 893–905 (2009).

24. M. L. Schölvinck, A. Maier, F. Q. Ye, J. H. Duyn, D. A. Leopold, Neural basis of global resting-state fMRI activity. *Proc. Natl. Acad. Sci. U.S.A.* **107**, 10238–10243 (2010).

25. S. Maknojia, N. W. Churchill, T. A. Schweizer, S. J. Graham, Resting state fMRI: Going through the motions. *Front. Neurosci.* **13**, 825 (2019).

26. M. Filippi, P. Valsasina, P. Misci, A. Falini, G. Comi, M. A. Rocca, The organization of intrinsic brain activity differs between genders: A resting-state fMRI study in a large cohort of young healthy subjects. *Hum. Brain Mapp.* **34**, 1330–1343 (2013).

27. D. Tomasi, N. D. Volkow, Aging and functional brain networks. *Mol. Psychiatry* **17**, 549–558 (2012).

28. E. A. Allen, E. B. Erhardt, E. Damaraju, W. Gruner, J. M. Segall, R. F. Silva, M. Havlicek, S. Rachakonda, J. Fries, R. Kalyanam, A. M. Michael, A. Caprihan, J. A. Turner, T. Eichele, S. Adelsheim, A. D. Bryan, J. Bustillo, V. P. Clark, S. W. F. Ewing, F. Filbeyand, C. C. Ford, K. Hutchison, R. E. Jung, K. A. Kiehl, P. Kodituwakku, Y. M. Komesu, A. R. Mayer, G. D. Pearlson, J. P. Phillips, J. R. Sadek, M. Stevens, U. Teuscher, R. J. Thoma, V. D. Calhoun, A baseline for the multivariate comparison of resting-state networks. *Front. Syst. Neurosci.* **5**, 2 (2011).

29. B. B. Biswal, M. Mennes, X.-N. Zuo, S. Gohel, C. Kelly, S. M. Smith, C. F. Beckmann, J. S. Adelstein, R. L. Buckner, S. Colcombe, A.-M. Dogonowski, M. Ernst, D. Fair, M. Hampson, M. J. Hoptman, J. S. Hyde, V. J. Kiviniemi, R. Kötter, S.-J. Li, C.-P. Lin, M. J. Lowe, C. Mackay, D. J. Madden, K. H. Madsen, D. S. Margulies, H. S. Mayberg, K. McMahon, C. S. Monk, S. H. Mostofsky, B. J. Nagel, J. J. Pekar, S. J. Pekar, S. E. Petersen, V. Riedl, S. A. R. B. Rombouts, B. Rypma, B. L. Schlaggar, S. Schmidt, R. D. Seidler, G. J. Siegle, C. Sorg, G.-J. Teng, J. Veijola, A. Villringer, M. Walter, L. Wang, X.-C. Weng, S. Whitfield-Gabrieli, P. Williamson, C. Windischberger, Y.-F. Zang, H.-Y. Zhang, F. X. Castellanos, M. P. Milham, Toward discovery science of human brain function. *Proc. Natl. Acad. Sci. U.S.A.* **107**, 4734–4739 (2010).

30. R. L. Bluhm, E. A. Osuch, R. A. Lanius, K. Boksman, R. W. J. Neufeld, J. Théberge, P. Williamson, Default mode network connectivity: Effects of age, sex, and analytic approach. *Neuroreport* **19**, 887–891 (2008).

31. D. Scheinost, E. S. Finn, F. Tokoglu, X. Shen, X. Papademetris, M. Hampson, T. R. Constable, Sex differences in normal age trajectories of functional brain networks. *Hum. Brain Mapp.* **36**, 1524–1535 (2015).

32. I. Weissman-Fogel, M. Moayedi, K. S. Taylor, G. Pope, K. D. Davis, Cognitive and default-mode resting state networks: Do male and female brains "rest" differently? *Hum. Brain Mapp.* **31**, 1713–26 (2010).

33. G. Alarcón, A. Cservenka, M. D. Rudolph, D. A. Fair, B. J. Nagel, Developmental sex differences in resting state functional connectivity of amygdala sub-regions. *Neuroimage* **115**, 235–244 (2015).

34. L. A. Kilpatrick, D. H. Zald, J. V. Pardo, L. F. Cahill, Sex-related differences in amygdala functional connectivity during resting conditions. *Neuroimage* **30**, 452–461 (2006).

35. C. Zhang, N. D. Cahill, M. R. Arbabshirani, T. White, S. A. Baum, A. Michael, Sex and age effects of functional connectivity in early adulthood. *Brain Connect.* **6**, 700–713 (2016).

36. C. Zhang, C. C. Dougherty, S. A. Baum, T. White, A. M. Michael, Functional connectivity predicts gender: Evidence for gender differences in resting brain connectivity. *Hum. Brain Mapp.* **39**, 1765–1776 (2018).

37. R. Casanova, C. T. Whitlow, B. Wagner, M. A. Espeland, J. A. Maldjian, Combining graph and machine learning methods to analyze differences in functional connectivity across sex. *Open Neuroimaging J.* **6**, 1–9 (2012).

38. B. Kiddle, B. Inkster, G. Prabhu, M. Moutoussis, K. J. Whitaker, E. T. Bullmore, R. J. Dolan, P. Fonagy, I. M. Goodyer, P. B. Jones, Cohort profile: The NSPN 2400 Cohort: A developmental sample supporting the Wellcome trust NeuroScience in psychiatry network. *Int. J. Epidemiol.* **47**, 18–19g (2017).

39. M. G. Kitzbichler, A. R. Aruldass, G. J. Barker, T. C. Wood, N. G. Dowell, S. A. Hurley, J. McLean, M. Correia, C. Clarke, L. Pointon, J. Cavanagh, P. Cowen, C. Pariante, M. Cercignani; Neuroimmunology of mood disorders and Alzheimer's disease (NIMA) Consortium, E. T. Bullmore, N. A. Harrison, Peripheral inflammation is associated with micro-structural and functional connectivity changes in depression-related brain networks. *Mol. Psychiatry* **26**, 7346–7354 (2021).

40. A. R. Aruldass, M. G. Kitzbichler, S. E. Morgan, S. Lim, M.-E. Lynall, L. Turner, P. Vertes, J. Cavanagh, P. Cowen, C. M. Pariante, N. A. Harrison, E. T. Bullmore, Dysconnectivity of a brain functional network was associated with blood inflammatory markers in depression. *Brain Behav. Immun.* **98**, 299–309 (2021).

41. M. S. Çetin, F. Christensen, C. C. Abbott, J. M. Stephen, A. R. Mayer, J. M. Cañive, J. R. Bustillo, G. D. Pearlson, V. D. Calhoun, Thalamus and posterior temporal lobe show greater inter-network connectivity at rest and across sensory paradigms in schizophrenia. *Neuroimage* **97**, 117–126 (2014).

42. S. E. Morgan, J. Young, A. X. Patel, K. J. Whitaker, C. Scarpazza, T. van Amelsvoort, M. Marcelis, J. van Os, G. Donohoe, D. Mothersill, A. Corvin, C. Arango, A. Mechelli, M. van den Heuvel, R. S. Kahn, P. McGuire, M. Brammer, E. T. Bullmore, Functional magnetic resonance imaging connectivity accurately distinguishes cases with psychotic disorders from healthy controls, based on cortical features associated with brain network development. *Biol. Psychiatry: Cogn. Neurosci. Neuroimaging.* **6**, 1125–1134 (2021).

43. T. S. W. G. of the P. G. Consortium, S. Ripke, J. T. Walters, M. C. O'Donovan, Mapping genomic loci prioritises genes and implicates synaptic biology in schizophrenia. medRxiv (2020), p. 2020.09.12.20192922; doi:10.1101/2020.09.12.20192922.

44. A. X. Patel, P. Kundu, M. Rubinov, P. S. Jones, P. E. Vértes, K. D. Ersche, J. Suckling, E. T. Bullmore, A wavelet method for modeling and despiking motion artifacts from resting-state fMRI time series. *Neuroimage* **95**, 287–304 (2014).

45. B. T. Yeo, F. M. Krienen, J. Sepulcre, M. R. Sabuncu, D. Lashkari, M. Hollinshead, J. L. Roffman, J. W. Smoller, L. Zöllei, J. R. Polimeni, B. Fischl, H. Liu, R. L. Buckner, The organization of the human cerebral cortex estimated by intrinsic functional connectivity. *J. Neurophysiol.* **106**, 1125–1165 (2011).

46. V. J. Sydnor, B. Larsen, D. S. Bassett, A. Alexander-Bloch, D. A. Fair, C. Liston, A. P. Mackey, M. P. Milham, A. Pines, D. R. Roalf, J. Seidlitz, T. Xu, A. Raznahan, T. D. Satterthwaite,

Neurodevelopment of the association cortices: Patterns, mechanisms, and implications for psychopathology. *Neuron* **109**, 2820–2846 (2021).

47. T. Yarkoni, R. A. Poldrack, T. E. Nichols, D. C. V. Essen, T. D. Wager, Large-scale automated synthesis of human functional neuroimaging data. *Nat. Methods* **8**, 665–670 (2011).

48. S. E. Morgan, J. Seidlitz, K. J. Whitaker, R. Romero-Garcia, N. E. Clifton, C. Scarpazza, T. van Amelsvoort, M. Marcelis, J. van Os, G. Donohoe, D. Mothersill, A. Corvin, A. Pocklington, A. Raznahan, P. McGuire, P. E. Vértes, E. T. Bullmore, Cortical patterning of abnormal morphometric similarity in psychosis is associated with brain expression of schizophrenia-related genes. *Proc. Natl. Acad. Sci. U.S.A.* **116**, 9604–9609 (2019).

49. M. J. Hawrylycz, E. S. Lein, A. L. Guillozet-Bongaarts, E. H. Shen, L. Ng, J. A. Miller, L. N. van de Lagemaat, K. A. Smith, A. Ebbert, Z. L. Riley, C. Abajian, C. F. Beckmann, A. Bernard, D. Bertagnolli, A. F. Boe, P. M. Cartagena, M. Mallar Chakravarty, M. Chapin, J. Chong, R. A. Dalley, B. D. Daly, C. Dang, S. Datta, N. Dee, T. A. Dolbeare, V. Faber, D. Feng, D. R. Fowler, J. Goldy, B. W. Gregor, Z. Haradon, D. R. Haynor, J. G. Hohmann, S. Horvath, R. E. Howard, A. Jeromin, J. M. Jochim, M. Kinnunen, C. Lau, E. T. Lazarz, C. Lee, T. A. Lemon, L. Li, Y. Li, J. A. Morris, C. C. Overly, P. D. Parker, S. E. Parry, M. Reding, J. J. Royall, J. Schulkin, P. A. Sequeira, C. R. Slaughterbeck, S. C. Smith, A. J. Sodt, S. M. Sunkin, B. E. Swanson, M. P. Vawter, D. Williams, P. Wohnoutka, H Ronald Zielke, D. H. Geschwind, P. R. Hof, S. M. Smith, C. Koch, S. G. N. Grant, A. R. Jones, An anatomically comprehensive atlas of the adult human brain transcriptome. *Nature* 391–399 (2012).

50. H. E. O'Brien, E. Hannon, A. R. Jeffries, W. Davies, M. J. Hill, R. J. Anney, M. C. O'Donovan, J. Mill, N. J. Bray, Sex differences in gene expression in the human fetal brain. bioRxiv 10.1101/483636 [Preprint]. 2019.

51. M. Oliva, M. Muñoz-Aguirre, S. Kim-Hellmuth, V. Wucher, A. D. H. Gewirtz, D. J. Cotter, P. Parsana, S. Kasela, B. Balliu, A. Viñuela, S. E. Castel, P. Mohammadi, F. Aguet, Y. Zou, E. A. Khramtsova, A. D. Skol, D. Garrido-Martín, F. Reverter, A. Brown, P. Evans, E. R. Gamazon, A. Payne, R. Bonazzola, A. N. Barbeira, A. R. Hamel, A. Martinez-Perez, J. M. Soria; Gte. Consortium, B. L. Pierce, M. Stephens, E. Eskin, E. T. Dermitzakis, A. V. Segrè, H. K. Im, B. E.

Engelhardt, K. G. Ardlie, S. B. Montgomery, A. J. Battle, T. Lappalainen, R. Guigó, B. E. Stranger, The impact of sex on gene expression across human tissues. *Science* **369**, aba3066 (2020).

52. T. Tukiainen, A. C. Villani, A. Yen, M. A. Rivas, J. L. Marshall, R. Satij, M. Aguirre, L. Gauthier, M. Fleharty, A. Kirby, B. B. Cummings, S. E. Castel, K. J. Karczewski, F. Aguet, A. Byrnes; the Gte. Consortium, T. Lappalainen, A. Regev, K. G. Ardlie, N. Hacohen, D. G. MacArthur, Landscape of X chromosome inactivation across human tissues. *Nature* **550**, 244–248 (2017).

53. Y. Zhu, A. M. M. Sousa, T. Gao, M. Skarica, M. Li, G. Santpere, P. Esteller-Cucala, D. Juan, L. Ferrández-Peral, F. O. Gulden, M. Yang, D. J. Miller, T. Marques-Bonet, Y. I. Kawasawa, H. Zhao, N. Sestan, Spatiotemporal transcriptomic divergence across human and macaque brain development. *Science* **362**, eaat8077 (2018).

54. X. Xu, A. B. Wells, D. R. O'Brien, A. Nehorai, J. D. Dougherty, Cell type-specific expression analysis to identify putative cellular mechanisms for neurogenetic disorders. *J. Neurosci.* **34**, 1420–1431 (2014).

55. N. Lake, S. Chen, B. Sos, J. Fan, G. E. Kaeser, Y. C. Yung, T. E. Duong, D. Gao, J. Chun, P. V. Kharchenko, K. Zhang, Integrative single-cell analysis of transcriptional and epigenetic states in the human adult brain. *Nat. Biotechnol.* **36**, 70–80 (2018).

56. D. Polioudakis, L. de la Torre-Ubieta, J. Langerman, A. G. Elkins, X. Shi, J. L. Stein, C. K. Vuong, S. Nichterwitz, M. Gevorgian, C. K. Opland, D. Lu, W. Connell, E. K. Ruzzo, J. K. Lowe, T. Hadzic, F. I. Hinz, S. Sabri, W. E. Lowry, M. B. Gerstein, K. Plath, D. H. Geschwind, A single-cell transcriptomic atlas of human neocortical development during Mid-gestation. *Neuron* **103**, 785–801.e8 (2019).

57. M. Li, G. Santpere, Y. I. Kawasawa, O. V. Evgrafov, F. O. Gulden, S. Pochareddy, S. M. Sunkin, Z. Li, Y. Shin, Y. Zhu, A. M. M. Sousa, D. M. Werling, R. R. Kitchen, H. J. Kang, M. Pletikos, J. Choi, S. Muchnik, X. Xu, D. Wang, B. L.-Galdos, S. Liu, P. G.-Rodríguez, H. Won, C. A. de Leeuw, A. F. Pardiñas, B. S. Consortium; Psych ENCODE Consortium, Psych ENCODE Developmental Subgroup, M. Hu, F. Jin, Y. Li, M. J. Owen, M. C O'Donovan, J. T. R. Walters, D.

Posthuma, M. A. Reimers, P. Levitt, D. R. Weinberger, T. M. Hyde, J. E. Kleinman, D. H. Geschwind, M. J. Hawrylycz, M. W. State, S. J. Sanders, P. F. Sullivan, M. B. Gerstein, E. S. Lein, J. A. Knowles, N. Sestan, Integrative functional genomic analysis of human brain development and neuropsychiatric risks. *Science* **362**, eaat7615 (2018).

58. N. R. Wray, S. Ripke, M. Mattheisen, M. Trzaskowski, E. M. Byrne, A. Abdellaoui, M. J. Adams, E. Agerbo, T. M. Air, T. M. F. Andlauer, S.-A. Bacanu, M. B.-Hansen, A. F. T. Beekman, T. B. Bigdeli, E. B. Binder, D. R. H. Blackwood, J. Bryois, H. N. Buttenschøn, J. B.-Grauholm, N. Cai, E. Castelao, J. H. Christensen, T.-K. Clarke, J. I. R. Coleman, L. C.-Conde, B. Couvy-Duchesne, N. Craddock, G. E. Crawford, C. A. Crowley, H. S. Dashti, G. Davies, I. J. Deary, F. Degenhardt, E. M. Derks, N. Direk, C. V. Dolan, E. C. Dunn, T. C. Eley, N. Eriksson, V. Escott-Price, F. H. F. Kiadeh, H. K. Finucane, A. J. Forstner, J. Frank, H. A. Gaspar, M. Gill, P. Giusti-Rodríguez, F. S. Goes, S. D. Gordon, J. Grove, L. S. Hall, E. Hannon, C. S. Hansen, T. F. Hansen, S. Herms, I. B. Hickie, P. Hoffmann, G. Homuth, C. Horn, J.-J. Hottenga, D. M. Hougaard, M. Hu, C. L. Hyde, M. Ising, R. Jansen, F. Jin, E. Jorgenson, J. A. Knowles, I. S. Kohane, J. Kraft, W. W. Kretzschmar, J. Krogh, Z. Kutalik, J. M. Lane, Y. Li, Y. Li, P. A. Lind, X. Liu, L. Lu, D. J. Mac Intyre, D. F. Mac Kinnon, R. M. Maier, W. Maier, J. Marchini, H. Mbarek, P. M. Grath, P. M. Guffin, S. E. Medland, D. Mehta, C. M. Middeldorp, E. Mihailov, Y. Milaneschi, L. Milani, J. Mill, F. M. Mondimore, G. W. Montgomery, S. Mostafavi, N. Mullins, M. Nauck, B. Ng, M. G. Nivard, D. R. Nyholt, P. F. O'Reilly, H. Oskarsson, M. J. Owen, J. N. Painter, C. B. Pedersen, M. G. Pedersen, R. E. Peterson, E. Pettersson, W. J. Peyrot, G. Pistis, D. Posthuma, S. M. Purcell, J. A. Quiroz, P. Qvist, J. P. Rice, B. P. Riley, M. Rivera, S. S. Mirza, R. Saxena, R. Schoevers, E. C. Schulte, L. Shen, J. Shi, S. I. Shyn, E. Sigurdsson, G. B. C. Sinnamon, J. H. Smit, D. J. Smith, H. Stefansson, S. Steinberg, C. A. Stockmeier, F. Streit, J. Strohmaier, K. E. Tansey, H. Teismann, A. Teumer, W. Thompson, P. A. Thomson, T. E. Thorgeirsson, C. Tian, M. Traylor, J. Treutlein, V. Trubetskoy, A. G. Uitterlinden, D. Umbricht, S. Van der Auwera, A. M. van Hemert, A. Viktorin, P. M. Visscher, Y. Wang, B. T. Webb, S. M. Weinsheimer, J. Wellmann, G. Willemsen, S. H. Witt, Y. Wu, H. S. Xi, J. Yang, F. Zhang; eQTLGen, andMe, V. Arolt, B. T. Baune, K. Berger, D. I. Boomsma, S. Cichon, U. Dannlowski, E. C. J. de Geus, J. R. De Paulo, E. Domenici, K. Domschke, T. Esko, H. J. Grabe, S. P. Hamilton, C. Hayward, A. C. Heath, D. A. Hinds, K. S. Kendler, S. Kloiber, G. Lewis, Q. S. Li, S. Lucae, P. F. A. Madden, P. K. Magnusson, N. G.

Martin, A. M. Mc Intosh, A. Metspalu, O. Mors, P. B. Mortensen, B. M.-Myhsok, M. Nordentoft, M. M. Nöthen, M. C. O'Donovan, S. A. Paciga, N. L. Pedersen, B. W. J. H. Penninx, R. H. Perlis, D. J. Porteous, J. B. Potash, M. Preisig, M. Rietschel, C. Schaefer, T. G. Schulze, J. W. Smoller, K. Stefansson, H. Tiemeier, R. Uher, H. Völzke, M. M. Weissman, T. Werge, A. R. Winslow, C. M. Lewis, D. F. Levinson, G. Breen, A. D. Børglum, P. F. Sullivan; Major depressive disorder working group of the psychiatric genomics consortium, Genome-wide association analyses identify 44 risk variants and refine the genetic architecture of major depression. *Nat. Genet.* **50**, 668–681 (2018).

59. N. Y. A. Sey, B. Hu, W. Mah, H. Fauni, J. C. McAfee, P. Rajarajan, K. J. Brennand, S. Akbarian, H. Won, A computational tool (H-MAGMA) for improved prediction of brain-disorder risk genes by incorporating brain chromatin interaction profiles. *Nat. Neurosci.* **23**, 583–593 (2020).

60. S. L. Edwards, J. Beesley, J. D. French, A. M. Dunning, Beyond GWASs: Illuminating the dark road from association to function. *Am. J. Hum. Genet.* **93**, 779–797 (2013).

61. C. Sanchis-Segura, M. V. Ibañez-Gual, N. Aguirre, C. Forn, Effects of different intracranial volume correction methods on univariate sex differences in grey matter volume and multivariate sex prediction. *Sci. Rep.* **10**, 12953 (2020).

62. K. L. Mills, A. L. Goddings, M. M. Herting, R. Meuwese, S. J. Blakemore, E. A. Crone, R. E. Dahl, B. Güroğlu, A. Raznahan, E. R. Sowell, C. K. Tamnes, Structural brain development between childhood and adulthood: Convergence across four longitudinal samples. *Neuroimage* **141**, 273–281 (2016).

63. D. Witvliet, B. Mulcahy, J. K. Mitchell, Y. Meirovitch, D. R. Berger, Y. Wu, Y. Liu, W. X. Koh, R. Parvathala, D. Holmyard, R. L. Schalek, N. Shavit, A. D. Chisholm, J. W. Lichtman, A. D. T. Samuel, M. Zhen, Connectomes across development reveal principles of brain maturation. *Nature* **596**, 257–261 (2021).

64. C. H. Phoenix, R. W. Goy, A. A. Gerall, W. C. Young, Organizing action of prenatally administered testosterone propionate on the tissue mediating mating behavior in the female guinea pig. *Endocrinology* **65**, 369–382 (1959).

65. L. Carruth, I. Reisert, A. Arnold, Sex chromosome genes directly affect brain sexual differentiation. *Nat. Neurosci.* **5**, 933–4 (2002).

66. A. P. Arnold, J. Xu, W. Grisham, X. Chen, Y.-H. Kim, Y. Itoh, Minireview: Sex chromosomes and brain sexual differentiation. *Endocrinology* **145**, 1057–1062 (2004).

67. M. M. McCarthy, A. P. Arnold, Reframing sexual differentiation of the brain. *Nat. Neurosci.* **14**, 677–83 (2011).

68. K. R. Cullen, D. G. Gee, B. Klimes-Dougan, V. Gabbay, L. Hulvershorn, B. A. Mueller, J. Camchong, C. J. Bell, A. Houri, S. Kumra, K. O. Lim, F. X. Castellanos, M. P. Milham, A preliminary study of functional connectivity in comorbid adolescent depression. *Neurosci. Lett.* **460**, 227–231 (2009).

69. C. G. Connolly, J. Wu, T. C. Ho, F. Hoeft, O. Wolkowitz, S. Eisendrath, G. Frank, R. Hendren, J. E. Max, M. P. Paulus, S. F. Tapert, D. Banerjee, A. N. Simmons, T. T. Yang, Resting-state functional connectivity of subgenual anterior cingulate cortex in depressed adolescents. *Biol. Psychiatry* **74**, 898–907 (2013).

70. K. M. Anderson, M. A. Collins, R. Kong, K. Fang, J. Li, T. He, A. M. Chekroud, B. T. T. Yeo, A. J. Holmes, Convergent molecular, cellular, and cortical neuroimaging signatures of major depressive disorder. *Proc. Natl. Acad. Sci. U.S.A.* **117**, 25138–25149 (2020).

71. B. Ji, K. K. Higa, J. R. Kelsoe, X. Zhou, Over-expression of XIST, the master gene for X chromosome inactivation, in females with major affective disorders. *EBioMedicine* **2**, 909–918 (2015).

72. M. L. Elliott, A. Romer, A. R. Knodt, A. R. Hariri, A connectome-wide functional signature of transdiagnostic risk for mental illness. *Biol. Psychiatry* **84**, 452–459 (2018).

73. M. J. Gandal, J. R. Haney, N. N. Parikshak, V. Leppa, G. Ramaswami, C. Hartl, A. J. Schork, V. Appadurai, A. Buil, T. M. Werge, C. Liu, K. P. White, CommonMind Consortium; PsychENCODE Consortium, iPSYCH-BROAD Working Group, S. Horvath, D. H. Geschwind,

Shared molecular neuropathology across major psychiatric disorders parallels polygenic overlap. *Science* **359**, 693–697 (2018).

74. A. N. Kaczkurkin, A. Raznahan, T. D. Satterthwaite, Sex differences in the developing brain: Insights from multimodal neuroimaging. *Neuropsychopharmacology* **44**, 71–85 (2019).

75. L. Cahill, Why sex matters for neuroscience. *Nat. Rev. Neurosci.* **7**, 477–484 (2006).

76. L. Eliot, A. Ahmed, H. Khan, J. Patel, Dump the “dimorphism”: Comprehensive synthesis of human brain studies reveals few male-female differences beyond size. *Neurosci. Biobehav. Rev.* **125**, 667–697 (2021).

77. A. Arnatkeviciute, B. D. Fulcher, M. A. Bellgrove, A. Fornito, Where the genome meets the connectome: Understanding how genes shape human brain connectivity. *Neuroimage* **244**, 118570 (2021).

78. B. D. Fulcher, A. Arnatkeviciute, A. Fornito, Overcoming false-positive gene-category enrichment in the analysis of spatially resolved transcriptomic brain atlas data. *Nat. Commun.* **12**, 2669 (2021).

79. A. Alexander-Bloch, A. Raznahan, E. Bullmore, J. Giedd, The convergence of maturational change and structural covariance in human cortical networks. *J. Neurosci.* **33**, 2889–2899 (2013).

80. N. A. Crossley, L. M. Alliende, T. Ossandon, C. P. Castañeda, A. González-Valderrama, J. Undurraga, M. Castro, S. Guinjoan, A. M. Díaz-Zuluaga, J. A. Pineda-Zapata, C. López-Jaramillo, F. Reyes-Madrigal, P. León-Ortíz, C. de la Fuente-Sandoval, L. S. Czepielewski, C. S. Gama, A. Zugman, A. Gadelha, A. Jackowski, R. Bressan, Imaging social and environmental factors as modulators of brain dysfunction: Time to focus on developing non-western societies. *Biol. Psychiatry: Cogn. Neurosci. Neuroimaging.* **4**, 8–15 (2019).

81. U. A. Tooley, D. S. Bassett, A. P. Mackey, Environmental influences on the pace of brain development. *Nat. Rev. Neurosci.* **22**, 372–384 (2021).

82. S. Yu, Uncovering the hidden impacts of inequality on mental health: A global study. *Transl. Psychiatry* **8**, 1–10 (2018).

83. P. Kundu, S. J. Inati, J. W. Evans, W.-M. Luh, P. A. Bandettini, Differentiating BOLD and non-BOLD signals in fMRI time series using multi-echo EPI. *Neuroimage* **60**, 1759–1770 (2012).

84. P. Kundu, N. D. Brenowitz, V. Voon, Y. Worbe, P. E. Vértes, S. J. Inati, Z. S. Saad, P. A. Bandettini, E. T. Bullmore, Integrated strategy for improving functional connectivity mapping using multiecho fMRI. *Proc. Natl. Acad. Sci. U.S.A.* **110**, 16187–92 (2013).

85. R. W. Cox, AFNI: Software for analysis and visualization of functional magnetic resonance neuroimages. *Comput. Biomed. Res.* **29**, 162–173 (1996).

86. M. F. Glasser, T. S. Coalson, E. C. Robinson, C. D. Hacker, J. Harwell, E. Yacoub, K. Ugurbil, J. Andersson, C. F. Beckmann, M. Jenkinson, S. M. Smith, D. C. V. Essen, A multi-modal parcellation of human cerebral cortex. *Nature* **536**, 171–178 (2016).

87. P. A. Filipek, C. Richelme, D. N. Kennedy, V. S. Caviness, The young adult human brain: An MRI-based morphometric analysis. *Cereb. Cortex* **4**, 344–360 (1994).

88. B. Fischl, D. H. Salat, E. Busa, M. Albert, M. Dieterich, C. Haselgrove, A. van der Kouwe, R. Killiany, D. Kennedy, S. Klaveness, A. Montillo, N. Makris, B. Rosen, A. M. Dale, Automated labeling of neuroanatomical structures in the human brain. *Cell Press* **33**, 341–355 (2002).

89. E. T. Bullmore, J. Fadili, V. Maxim, L. Şendur, B. Whitcher, J. Suckling, M. Brammer, M. Breakspear, Wavelets and functional magnetic resonance imaging of the human brain. *Neuroimage* **23**, S234–S249 (2004).

90. R. A. Fisher, Frequency distribution of the values of the correlation coefficient in samples from an indefinitely large population. *Biometrika* **10**, 507–521 (1915).

91. R. Paternoster, R. Brame, P. Mazerolle, A. Piquero, Using the correct statistical test for the equality of regression coefficients. *Crim.* **36**, 859–866 (1998).

92. J. Seidlitz, A. Nadig, S. Liu, R. A. I. Bethlehem, P. E. Vértes, S. E. Morgan, F. Váša, R. Romero-Garcia, F. M. Lalonde, L. S. Clasen, J. D. Blumenthal, C. Paquola, B. Bernhardt, K. Wagstyl, D. Polioudakis, L. de la Torre-Ubieta, D. H. Geschwind, J. C. Han, N. R. Lee, D. G. Murphy, E. T.

Bullmore, A. Raznahan, Transcriptomic and cellular decoding of regional brain vulnerability to neurogenetic disorders. *Nat. Commun.* **11**, 3358 (2020).

93. N. Weiskopf, J. Suckling, G. Williams, M. M. Correia, B. Inkster, R. Tait, C. Ooi, E. T. Bullmore, A. Lutti, Quantitative multi-parameter mapping of R1, PD(*), MT, and R2(*) at 3T: A multi-center validation. *Front. Neurosci.* **7**, 95 (2013).

94. M. Barth, J. R. Reichenbach, R. Venkatesan, E. M. M. E. Haacke, High-resolution, multiple gradient-echo functional MRI at 1.5 T. *Magn. Reson. Imaging* **17**, 321–329 (1999).

95. B. Fischl, M. I. Sereno, A. M. Dale, Cortical surface-based analysis: II: Inflation, flattening, and a surface-based coordinate system. *Neuroimage* **9**, 195–207 (1999).

96. Z. S. Saad, D. R. Glen, G. Chen, M. S. Beauchamp, R. Desai, R. W. Cox, A new method for improving functional-to-structural MRI alignment using local Pearson correlation. *Neuroimage* **44**, 839–848 (2009).

97. P. Reiss, L. Huang, Y.-H. Chen, L. Huo, T. Tarpey, M. Mennes, Massively parallel nonparametric regression, with an application to developmental brain mapping. *J. Comput. Graph. Stat.* **23**, 232–248 (2014).

98. R. D. Markello, B. Misic, Comparing spatial null models for brain maps. *Neuroimage* **236**, 118052 (2021).

99. A. Arnatkeviciute, B. D. Fulcher, A. Fornito, A practical guide to linking brain-wide gene expression and neuroimaging data. *Neuroimage* **189**, 353–367 (2019).

100. J. A. Miller, S.-L. Ding, S. M. Sunkin, K. A. Smith, L. Ng, A. Szafer, A. Ebbert, Z. L. Riley, J. J. Royall, K. Aiona, J. M. Arnold, C. Bennet, D. Bertagnolli, K. Brouner, S. Butler, S. Caldejon, A. Carey, C. Cuhaciyan, R. A. Dalley, N. Dee, T. A. Dolbeare, B. A. C. Facer, D. Feng, T. P. Fliss, G. Gee, J. Goldy, L. Gourley, B. W. Gregor, G. Gu, R. E. Howard, J. M. Jochim, C. L. Kuan, C. Lau, C.-K. Lee, F. Lee, T. A. Lemon, P. Lesnar, B. McMurray, N. Mastan, N. Mosqueda, T. Naluai-Cecchini, N.-K. Ngo, J. Nyhus, A. Oldre, E. Olson, J. Parente, P. D. Parker, S. E. Parry, A. Stevens, M. Pletikos, M. Reding, K. Roll, D. Sandman, M. Sarreal, S. Shapouri, N. V.

Shapovalova, E. H. Shen, N. Sjoquist, C. R. Slaughterbeck, M. Smith, A. J. Sodt, D. Williams, L. Zöllei, B. Fischl, M. B. Gerstein, D. H. Geschwind, I. A. Glass, M. J. Hawrylycz, R. F. Hevner, H. Huang, A. R. Jones, J. A. Knowles, P. Levitt, J. W. Phillips, N. Šestan, P. Wohnoutka, C. Dang, A. Bernard, J. G. Hohmann, E. S. Lein, Transcriptional landscape of the prenatal human brain. *Nature* **508**, 199–206 (2014).

101. B. A. Poser, M. J. Versluis, J. M. Hoogduin, D. G. Norris, BOLD contrast sensitivity enhancement and artifact reduction with multiecho EPI: Parallel-acquired inhomogeneity-desensitized fMRI. *Magn. Reson. Med.* **55**, 1227–1235 (2006).

102. A. X. Patel, E. T. Bullmore, A wavelet-based estimator of the degrees of freedom in denoised fMRI time series for probabilistic testing of functional connectivity and brain graphs. *Neuroimage* **142**, 14–26 (2016).

103. S. Achard, R. Salvador, B. Whitcher, J. Suckling, E. Bullmore, A resilient, low-frequency, small-world human brain functional network with highly connected association cortical hubs. *J. Neurosci.* **26**, 63–72 (2006).
